# The Transcriptome of *Leishmania major* Developmental Stages in Their Natural Sand Fly Vector

**DOI:** 10.1128/mBio.00029-17

**Published:** 2017-04-04

**Authors:** Ehud Inbar, V. Keith Hughitt, Laura A. L. Dillon, Kashinath Ghosh, Najib M. El-Sayed, David L. Sacks

**Affiliations:** a Laboratory of Parasitic Diseases, National Institute of Allergy and Infectious Diseases, National Institutes of Health, Bethesda, Maryland, USA; b Department of Cell Biology and Molecular Genetics, University of Maryland, College Park, Maryland, USA; c Center for Bioinformatics and Computational Biology, University of Maryland, College Park, Maryland, USA; Washington University School of Medicine

**Keywords:** Leishmania, sand fly, transcriptome

## Abstract

The life cycle of the *Leishmania* parasite in the sand fly vector involves differentiation into several distinctive forms, each thought to represent an adaptation to specific microenvironments in the midgut of the fly. Based on transcriptome sequencing (RNA-Seq) results, we describe the first high-resolution analysis of the transcriptome dynamics of four distinct stages of *Leishmania major* as they develop in a natural vector, *Phlebotomus duboscqi*. The early transformation from tissue amastigotes to procyclic promastigotes in the blood-fed midgut was accompanied by the greatest number of differentially expressed genes, including the downregulation of amastins, and upregulation of multiple cell surface proteins, sugar and amino acid transporters, and genes related to glucose metabolism and cell cycle progression. The global changes accompanying post-blood meal differentiation of procyclic promastigotes to the nectomonad and metacyclic stages were less extensive, though each displayed a unique signature. The transcriptome of nectomonads, which has not been studied previously, revealed changes consistent with cell cycle arrest and the upregulation of genes associated with starvation and stress, including autophagic pathways of protein recycling. Maturation to the infective, metacyclic stage was accompanied by changes suggesting preadaptation to the intracellular environment of the mammalian host, demonstrated by the amastigote-like profiles of surface proteins and metabolism-related genes. Finally, a direct comparison between sand fly-derived and culture-derived metacyclics revealed a reassuring similarity between the two forms, with the *in vivo* forms distinguished mainly by a stronger upregulation of transcripts associated with nutrient stress.

## IMPORTANCE

The life cycle of *Leishmania* parasites in the sand fly vector includes their growth and development as morphologically distinct forms of extracellular promastigotes found within the different microenvironments of the gut. Based on RNA-Seq, we provide here the first high-resolution, transcriptomic analysis of *Leishmania* insect stages during their cyclical development *in vivo*, from tissue amastigotes ingested with the blood meal to infective, metacyclic promastigotes that initiate infection in the mammalian host. The most extensive genetic reprogramming occurred during the early transformation of amastigotes to rapidly dividing procyclic promastigotes in the blood-fed midgut, with major changes in the abundance of mRNAs for surface proteins and metabolism. The post-blood meal-adapted nectomonad stage was characterized by the downregulation of cell cycle-related genes and the upregulation of stress- and starvation-related genes. Finally, the transcriptome of metacyclic promastigotes shifted to a more amastigote-like profile, suggesting their preadaptation to the intracellular host environment.

## INTRODUCTION

Leishmaniasis is the general name for a group of diseases caused by different species of the parasitic protozoan *Leishmania* that produce distinct clinical outcomes, ranging from localized, self-limiting cutaneous lesions, to more chronic forms of cutaneous and mucocutaneous disease, to visceral disease, which is fatal if untreated. According to the WHO there are around 1.3 million new cases of leishmaniasis around the world, with 20,000 to 30,000 deaths each year (http://www.who.int/mediacentre/factsheets/fs375/en/).

*Leishmania* parasites have a dimorphic life cycle, shifting between the alimentary tract of their sand fly vector as extracellular, flagellated promastigotes and the phagolysosomal vacuoles of their mammalian host mononuclear phagocytes as intracellular amastigotes. The ability of the parasite to adapt to these vastly different environments has been the focus of a number of studies comparing the transcriptomes, proteomes, and metabolomes of amastigotes and promastigotes during their transformation *in vitro* (1–6). More recent studies have addressed the differential gene expression levels associated with the maturation of promastigotes to the mammalian-infective, metacyclic stage, again using *in vitro* conditions (7). Finally, two recent studies have demonstrated differential gene expression between *L. infantum* promastigtoes in the anterior midgut of a natural vector, *Phlebotomus perniciosus*, versus those in stationary-phase culture (8) or versus intracellular amastigotes *in vitro* (9). To date, no studies have defined the genetic reprogramming associated with amastigote-to-promastigote transformation in the vector, nor have studies focused on the more complex series of promastigote developmental changes that accompany the maturation of transmissible infections *in vivo*.

Phlebotomine sand flies are the only known natural vectors of *Leishmania* spp. responsible for human disease (reviewed by Killick-Kendrick in 1999 [10]); they are essential to sustain the life cycle of this parasite. Female sand flies require a blood meal before they will lay eggs (10), and the gonotrophic cycle is defined as the period between blood meals during which the ova develop and are subsequently deposited. The sequence of physiological events that delineate a normal gonotrophic cycle, and that define the ecology of the *Leishmania* parasite in the vector, can be summarized as follows: when a sand fly takes a blood meal, the ingested blood passes through the food canal and esophagus into the posterior midgut via the stomodeal valve (SV), which regulates the flow of fluids into the gut. A peritrophic matrix (PM) is rapidly secreted by midgut cells to completely envelop the fresh blood meal (11, 12). The PM protects the midgut epithelial cells from damage by blood meal contents, but it remains permeable to the digestive enzymes induced by blood feeding (reviewed by Lehane in 1997 [13]); nutrients derived from the digesting blood meal are required for egg development, which become fully mature and deposited around the time that the blood meal remnants are excreted (10, 14). Depending on the sand fly species and ambient conditions, varied proportions of females survive oviposition and undergo multiple gonotrophic cycles, with each additional cycle requiring another blood meal, thus increasing the capacity of the vector to transmit *Leishmania* (10). During each gonotrophic cycle, females will continue to feed on sugar meals that serve as an energy source for the fly during the interval between blood meals. The sources of the sugars are typically plant sap, nectar, or aphid and coccid honeydew, which are all rich in sucrose (15–17). The sugar feeds are stored in the crop and diffuse into the thoracic midgut (15, 18).

Suprapylarian *Leishmania* spp. include all members of the genus with the exception of the *Vianna* subgenus, in which parasite development is confined to the midgut and the foregut. Some general aspects regarding the development of Suprapylarian species appear to be consistent (19): the infective blood meal containing *Leishmania* amastigotes is passed into the abdominal midgut, where the blood is quickly retained inside the PM. The transformation of amastigotes to promastigotes occurs within 12 to 18 h. These early transformed promastigotes are termed procyclics and appear as short, ovoid, and only slightly motile forms. For the next 36 to 60 h, rapid multiplication of procyclic promastigotes within the digesting blood meal continues, followed by their transformation to a long, slender, more actively motile form termed nectomonads. By 60 to 72 h, coincident with the excretion of the digested blood meal, tremendous numbers of nectomonads are found packed in the anterior portion of the abdominal midgut, with many attached via their flagella to the epithelial cell microvilli (19, 20). By days 7 to 10, the anterior migration of promastigotes to the region of the thoracic midgut and stomodeal valve proceeds until a massive accumulation of parasites behind the valve is achieved. This migration is associated with the transformation of nectomonads into short, actively dividing forms called leptomonads that produce a mucin-like substance termed promastigote secretory gel (PSG) in which the mass of promastigotes in the thoracic midgut are imbedded (21, 22). Broad forms termed haptomonads can be found in attachment to the cuticular surface of stomodeal valve (20). The final development phase is termed metacyclogensis, which refers to the differentiation to metacyclic promastigotes, thought to originate from leptomonads (21, 22), though their transformation directly from nectomonads cannot be ruled out. Metacyclic promastigotes are short, slender, highly motile, and unattached forms with a flagellum at least twice the length of the cell body, and they are never seen in division. They are believed to be the only forms egested into the skin when an infected fly takes a blood meal (23).

There are thus a number of promastigote developmental stages, and distinct microenvironments in the fly to which they are likely adapted, that are absent when *Leishmania* are axenically grown *in vitro*. We used transcriptome sequencing (RNA-Seq) to profile the transcriptomes of *L. major* during its transition from amastigotes to promastigotes in the vector, and also during its subsequent development as nectomonads and its maturation to the infective, metacyclic stage. Given that the vast majority of experimental studies are confined to use of cultured promastigotes, we also found it relevant to compare the transcriptomes of sand fly-derived and culture-derived metacyclics.

It should be noted that transcription profiles do not always reflect the true proteomic and metabolomic conditions, which is an inherent weakness in transcriptomic studies. Since proteomic and metabolomic studies are still technically challenging when working with such small amounts of starting material as can be obtained from sand fly midguts, RNA-Seq is currently the most useful approach to predict the genes that are associated with the development of the *Leishmania* parasite inside the vector.

## RESULTS AND DISCUSSION

### Experimental design.

**FIG 1:**
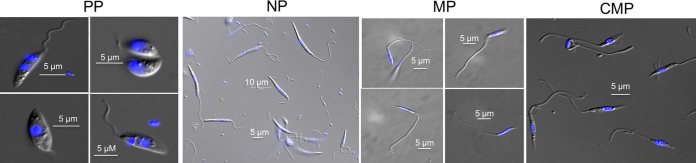
Morphological observations of the different *L. major* developmental stages. Light microscope images are of promastigotes recovered from sand flies on days 2 (PP), 4 (NP), and 15 (MP) postinfection and stained with Hoechst fluorescent dye. Culture-derived metacyclics (CMP) were purified from stationary-phase cultures of promastigotes growing in CM199.

*L. major* amastigotes were used to initiate infection in *P. duboscqi* sand flies, which are a natural vector for *L. major*. The flies were infected via artificial feeding through a chick skin membrane on mouse blood seeded with tissue-derived amastigotes prepared from the footpad lesions of BALB/c mice. Flies were dissected and midguts harboring homogeneous populations of procyclic (PP), nectomonad (NP), and metacyclic promastigotes (MP) were collected and pooled on days 2, 4, and 15 post-blood meal (PBM), respectively (Fig. 1). RNA was extracted from these samples, as well as from lesion-derived amastigotes (AM) and from culture-derived metacyclic promastigotes (CMP). These procedures were repeated to obtain duplicate, independent samples of each developmental form. Of note, midgut-derived leptomonads or haptomonads were not analyzed, because it is so far not possible to obtain homogenous populations of these *in vivo* forms.

A total of 1.9 × 10^8^ reads were generated across the 10 experimental samples after trimming 23.3% to 26.6% of the reads due to poor-quality sequences (see Table S1 in the supplemental material). In samples from replicate A, 76.4% of the high-quality reads in the RNA extracted from footpad-derived amastigotes mapped to the *L. major* genome. As expected, RNA extracted from the sand fly midguts contained a relatively low proportion of *L. major* sequences: 21.2% of the total midgut RNA sequences on day 2, 14.1% on day 4, and 14.1% on day 15. The rest of the reads mapped to the sand fly or other eukaryotic genomes, such as fungi, that may be normal constituents of the gut microbiota. In RNA extracted from culture-derived metacyclics, 87.2% of the reads mapped to the *L. major* genome. Similar numbers were observed for the RNA samples from replicate B (Table S1).

10.1128/mBio.00029-17.9 TABLE S1  RNA sequence reads from the different samples. Download TABLE S1, DOCX file, 0.1 MB. Copyright © 2017 Inbar et al. 2017 Inbar et al. This content is distributed under the terms of the Creative Commons Attribution 4.0 International license.

### Relationship between samples.

**FIG 2:**
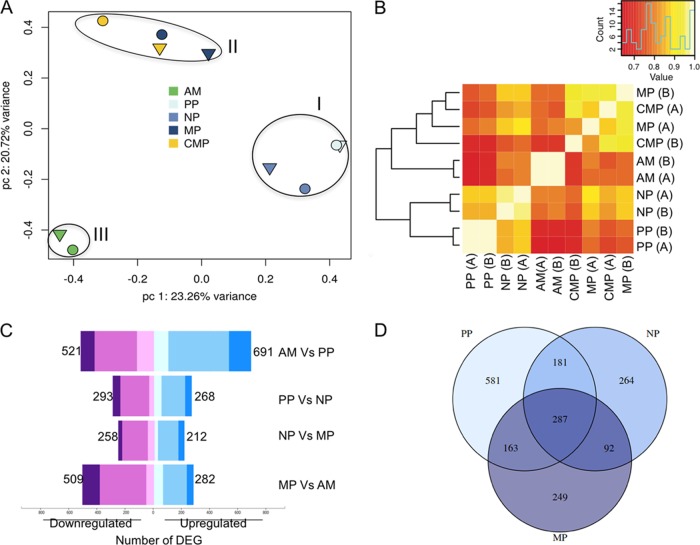
Global transcriptomic profiles of *L. major* parasites in the different developmental stages. RNA-Seq was performed on replicate samples of sand fly-derived procyclic (PP), nectomonad (NP), and metacyclic promastigotes (MP), purified culture-derived metacyclics (CMP), and footpad lesion-derived amastigotes (AM). (A) PCA results. The different colors indicate the different developmental stages in biological replicates A (triangles) and B (circles). (B) Heat map of hierarchical clustering analysis using the Pearson correlation, with correlation levels indicated by colors (inset). (C) Distribution of DEG between different sequential developmental stages *in vivo* are indicated in a bar graph. The box width depicts the number of DEG downregulated (purple) and upregulated (blue) at an adjusted *P* value of 0.05, with the total number of down- and upregulated genes shown. The color shading indicates the proportion of genes with at least 4-fold (dark), between 2- and 4-fold (medium), or 2-fold differential expression (light). (D) Venn diagram showing the differentially and commonly expressed mRNAs between AM and each of the sand fly promastigote stages.

Principal component analysis (PCA) (Fig. 2A; see also Fig. S1B and C in the supplemental material) and Pearson correlation heat maps (Fig. 2B) were used to study the relationship between the samples and to evaluate the reproducibility of the biological replicates. The PCA plot shows the top two principal components which explain most of the variance between samples in the data set, 23%, 21% and 15.49% for PC1, PC2, and PC3, respectively (see Fig. S1 for PC3). The analysis revealed a high degree of similarity between the duplicate samples, indicating a low batch effect with no clustering based on biological replicate (shapes) but rather on the developmental stage (color) (Fig. 2A; see also Fig. S1B and C). This was further supported by the scatterplots of normalized gene counts for each pair of biological replicates, showing high correlation values ranging from 0.79 to 0.95 (Fig. S1A). Therefore, no batch correction was applied in any further analysis. The data set could be divided into three distinct clusters: cluster I, grouping together procyclic and nectomonad promastigotes; cluster II, with both culture- and sand fly-derived metacyclics, and cluster III, with tissue-derived amastigotes. This observation suggested distinct variations in the transcriptome between the earlier stages of infection, namely, procyclics and nectomonads, and the mature metacyclic promastigote forms. This deviation was also observed with the Pearson correlation heat map (Fig. 2B), which separated MP from the earlier sand fly stages and clustered them more closely with tissue amastigotes. This suggests a transcriptomic preadaptation of the fully mature metacyclic forms to the intracellular environment that they will encounter following transmission by bite. Notably, both culture- and sand fly-derived metacyclics clustered together (cluster II), suggesting high similarity between the transcriptomes of both forms. However, slight separation between MP and CMP was observed when plotting PC1 versus PC3 and PC2 versus PC3 (Fig. S1B and C), suggesting that the transcriptomes of metacyclics derived under these two conditions are close but not identical.

10.1128/mBio.00029-17.7 FIG S1  Scatterplots indicating correlation of expression values across replicates and PCA plots of PC1 versus PC3 and PC2 versus PC3. (A) Correlation of expression values across replicates. For each of the five conditions considered, scatterplots show the Voom and quantile normalized log_2_ counts per million values for each pair of replicates. (B and C) PCA results, with plots of PC1 versus PC3 (B) and PC2 versus PC3 (C). The different colors indicate the different developmental stages in biological replicates A (triangles) and B (circles). Download FIG S1, TIF file, 0.3 MB. Copyright © 2017 Inbar et al. 2017 Inbar et al. This content is distributed under the terms of the Creative Commons Attribution 4.0 International license.

### Identification of differentially expressed genes between the four developmental stages.

A list of differentially expressed genes (DEG) with a *P* value of <0.05 was generated for each of the different pairwise comparisons of the developmental stages (see Data Set S1 for the complete list of DEG). The total number of DEG are summarized in Table S2; Fig. 2C shows DEG that are at least 2-fold or 4-fold different, and for only the comparison of stages that occur sequentially. A total of 1,212 DEG were associated with the transition from AM to PP, of which 691 and 521 were up- and downregulated in AM, respectively. Comparisons between the promastigote stages revealed smaller differences in mRNA abundance. We found 561 DEG between PP and NP, 470 between NP and MP, and 791 between MP and AM.

10.1128/mBio.00029-17.2 DATA SET S1  List of differentially expressed genes for each of the different pairwise comparisons of the developmental stages. Download DATA SET S1, XLS file, 0.8 MB. Copyright © 2017 Inbar et al. 2017 Inbar et al. This content is distributed under the terms of the Creative Commons Attribution 4.0 International license.

10.1128/mBio.00029-17.10 TABLE S2  Summary of differentially expressed genes between the different samples. Download TABLE S2, DOCX file, 0.04 MB. Copyright © 2017 Inbar et al. 2017 Inbar et al. This content is distributed under the terms of the Creative Commons Attribution 4.0 International license.

Venn diagrams were generated to reveal the unique and common mRNA abundance patterns between the different developmental stages (Fig. 2D; see also Fig. S2). AM had the highest number of unique genes in its expression profile, with 287 genes that were different from PP, NP, and MP (Fig. 2D). In contrast, 81 genes were uniquely up- or downregulated in PP, while 53 and 52 genes showed unique expression patterns in NP and MP, respectively (Fig. S2).

10.1128/mBio.00029-17.8 FIG S2  Venn diagrams for differentially and commonly expressed mRNAs between the developmental stages. Venn diagrams show differentially and commonly expressed mRNAs between *L. major* developmental forms in relation to PP (A), NP (B), and MP (C). Download FIG S2, TIF file, 0.2 MB. Copyright © 2017 Inbar et al. 2017 Inbar et al. This content is distributed under the terms of the Creative Commons Attribution 4.0 International license.

Of the 1,212 genes that were significantly up- or downregulated during transformation of AM to PP, 581 genes showed expression patterns that were unique to PP, while the differences between AM and NP and between AM and MP were significantly fewer, with 264 and 249 DEG, respectively (Fig. 2D). These observations are in line with the PCA and Pearson correlation analyses, suggesting that *in vivo* promastigotes become transcriptionally closer to amastigotes as they mature to the mammalian-infective, metacyclic stage. Overall, the major shift in the transcriptome was, as expected, between AM and the different sand fly stages. However, a unique signature was associated with each of the promastigote forms, validating that the distinctive cellular morphologies upon which their developmental stage designations have been based do in fact reflect distinct gene developmental programs.

Based on the DEG, we used Gene Ontology (GO) and KEGG enrichment analyses (see Materials and Methods) to identify the cellular processes associated with parasite development *in vivo* and culminating in their maturation into MP. Upregulation and downregulation of GO categories in pairwise comparisons between the different developmental stages are listed in Data Set S2. Five GO categories were downregulated during the differentiation from AM to PP, among which were categories related to antioxidant activity (GO:0016209), cysteine peptidase (GO:0008234), and DNA catabolism (GO:0006308) (Data Set S2). Eighteen GO categories were shown to be upregulated in PP compared to AM, including categories related to nucleosomes (GO:0000786), motility (GO:0031514), and synthesis of ATP (GO:0000275). Seven GO categories were downregulated in the transition from PP to NP, mainly related to the cell cycle (GO:0000786 and GO:0000776), and no significant upregulated GO categories were found. Only two groups were downregulated during the transition from NP to MP, and these were related to rRNA processing (GO:0006364 and GO:0032040). No significant upregulated GO categories were found in MP compared to NP. Fourteen GO categories were shown to be upregulated in MP compared to PP, with most being related to transport and homeostasis of nonessential amino acids, like proline, alanine, and glutamate (GO:0015193, GO:0022858, and GO:0002036), and to signal transduction (GO:0035556). Eight GO categories were downregulated in MP compared to PP, including categories related to the cell cycle (GO:0000786), ATP hydrolysis (GO:0015991), and protein heterodimerization (GO:0051258).

10.1128/mBio.00029-17.3 DATA SET S2  Enrichment of GO categories in pairwise comparisons between the different developmental stages. Download DATA SET S2, PDF file, 0.1 MB. Copyright © 2017 Inbar et al. 2017 Inbar et al. This content is distributed under the terms of the Creative Commons Attribution 4.0 International license.

**FIG 3:**
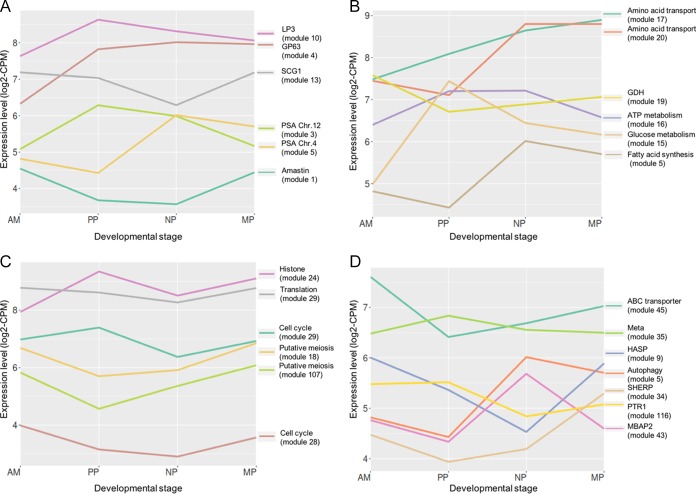
Selected coexpression modules showing dynamics of cellular processes across *L. major* developmental stages in the sand fly. Each line represents a plot of the median expression level of a selected gene or group of genes in coexpression modules relating to cell surface (A), metabolism (B), cell cycle and gene expression (C), and differentiation, stress, and autophagy (D). Values shown are the log_2_ counts per million and represent the proportion of reads mapped to each *Leishmania* gene, multiplied by 10^6^.

We also generated coexpression gene modules for mRNAs with similar patterns of abundance across the different developmental stages (see Data Set S4 for the list of modules and corresponding genes). Our analysis resulted in 207 gene modules with a median number of 31 genes per module (minimum of 10, maximum of 295). All of the 207 coexpression modules are presented in Data Set S3. In addition, coexpression gene modules were enriched by using GO and KEGG tools (see Data Set S5 for the list of GO and KEGG categories in the modules). In the sections below, based on the top up- and downregulated mRNAs in each of the stages (Data Set S1), the most dynamic cellular processes are described. To highlight interesting coexpression modules, we generated median expression plots for modules enriched for specific functions of interest described below (Fig. 3). Each plot shown was selected because it included the greatest number of genes annotated with a particular structure or function. For example, while amastin genes are represented in a number of different coexpression modules, module 1 was chosen because it contains the largest number of amastin genes. The specific genes that are mentioned throughout our manuscript were significantly different (*P <* 0.05) between at least two stages (see Data Set S1).

10.1128/mBio.00029-17.4 DATA SET S3  Coexpression modules for genes with similar mRNA abundance patterns across the different developmental stages. There were a total of 207 coexpression modules. Each line represents a gene, with genes of particular interest labeled with different colors and/or line patterns. The counts per million data represent the proportion of reads mapped to each *Leishmania* gene, multiplied by 10^6^. Download DATA SET S3, PDF file, 0.5 MB. Copyright © 2017 Inbar et al. 2017 Inbar et al. This content is distributed under the terms of the Creative Commons Attribution 4.0 International license.

10.1128/mBio.00029-17.5 DATA SET S4  List of modules and corresponding genes. Download DATA SET S4, XLSX file, 0.6 MB. Copyright © 2017 Inbar et al. 2017 Inbar et al. This content is distributed under the terms of the Creative Commons Attribution 4.0 International license.

10.1128/mBio.00029-17.6 DATA SET S5  List of GO and KEGG categories in the modules. Download DATA SET S5, PDF file, 0.2 MB. Copyright © 2017 Inbar et al. 2017 Inbar et al. This content is distributed under the terms of the Creative Commons Attribution 4.0 International license.

### Surface genes.

Strong stage regulation was observed in surface proteins between the different developmental forms. Ten out of the 15 most downregulated transcripts as the parasites differentiate into PP, 2 days PBM, were amastin, the amastigotes surface proteins (Data Set S1, PP versus AM). We found 30 significantly regulated amastin-like mRNAs, most of these transcripts affiliated with modules that show a strong downregulation in PP and an increase in NP or MP (Fig. 3A; Data Set S3, modules 1, 2 and 28). Many amastin-like mRNAs returned to their AM expression levels already at the MP stage. The most strongly upregulated mRNAs in PP were the promastigote surface antigen proteins (PSA) on chromosome 12 (Data Set S1, PP versus AM). The majority of the PSA transcripts (19 out of 22) showed higher expression in PP compared to the other promastigote forms, with 5 of these genes showing a sharp peak of abundance in PP (Fig. 3A; Data Set S3, module 3). The coordinate upregulation of these genes in PP may suggest their role in the resistance of the transforming extracellular promastigotes to the proteolytic enzymes that are induced by blood feeding and that reach their peak 18 to 48 h post-blood meal (24). Transcripts for other surface proteins, such as those encoded by adjacent genes on chromosome 4 and a single gene on chromosome 21, increased significantly at the NP stage and remained elevated in MP (Fig. 3A, module 5, and Data Set S1, NP versus PP). Another surface antigen-like protein on chromosome 5 was upregulated only in NP versus the AM stage (Data Set S3, module 6).

The *Leishmania* surface proteinase of 63 kDa (GP63, or leishmanolysin) is encoded by 7 genes in *L. major* and is thought to be a virulence factor involved in complement resistance and also interaction with and survival in macrophages. mRNAs of gp63-1, -2 and -4 on chromosome 10 (LmjF.10.0460, LmjF.10.0465, and LmjF.10.0480) were significantly upregulated in PP and continued to increase at the later stages (Fig. 3A, module 4). In contrast, GP63 on chromosome 28 (LmjF.28.0570) showed a sharp peak at the MP stage (Data Set S3, module 7). The abundant surface lipophosphoglycan (LPG) is another virulence factor for promastigote survival in both the sand fly and mammalian host. LPG3 is essential for the parasite survival due to its role in the biosynthesis and assembly of glycophospholipid (GPI)-anchored glycoconjugates, including GP63 and LPG (25). LPG3 transcripts were strongly upregulated in PP but significantly decreased in MP (Fig. 3A, module 10), consistent with its upregulated expression in log-phase versus stationary-phase culture promastigotes (25). Surprisingly, other LPG biosynthetic genes did not show significant differences between the stages, apart from the galactosyltransferases that modify the phosphoglycan side chains of the LPG (SCGs), for which SCG1 was downregulated in PP and NP versus AM while SCG7 was specific to AM and strongly downregulated in all other stages (Fig. 3A; see also Data Set S3, modules 12 and 13). Two different mRNAs encoding SCG5 showed different patterns of abundance. LmjF.31.3090 was upregulated in PP and NP, and LmjF.31.3190 was downregulated in NP and MP compared to AM (Data Set S3, modules 11 and 154). SCAL (LmjF.34.0510), the arabinosyltransferase that modifies the LPG side chain oligosaccharides with arabinose, is abundant in amastigotes but remains significantly downregulated at the NP and MP stages (Data Set S3, module 183). This was surprising, as it is known that LPG side chains are arabinosylated on MP and are thought to facilitate the detachment of the parasite from the midgut epithelium (26).

### Metabolism.

*Leishmania* spp. utilize different carbon sources for the production of energy throughout their life cycle. In the insect, extracellular promastigotes utilize mainly sugar and amino acids, while intracellular amastigotes use amino acids and fatty acids (27–31). Combining coexpression module analysis with GO and KEGG pathway enrichment analyses, our studies confirmed that transport of different sugars and amino acids was higher in PP than AM, further increased from PP to NP, and reached their peak at MP (Fig. 3B; see also Data Sets S5, module 17, and S2, E and G). This pattern was shared between the glucose transporters GT1, GT2, and GT3 (LmjF.36.6300, LmjF.36.6290, and LmjF.36.6280, respectively) and the amino acid transporter AAP24 (LmjF.10.0720, LmjF.10.0715, and LmjF.10.0720, also known as AAT20 1 to 3). This transporter is specific for “nonessential” amino acids, such as proline and alanine, that are known to serve as carbon sources in *Leishmania* (4, 30–33). Moreover, the transporter has a role in maintaining the homeostasis of amino acids in the free amino acid pool, in which proline, alanine, and glutamate are the most abundant constituents (32, 34). The significant increase in acquisition of carbon sources throughout the development of promastigotes in the fly is consistent with recent studies showing that the differentiation of *L. mexicana* to amastigotes is coupled with them entering into a “metabolic stringent state,” in which transport and utilization of glucose and amino acids are strongly suppressed, while metabolism of fatty acids via β-oxidation and production of glutamate via the tricarboxylic acid (TCA) cycle is increased (4). These observations suggest that promastigotes build a reservoir of carbon sources that can be utilized in the nutrient-poor intracellular environment.

In contrast to glucose transport, mRNAs for glucose metabolism showed a different pattern of abundance. Three out of four glyceraldehyde-3-phosphate dehydrogenase (GAPDH) variants were significantly more abundant in all promastigote stages versus AM, with two of them reaching their peak already at PP (LmjF.30.2980 and LmjF.35.4750) (Fig. 3B; see also Data Set S5, module 15) and a third (LmjF.30.2970) peaking at the NP stage. Conversely, the putative cytosolic GAPDH, LmjF.36.2350, was upregulated in AM and MP and significantly downregulated in PP and NP (Data Set S3, module 18). Other glycolysis enzymes, such as the phosphoglucose isomerase (PGI) (LmjF.12.0530) and the putative hexokinase (LmjF.21.0240), were significantly upregulated in PP and NP compared to AM, with no significant difference between MP and AM (modules 15 and 145, respectively), suggesting that glycolysis is downregulated at the MP stage, possibly as a preadaptation to the intraphagosomal environment, where energy is produced by other carbon sources, such as fatty acids and amino acids. A main pathway of amino acid utilization in *Leishmania* is through the metabolism of glutamate (32, 33). Glutamate is metabolized by glutamate dehydrogenase (GDH), which converts glutamate to α-ketoglutarate to enter the TCA cycle and vice versa. Two mRNAs for GDH were significantly regulated in this study: LmjF.15.1010 was upregulated by 2.79-fold in PP compared to AM, remained low in NP, and was downregulated again in MP by 2.38-fold (Data Set S1). Another GDH (LmjF.28.2910) was strongly downregulated between AM and PP (6.29-fold), slightly increased in NP, and returned to its high AM abundance level at the MP stage (Fig. 3B; Data Set S3, module 19). Saunders et al. in 2014 reported that metabolism of glutamate takes place in intracellular amastigotes via acetyl coenzyme A (CoA) in a compartmentalized and highly active TCA cycle (4). The strong upregulation of LmjF.28.2910 in MP to its high abundance level in AM suggests that this process already arises in the MP stage.

While mitochondrial activity is suggested to increase in MP and AM, general metabolism of ATP seemed to be more pronounced in NP and PP. GO categories that are related to ATP metabolism (GO:0000275, GO:0015991, and GO:0046034) were upregulated in PP and NP but were decreased in AM and MP (Fig. 3B; Data Sets S5, module 16, and S2, B and F).

Although in *Leishmania* the use of fatty acids as a carbon source is predominantly associated with the intracellular amastigote stage, it was also evident in nondividing promastigotes in culture (27, 35). We observed that the biosynthesis of fatty acids as well as the catabolism of ketone bodies were strongly upregulated in NP. The mRNAs of two fatty acid elongase enzymes (LmjF.14.0700 and LmjF.14.0720) were significantly upregulated in NP compared to AM and PP, while a third, LmjF.14.0730, showed a significant peak in NP compared to all other forms (Fig. 3B; Data Set S3, modules 5 and 43). Likewise, two putative fatty acid desaturases (LmjF.36.6950 and LmjF.24.2250) and one putative acetyl-CoA carboxylase (LmjF.31.2970) were affiliated with modules 6, 92, and 43, respectively (Data Set S3), all of which are characterized by a significant peak at the NP stage. In addition, we observed a strong increase in the mRNAs of succinyl-CoA:3-ketoacid (LmjF.30.1930, LmjF.30.1940, and LmjF.33.2340) in the NP stage that remained elevated in MP (Data Sets S3, modules 17, 20, and 22, and S2, G and I; GO:0046952). This enzyme participates in the catabolism of ketone bodies by converting acetoacetate to acetoacetyl-CoA under low-nutrient conditions.

Additional evidence that NP are experiencing starvation conditions was found in the abundance patterns of specific amino acid transporters. While mRNAs of many amino acid transporters accumulated throughout promastigote development, with a peak of abundance in MP (module 17), others, including some members of the aATP11 family and the arginine transporter AAP3 (LmjF.31.0870 and LmjF.31.0880), showed a sharp increase in the NP stage (Fig. 3B, modules 20 and 126; Data Set S1, NP versus PP). aATP11 and AAP3 were previously associated with a response to purine and amino acid starvations, respectively (36–38), reinforcing the suggestion that the NP stage is associated with one or a number of stress conditions.

### Cell cycle and gene expression.

Histone proteins were among the most strongly upregulated transcripts during differentiation from AM to PP (Data Set S1, PP versus AM). GO enrichment along with coexpression clustering analysis indicated that most mRNAs of histone proteins (categories GO:0000786 and GO:0006334, nucleosome and nucleosome assembly) were affiliated with modules 8, 23, and 24 (Fig. 3c; Data Set S3 and S5). The shared expression pattern in these modules is a significant increase in PP followed by a decrease in NP (Data Set S2, B and C) and a more modest increase at the MP stage. The tight association between histone protein abundance and cell cycle in *Leishmania* has been previously described (39). The mRNAs of genes that are related to cell replication showed a similar pattern to histones: strongly upregulated in PP and downregulated in NP, followed by a modest upregulation in MP. These include cyclin6 (LmjF.32.3320), CDC20 (LmjF.24.1720), cell cycle sequence binding phosphoprotein (RBP33) (LmjF.35.0950), and cyclin-dependent kinase regulatory subunit (LmjF.32.3790) (Fig. 3C; Data Set S3, modules 25, 26, 28, and 163). CyclinA (LmjF.25.1470) was an exception, as it was elevated in AM and PP but significantly decreased in NP and MP (Data Set S3, module 27). Consistently, all of the mRNAs for DNA polymerases were downregulated in the nectomonad stage, including the mitochondrial DNA polymerase beta (LmjF.08.0890) and the mitochondrial DNA polymerase I protein C (LmjF.14.0920) (Fig. 3C; Data Set S3, modules 28 and 163).

Together, the results suggest cell cycle arrest in NP, which is consistent with the absence of dividing nectomonads observed by light microscopic examination of stained cells (21). Surprisingly, there was an increase in most cell cycle-related mRNAs in the metacyclic stage. Gossage et al. (21) identified two separate growth phases during promastigote development *in vivo*, the procyclic and leptomonad forms, that appeared prior to or coincident with the appearance of MP. The increase of cell cycle-related mRNAs in MP may be due to incomplete degradation of mRNAs from a previous, cycling promastigote form (i.e., leptomonad) that was not isolated in this work. Another possible explanation for the increase in cell cycle mRNAs in MP is the occurrence of meiosis, which shares both DNA synthesis and DNA-packaging processes with mitosis. Sexual mating of *Leishmania* occurs in the sand fly vector (40, 41), and while the mating competent form(s) is yet to be directly identified, the recovery of hybrid progeny was greatest during the later stage of infection (days 10 to 14) (41). Meiosis homologue genes were previously found to be active in *Trypanosoma brucei* in the salivary glands of the tsetse fly (42). Three orthologs of these potential meiosis-specific genes (SPO11 [LmjF.16.0630], HOP1 [LmjF.36.1110], and DMC1 [LmjF.35.4890]) were found to be significantly downregulated in PP and most abundant in MP (Fig. 3C, modules 18 and 107; Data Set S3, module 199).

GO and KEGG enrichment analyses revealed that the majority of ribosomal constituents and other translation-associated genes (GO:0005840, GO:0006412, GO:0003735, GO:0006412, and MD:03010) are downregulated at the NP stage (Fig. 3C, module 29; Data Sets S3 and S5, modules, 32, 69, 113, 120, 148, 150, and 207). Conversely, a small number of translation-related mRNAs reached their peak of abundance at NP (Data Sets S3 and S5, modules 98 and 113). Generally, the results suggest that translation decreases as the parasites differentiate into PP, remains low at the NP stage, and begins to increase again in the MP stage, as a possible preadaptation to the intracellular conditions.

### Differentiation, autophagy, and the stress response.

The loci on chromosome 23 that contain the hydrophilic acylated surface proteins (HASP) (LmjF.23.1040, LmjF.23.1060, LmjF.23.1070, LmjF.23.1082, and LmjF.23.1088) and the small hydrophilic endoplasmic reticulum-associated protein (SHERP) (LmjF.23.1050, LmjF.23.1080, and LmjF.23.1086) were previously shown to be important in metacyclogenesis and in macrophage invasion by MP (43, 44). The HASP abundance pattern was consistent with these findings, as it was specific to AM and MP, downregulated in PP, and reached its negative peak in NP (Fig. 3D, module 9; Data Set S3, module 144). SHERP mRNAs were among the most abundant in the MP stage, but unlike HASP, they did not remain upregulated in AM (Fig. 3D, module 34; Data Set S1, MP versus AM, MP versus PP, and MP versus NP), suggesting that following the initial establishment of infection, only HASP continues to play a role in the infectious process in the mammalian host.

Biopterin metabolism provides cofactors for lipid cleavage, hydroxylation of aromatic amino acids and synthesis of nitric oxide (45–48). Previous studies showed that in *Leishmania*, both folate and biopterin can be metabolized via the same enzyme, PTR1 (49). Strikingly, it was shown that mutants lacking PTR1, and thus deficient in tetrahydropterin (H_4_B), generated significantly more metacyclics in culture and were more virulent (50). Moreover, PTR1 mRNA and protein were shown to be stage regulated and decreased dramatically in stationary versus log phase promastigotes (51, 52), reinforcing the role of pteridine metabolism in metacyclogenesis. In the present study, two folate/biopterin transporters (LmjF.10.0380 and LmjF.06.0310) and one pteridine transporter (LmjF.06.1260) were significantly upregulated in NP and MP (Data Set S3, modules 5, 37, and 122; Data Set S1, NP versus PP). A third folate/biopterin transporter (LmjF.10.0390) was AM specific, with low abundance in all promastigotes (Data Set S3, module 2). While acquisition of biopterin and pteridine appeared strongly upregulated in NP and MP, the mRNA abundance for PTR1 (LmjF.23.0270) which reduces pteridine, was strongly downregulated in NP and only slightly increased in MP (Fig. 3D; Data Set S3, module 116), suggesting that shortage of tetrahydropterin in NP may contribute to the induction of metacyclogenesis, consistent with what was previously reported (50).

Recycling of proteins by autophagic mechanisms is associated with the metabolism in cells that are under stress conditions and/or undergoing a differentiation process (53, 54). In *L. major*, autophagy was shown to be essential for metacyclogenesis and was significantly upregulated in culture metacyclics (55, 56). It was shown in a later study that recycling of the glycosome compartment via autophagy is an important aspect of this response (57). ATG8 is involved in completion and expansion of the autophagosome vesicles and was previously used as a marker for autophagy in *Leishmania* (56–58). In our study, putative ATG8 mRNAs on chromosomes 9 and 19 were among the most strongly upregulated in the NP stage (Fig. 3D; Data Sets S3, modules 5 and 126, and S1, NP versus PP). A ubiquitin-dependent, proteasome-related protein (LmjF.14.0310) was also specific to NP and significantly decreased in MP (Data Set S3, module 39).

Another factor that may trigger protein recycling in *Leishmania* is purine starvation. *Leishmania* parasites do not synthesize purines *de novo* and must scavenge them from the environment (59). In response to purine starvation, the parasites undergo major metabolic and cellular changes, including cell elongation, cell cycle arrest, and upregulation of the nucleotide transport and purine salvage machinery (36, 60). Changes in the transcriptome of *L. donovani* following purine starvation have been previously reported (36), and are reflected in the current studies. For example, one of the most upregulated genes in purine starved parasites was the membrane-bound acid phosphatase (MBAP2) which has a role in endosomal trafficking (61). The mRNA for one MBAP2, LmjF.28.2650, was among the most strongly upregulated in NP compared to all other stages. A second, LmjF.23.1170, was significantly upregulated only in NP versus AM (Fig. 3D, module 43; Data Sets S3, module 41, and S1, NP versus PP), suggesting an increase in lysosome-related recycling processes in NP. Other similarities include the increase in amino acid transporters aATP11 and in autophagy-related genes in the NP and MP stages, as discussed in the previous sections. Together, these observations suggest that promastigotes differentiation to NP and to MP involves a response to one or a number of stress conditions (e.g., purine shortage), which trigger protein recycling via autophagy or proteasome pathways. The initiation and in some cases the confinement of the peak of these responses to NP indicate that these stress conditions may vary according to the time and place within the post-blood meal midgut anatomy.

### Metacyclogenesis: sand fly versus culture.

We compared the change in transcriptome between sand fly-derived PP and MP to the difference recently described between log-phase and metacyclic promastigotes in culture (7) (Tables 1 and 2). Six of the top 10 upregulated mRNAs observed in culture with metacyclics versus log-phase promastigotes were not significantly upregulated when sand fly-derived MP and PP were compared (Table 1). This included the Meta1 gene (LmjF.17.0890), previously associated with metacyclogenesis in culture (62), which was upregulated in all promastigote forms *in vivo* with a small peak in NP, indicating that this gene is not directly related to metacyclogenesis. Four genes were consistent between the two differentiation conditions, including the RNA-binding protein (LmjF.23.0730) and the phosphoinositide phosphatase (LmjF.22.0250). Only 3 out of the 10 genes that were strongly downregulated in culture metacyclics were also downregulated in sand fly-derived MP: two histone H4s (LmjF.36.0020 and LmjF.31.3180) and the ATPase subunit 9 (LmjF.21.0740). Most of the top 10 mRNAs that were upregulated in sand fly-derived MP versus PP were also significantly upregulated in culture metacyclics versus log-phase promastigotes, although to a lesser extent (Table 2). This included the three SHERP mRNAs (LmjF.23.1050, LmjF.23.1080, and LmjF.23.1086) and an aATP11 transporter (LmjF.31.0350). The ABCA6 transporter (LmjF.11.1290), which was significantly increased in MP versus PP *in vivo*, was downregulated in metacyclic versus log-phase promastigotes from culture. Importantly, whereas 6 out of the 10 most downregulated mRNAs in MP versus PP were the promastigote surface proteins (PSAs) on chromosome 12, these genes were not downregulated in cultured metacyclics, and in some cases they were slightly increased. Thirty-three GO categories were found to be significantly downregulated in metacyclics versus log-phase promastigotes in culture (see Table 2 in Dillon et al. [7]). In contrast, only 8 GO categories were downregulated in MP versus PP *in vivo*, including those related to nucleosomes and ATP metabolism (Data Set S2). Seven GO categories were found to be upregulated in metacyclics versus log-phase cells in culture, and most were related to signal transduction and protein phosphorylation (7). In contrast, 14 GO categories were shown to be significantly upregulated in MP versus PP *in vivo* (Data Set S2, E), and the majority of these were related to nutrient uptake, while two were related to phosphorylation of nucleotides and signal transduction. Together, these discrepancies reflect unique factors influencing promastigote survival and differentiation *in vivo*, including the diet, physiology, and microbiota of the fly. Thus, the abundance of PSAs expressed by PP in the fly but not by log-phase promastigotes from culture suggests a role in protecting the parasites in the hydrolytic environment of the blood-fed midgut (63). In addition, the upregulation in mRNAs for amino acid transporters in sand fly-derived MP but not metacyclics from culture may reflect a response to a shortage in these amino acids in flies transitioning from blood to sugar meals, or to competition for nutrients by the gut microflora.

**TABLE 1:** The top 10 up- or downregulated mRNAs between culture MP and log-phase promastigotes compared to the difference between sand fly MP and PP

Direction of regulation and gene ID	Product description	Fold change	
		Culture MP vs log-phase promastigotes (Dillon et al.)	Sand fly MP vs PP (this work)
Upregulated in culture metacyclics			
LmjF.34.0070	Ascorbate peroxidase (APX)	3.61	NS^a^
LmjF.17.0890	META domain-containing protein (META1)	3.07	NS
LmjF.02.0460	Voltage-dependent anion-selective channel putative	3.03	NS
LmjF.23.0730	RNA-binding protein putative	2.78	4.22
LmjF.12.0480	Hypothetical protein unknown function	2.73	6.88
LmjF.16.0500	Hypothetical protein unknown function	2.71	NS
LmjF.28.0980	P27 protein putative	2.69	NS
LmjF.23.0780	Hypothetical protein conserved	2.68	8.46
LmjF.22.0250	Phosphoinositide phosphatase	2.63	5.92
LmjF.29.1350	RNA-binding protein putative	2.59	NS
Downregulated in culture metacyclics			
LmjF.31.3070	Iron-zinc transporter protein-like protein (LIT1)	−3.13	NS
LmjF.33.1760	Hypothetical protein unknown function	−2.91	NS
LmjF.35.1310	Histone H4	−2.89	NS
LmjF.35.2130	Hypothetical protein unknown function	−2.82	NS
LmjF.36.0020	Histone H4	−2.72	−2.08
LmjF.35.2160	Adenine aminohydrolase (AAH)	−2.72	7.04
LmjF.14.0470	Hypothetical protein conserved	−2.69	NS
LmjF.31.3180	Histone H4	−2.66	−2
LmjF.33.3240	h1 histone-like protein	−2.58	NS
LmjF.21.0740	ATPase subunit 9 putative	−2.58	−2.08

a NS, not significant; differences between growth stages were not statistically significant.

**TABLE 2:** The top 10 up- and downregulated mRNAs between sand fly MP and PP compared to the change between MP and log-phase promastigotes from culture

Direction of regulation and gene ID	Product description	Fold change	
		Culture MP vs log-phase promastigotes (Dillon et al.)	Sand fly MP vs PP (this work)
Upregulated in MP			
LmjF.23.1084	Hypothetical protein	1.98	32.93
LmjF.23.1086	Small hydrophilic endoplasmic reticulum-associated protein (SHERP2)	1.90	31.55
LmjF.23.1075	Hypothetical protein	1.93	29.00
LmjF.23.1050	Small hydrophilic endoplasmic reticulum-associated protein (SHERP)	1.88	28.80
LmjF.23.1080	Small hydrophilic endoplasmic reticulum-associated protein (SHERP1)	1.88	25.64
LmjF.33.1290	Hypothetical protein conserved	1.72	24.29
LmjF.31.0350	Amino acid transporter aATP11 putative (AAT1.4)	1.29	24.12
LmjF.13.0190	Hypothetical protein unknown function	1.68	19.81
LmjF.26.0160	Nuclear *lim* interactor-interacting factor-like protein	2.18	10.42
LmjF.11.1290	ATP-binding cassette protein subfamily A member 6 putative	−1.34	10.30
Downregulated in MP			
LmjF.12.1040	Surface antigen protein putative	1.36	−16.60
LmjF.12.1060	Surface antigen protein putative	1.31	−14.20
LmjF.12.1020	Surface antigen protein putative	1.36	−11.77
LmjF.12.0910	Promastigote surface antigen protein	1.32	−10.79
LmjF.12.0860	Surface antigen protein putative	1.35	−9.75
LmjF.12.0920	Promastigote surface antigen protein	1.35	−9.15
LmjF.14.0130	Inosine-guanine nucleoside hydrolase putative	1.31	−7.74
LmjF.15.1090	Developmentally regulated protein putative	1.09	−7.25
LmjF.06.0210	Hypothetical protein conserved	−1.51	−5.94
LmjF.13.0870	Mitochondrial processing peptidase alpha-subunit putative	−1.24	−5.86

While the shifting transcriptional programs comparing metacyclics and propagating promastigotes displayed important differences depended on their *in vitro* or *in vivo* origins, a comparison between the metacyclics themselves revealed a remarkably similar profile of transcript abundance, with only 26 DEGs distinguishing the *in vitro* and *in vivo* forms (Data Set S1, SFMP versus CMP). Such a high level of concordance was not observed in a recent microarray-based transcriptome comparison of *L. infantum* promastigotes from stationary-phase culture and from the anterior midgut of *P. perniciosus* (8), for which over 260 DEGs were reported. We suggest that, whereas relatively homogeneous populations of metacyclics from sand flies (>90%) and culture (>95%) were compared in the present studies, the *L. infantum* promastigote stages compared previously were not identified and likely contained heterogeneous populations of promastigotes. Moreover, compared to RNA microarrays, RNA-Seq requires less input RNA without the need for an artificial amplification step or the avoidance of sand fly tissue. Among the consistencies found, the pteridine transporters LmjF.06.1260 and its ortholog in *L. infantum*, LinJ.06.1320, were in each case significantly higher in sand fly metacyclics than in culture-derived forms. Pteridine transport and metabolism are crucial for *Leishmania* differentiation and virulence (51, 52).

In the current study, the most highly upregulated gene in sand fly MP compared to culture was LmjF.14.0440, which according to GO prediction encodes the enzyme flavin adenine dinucleotide-binding oxidoreductase, which participates in energy production via an oxidation-reduction process. The nucleoside transporter 1 (LmjF.15.1230) and the glucose transporters 1 and 2 (LmjF.36.6290 and LmjF.36.6300, respectively) were also increased in sand fly versus culture metacyclics, as was MBAP (LmjF.36.2590), which was previously associated with the response to purine starvation (36, 60, 61). All together, the transcriptional programs associated with metacyclogenesis in the anterior midgut of the fly and in stationary-phase culture show exceptional consistency, with *in vivo* forms likely driven to a more fully mature state by exposure to different and/or more severe stress conditions. These conditions may include the hyperosmolality associated with the high concentration of sugar diffusing from the crop into the anterior midgut and the exceptionally high density of promastigotes that accumulate behind the stomodeal valve, which along with the microbiota will compete for nutrients in the anterior gut.

### Conclusions.

This work presents the most thorough examination to date of the transcriptomes of *Leishmania* associated with the life cycle progression in the sand fly midgut from tissue amastigotes ingested with the blood meal to fully mature, infective-stage metacyclic promastigotes that are transmitted by bite back to the mammalian host. The unique transcriptomic profile of each of the different stages likely reflects their adaptation to distinct microenvironments in the vector. The transformation of AM to rapidly dividing extracellular PP in the blood-fed midgut was accompanied by the greatest degree of genetic reprogramming, with over 1,200 DEGs. A substantial part of this shift was devoted to altering the expression of genes encoding surface proteins, with the downregulation of amastins observed in parallel to a strong increase in promastigotes surface antigens located on chromosome 12. It is likely that these surface changes reflect at least in part the need for the extracellular parasite to protect itself from the digestive enzymes induced by blood feeding (63).

Our studies are the first to provide information about the transcriptome of NP, a distinctive developmental form that has so far not been studied, and only rarely even identified, in populations of promastigotes obtained from culture. NP differentiate from PP and dominate the gut immediately following excretion of a digested blood meal. Their differentiation was associated with a strong reduction in cell cycle-related and ribosomal protein mRNAs, and by a strong upregulation of responses associated with nutrient stress, including amino acid transport, fatty acid biosynthesis, catabolism of ketone bodies, and protein recycling via autophagy. Some of these DEGs, like those resembling the response to purine starvation, were confined to NP and were not apparent in MP. Despite both stages arising in a post-blood meal nutritional environment and both receiving cues to enter cell cycle arrest, their distinctive morphologies and gene expression profiles nonetheless argue for their exposures to distinctive nutrient and stress conditions. Sugar meals are stored in the crop and passively diffuse into the thoracic midgut (18), such that the concentration of sugars is apt to be greater for promastigotes that have migrated anteriorly. Indeed, the availability of nutrients in the anterior gut is adequate for some NP to reenter the cell cycle as leptomonads prior to their differentiation to MP and their return to a resting state (21). The earlier differentiation of PP to nonreplicating NP that occurs in the posterior midgut following excretion of the blood meal remnants may be in response to more extreme starvation conditions.

Extensive transcriptomic differences were found between the MP and the earlier promastigote stages *in vivo* that in most cases did not reflect the DEG in the comparison of log-phase and metacyclic promastigotes from culture (7). Since the transcriptomes of metacyclics obtained from flies and culture were themselves highly similar, the fact that the changes in gene expression that accompanied metacyclogenesis *in vivo* and *in vitro* were in such poor agreement suggests that the transcriptomes of the respective proliferative forms (PP and log-phase promastigotes) are apt to be highly dissimilar. Interestingly, Pearson correlation and principal component analysis suggested a greater similarity between MP and AM than to the other promastigote stages that would indicate a preadaptation process of the metacyclics to the mammalian host environment. For instance, many of the mRNAs that encode amastin, the main surface glycoprotein that is essential for amastigote intracellular survival (64), are strongly upregulated already in the MP stage. From the metabolic point of view, increased mitochondrial activity that is associated with amastigote survival and growth already occurs at the MP stage.

Metacyclic promastigotes obtained from culture rather than from flies are overwhelmingly relied upon for experimental studies, and the conditions and methods that promote their *in vitro* differentiation and purification have been well-described (25, 65). It seems generally encouraging then that the transcriptomic profiles of the sand fly- and culture-derived metacyclics were highly similar, with only 26 mRNAs significantly differing in abundance. This is consistent with the generally high level of infectivity that culture-derived metacyclics display. For example, injection of as few as 100 purified, culture-derived *L. major* metacyclics was able to reproducibly establish infection in the ear dermis of C57BL/6 mice (66). Nonetheless, the few genes that differ in abundance between these populations suggest that sand fly metacyclics are a more fully differentiated, infective stage and that direct infectivity comparisons using even lower doses or other mammalian hosts might reveal meaningful differences.

## MATERIALS AND METHODS

### Sand flies and parasites.

A laboratory-reared colony of *Phlebotomus duboscqi*, a natural vector for *L. major*, was used in this study. The colony was initiated from field specimens collected in Mali. The *L. major* Ryan strain used in this study was derived from a strain originally isolated from a lesion biopsy specimen of a laboratory worker accidentally exposed to sand flies that were experimentally infected with a strain of *L. major* (WR2885) originating in Iraq (67). Promastigotes were grown at 26°C in complete medium 199 (CM199) supplemented with 20% heat-inactivated fetal calf serum (FCS), 100 U/ml penicillin, 100 µg/ml streptomycin, 2 mM l-glutamine, 40 mM HEPES, 0.1 mM adenine (in 50 mM HEPES), 5 mg/ml hemin (in 50% triethanolamine), and 1 mg/ml 6-biotin. Lesion amasigotes were obtained from BALB/c footpads that were initiated using metacyclic promastigotes, that were purified by centrifugation through Ficoll as described elsewhere (68). Mice were maintained in the National Institute of Allergy and Infectious Diseases (NIAID) animal care facility under specific-pathogen-free conditions and used under a study protocol approved by the NIAID Animal Care and Use Committee (protocol LPD 68E).

### Sand fly infection and parasite recovery.

Amastigotes were recovered from the mouse footpads, and ~3 × 10^7^ parasites were used for tissue amastigote RNA. A small portion of the amastigotes (~1 × 10^6^) was seeded in CM199 and metacyclics were purified from 6-day stationary cultures by using a Ficoll gradient. Four million footpad-derived amastigotes were mixed with 1 ml mouse blood for sand fly infection. Flies were fed through a chick skin membrane as described elsewhere (41). Midguts were dissected 2, 4, and 15 days PBM to obtain homogeneous populations of procyclic, nectomonad, and metacyclic promastigotes, respectively. Parasites were released by macerating midguts individually with a pestle (Kimble Chase, Vineland, NJ) in an Eppendorf tube containing 100 µl phosphate-buffered saline, and promastigote stages and numbers were determined by counting with a hemocytometer. A representative sample of DNA from each stage was stained using Hoechst fluorescent dye and observed under a Leica fluorescence microscope at 100× magnification. Only midguts yielding the respective promastigote stages with >90% homogeneity were pooled for RNA. The samples were homogenized in 1 ml of Trizol reagent (Tri reagent; Molecular Research Center Inc.) and stored at −80°C until further processing. Two biological replicates originating from different footpad lesions or groups of infected flies were prepared for each developmental stage.

### RNA extractions.

RNA was extracted from all samples by using a combination of two kits as follows: 0.2 ml of chloroform was added to the samples in Trizol and vigorously vortexed. Samples were incubated for 5 min at room temperature followed by 15 min of centrifugation at 15,000 × *g*, 4°C. The upper aqueous phase was carefully collected and mixed with 70% ethanol–diethyl pyrocarbonate. The mix of RNA and ethanol was added to an RNeasy column (catalog number 74106; Qiagen), and RNA was purified according to the manufacturer’s instructions, including a DNA digestion step. To confirm the lack of DNA in the samples, cDNA was produced with and without reverse transcriptase enzyme and used as the template for PCR amplification of G6PDH on *Leishmania* chromosome 34, as described in reference 41. Amplifications were observed only on cDNAs produced with the reverse transcriptase.

### RNA-Seq*.*

Due to low RNA yields, the SMARTer Ultra Low Input RNA kit for sequencing (v. 3; Clontech Laboratories, Inc., Mountain View, CA) was used for cDNA synthesis with a template input amount of 5 ng. The Illumina TruSeq Stranded mRNA-Seq HT sample preparation kit (Illumina, San Diego, CA) and its workflow were used for preparation of dual-indexed transcriptome libraries. A normalized input amount of 20 ng total RNA was used for the library prep. Final library products were measured by quantitative PCR using the Kapa Illumina Library quantification kit (Kapa Biosystems, Boston, MA), pooled at equal molar amounts, and sequenced on two lanes of a HiSeq 2500 8-lane flow cell (Illumina) to produce paired 100-bp reads. Trimmomatic v. 0.32 (69) was used to remove any remaining Illumina adaptor sequence and low-quality bases from the reads, and FastQC v. 0.11.2 (70) was used to assess the quality of reads before and after trimming.

### Read mapping and quantification.

TopHat version 2.0.13 (71) was used to map trimmed RNA-Seq reads to the TriTrypDB v. 6.0 *L. major* Friedlin reference genome (72). Mapped reads were then sorted with SAMtools v. 1.3 (73) and quantified using HTSeq v. 0.6.0 (74). For a complete description of the precise commands used for each of the above steps, refer to Text S1 in the supplemental material.

10.1128/mBio.00029-17.1 TEXT S1  Description of the precise commands used to generate the different steps in our study. Download TEXT S1, PDF file, 0.9 MB. Copyright © 2017 Inbar et al. 2017 Inbar et al. This content is distributed under the terms of the Creative Commons Attribution 4.0 International license.

Next, counts for each sample were loaded into R/Bioconductor (R Core Team, 2016 [75]) and combined into a single-count matrix. Unexpressed genes and genes with low levels of expression, defined as having less than 1 read per million in at least two of the samples, were removed. Quantile normalization (76) was applied, followed by log_2_ transformation of the counts per million and mean-variance adjustment using the Voom program (77).

### Sample quality assessment, differential expression, and GO analysis.

PCA and Pearson correlation-based heat maps created using the base R princomp and gplots heatmap.2 functions, respectively, were used to determine the similarity between samples and make sure no outliers or strong batch effects were present in the data. Samples clustered closely by condition with no evidence for a strong batch effect.

The generalized linear model (GLM)-based method of Limma (78) was used to detect differentially expressed genes for each pairwise contrast. *P* values were adjusted for multiple testing using the Benjamini-Hochberg (BH) method. Genes with an adjusted *P* value of <0.05 were considered differentially expressed. The goseq (79) package for R was used to detect over- or underrepresented GO terms among each set of differentially expressed genes, using the *L. major* Friedlin GO annotations provided by TriTrypDB. In order to improve the statistical power to detect enriched functions, up- and downregulated DEG were analyzed separately (80). The BH multiple-testing correction was applied, and GO terms with an adjusted *P* value of <0.05 were detected for each DEG contrast.

### Coexpression cluster analysis and enrichment analyses.

A modified version of the weighted gene coexpression network analysis (WGCNA) pipeline (81) was used to detect groups of *L. major* genes with similar expression patterns across the different developmental stages and growth conditions. Starting with the raw RNA-Seq counts for each sample, genes with low expression levels were removed, and read counts (counts per million) were log_2_ transformed, as described in the differential expression analysis section. For each pair of genes *i* and *j*, a similarity score *S*_*ij*_ was computed using a weighted combination of the Pearson correlation and Euclidean distance and was calculated as follows: *S* = SIGN (corr_*x*_) × {|corr_*x*_| + [1 − log(dist_*x*_ + 1)]/max[log(dist_*x*_ +1)]/2}, where corr_*x*_ is the correlation matrix and dist_*x*_ is the matrix used in calculation of the Euclidean distance. 

The Euclidean distance component of the similarity metric serves to penalize gene pairs with significantly different magnitudes of expression, thus helping to split up clusters with similar profiles but very different expression levels. The similarity matrix was then converted to an adjacency matrix by shifting the matrix to the range (0, 1) and raising the each similarity score to the power 4, helping to reduce the number of spurious correlations.

To detect coexpression clusters in the adjacency matrix, hierarchical clustering was applied to group genes by their similarity in expression, and a dynamic branch cut algorithm (82) was used to divide the resulting dendrogram into individual clusters. This resulted in a partitioning of the genes into 207 coexpression clusters (median size, 31 genes; minimum of 10, maximum of 295).

Each coexpression cluster was tested for enrichment of GO (83) and KEGG (84) annotations using the goseq package (79). Coexpression cluster analysis was performed in R/Bioconductor (R Core Team, 2016 [75]). All of the codes used for the coexpression cluster analysis, including commands used to generate all figures for this section, were documented using Knitr (85) and RMarkdown (86) (see Text S1).

### Accession number(s).

Raw sequence data are available at the NCBI Short Read Archive (SRA) under record SRP096578. Accession numbers for the individual samples are summarized in Table S1 in the supplemental material.
