# Supplementary material for: The Transcriptome of Leishmania major Developmental Stages in Their Natural Sand Fly Vector
Source: mBio. 2017 Apr 4;8(2):e00029-17. doi: 10.1128/mBio.00029-17 (PMC5380837; doi:10.1128/mBio.00029-17)
Supplement: TABLE S1 [file mbo002173254st1.docx]

Table S1. RNA sequence reads from the different samples

| **Replicate** | **Sample** | **# flies ^a^** | **# parasites (X10^6^) ^b^** | **RNA amount (ng)** | **# Reads (total)** | **# Reads (After trimming)** | **% Trimmed mapped to L. major ^c^** | **SRA accession numbers ^d^** |
| --- | --- | --- | --- | --- | --- | --- | --- | --- |
| A | AM | NA | 35 | 600 | 24,898,824 | 18,783,172 | 76.4 | SRX2485159 |
|  | PP | 20 | 0.64 | 150 | 24,065,534 | 17,667,850 | 21.2 | SRX2485186 |
|  | NP | 15 | 0.54 | 90 | 24,319,870 | 18,072,094 | 14.1 | SRX2485187 |
|  | MP | 22 | 1.2 | 60 | 25,554,028 | 18,827,286 | 14.1 | SRX2485188 |
|  | CMP | NA | 7 | 600 | 27,960,780 | 21,108,420 | 87.2 | SRX2485189 |
| B | AM | NA | 35 | 7800 | 28,590,448 | 21,707,810 | 76.9 | SRX2485190 |
|  | PP | 25 | 0.8 | 180 | 24,820,328 | 18,226,696 | 34.7 | SRX2485191 |
|  | NP | 15 | 0.45 | 60 | 25,556,180 | 19,329,884 | 14.6 | SRX2485192 |
|  | MP | 16 | 1.5 | 90 | 24,275,082 | 18,508,300 | 17.4 | SRX2485193 |
|  | CMP | NA | 5 | 1800 | 26,791,954 | 20,552,772 | 84.2 | SRX2485194 |

1. The number of midguts from flies with homogeneous parasite populations that were pooled together to generate the sample. (b) The number of parasites in the pool of midguts in each sample. (c) Reads were trimmed due to low quality. (d) Raw sequence data is available at the NCBI Short Read Archive (SRA) under records: SRP096578
