# Supplementary figures and images for: The Transcriptome of Leishmania major Developmental Stages in Their Natural Sand Fly Vector
Source: mBio. 2017 Apr 4;8(2):e00029-17. doi: 10.1128/mBio.00029-17 (PMC5380837; doi:10.1128/mBio.00029-17)

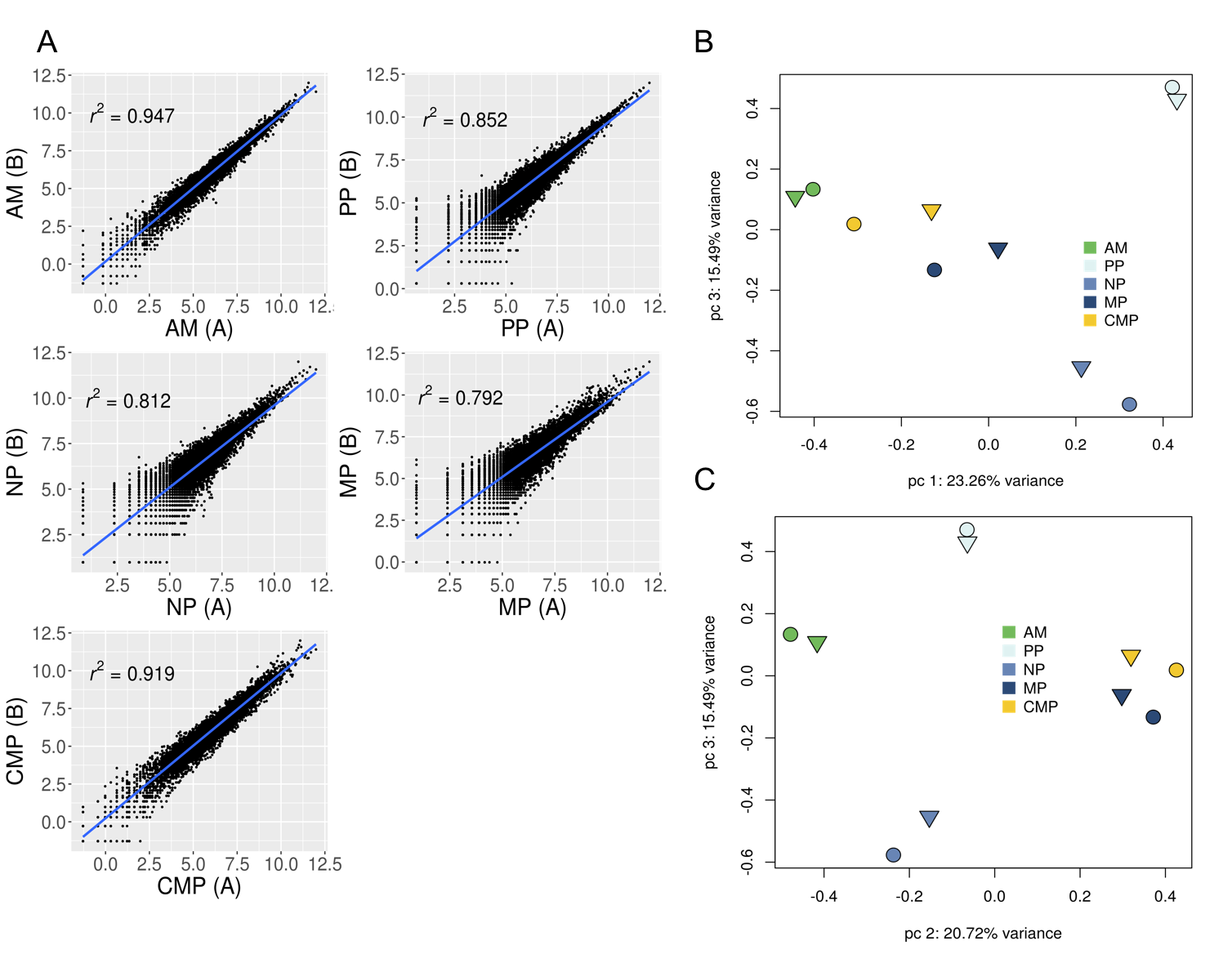

Supplement: FIG S1 [file mbo002173254sf1.tif]

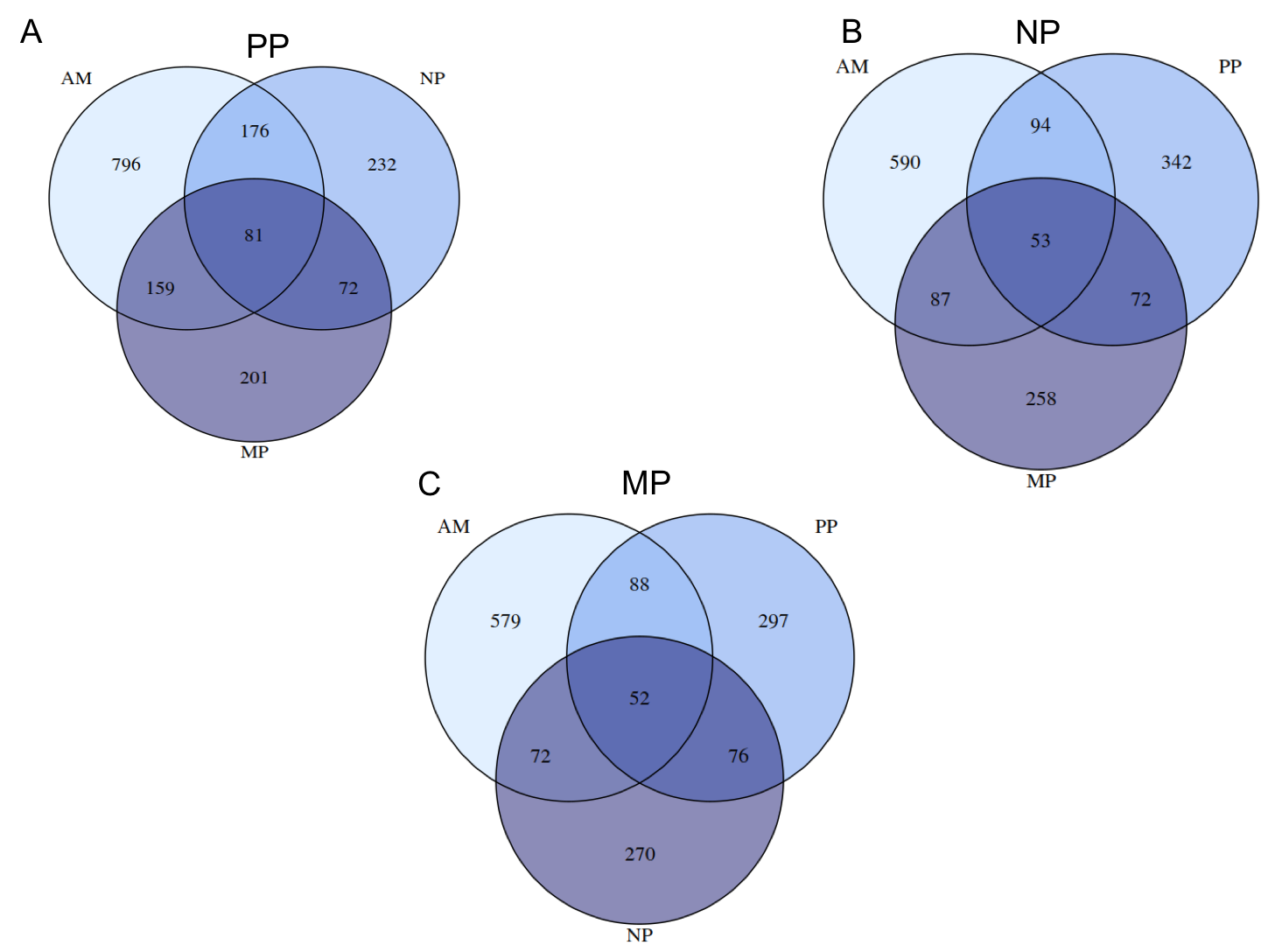

Supplement: FIG S2 [file mbo002173254sf2.tif]
