# Supplementary material for: The Transcriptome of Leishmania major Developmental Stages in Their Natural Sand Fly Vector
Source: mBio. 2017 Apr 4;8(2):e00029-17. doi: 10.1128/mBio.00029-17 (PMC5380837; doi:10.1128/mBio.00029-17)
Supplement: TABLE S2 [file mbo002173254st2.docx]

Table S2. Differentially expressed genes between the different samples

| **Samples** | **# DEG** | **>2-fold** | **>4-fold** |
| --- | --- | --- | --- |
| AM vs. PP | 1212 | 989 | 256 |
| AM vs. NP | 824 | 688 | 220 |
| AM vs. MP | 791 | 672 | 172 |
| PP vs. NP | 561 | 474 | 101 |
| PP vs. MP | 513 | 438 | 104 |
| NP vs. MP | 470 | 398 | 71 |
| MP vs. CMP | 26 | 26 | 6 |
| AM vs. CMP | 1076 | 822 | 154 |
