# Supplementary material for: The Transcriptome of Leishmania major Developmental Stages in Their Natural Sand Fly Vector
Source: mBio. 2017 Apr 4;8(2):e00029-17. doi: 10.1128/mBio.00029-17 (PMC5380837; doi:10.1128/mBio.00029-17)
Supplement: DATA SET S2 [file mbo002173254sd2.pdf]

A. Enrichment of GO categories, downregulated in PP vs. AM (5 categories)

Category	Adj pval[a]	Num in subset[b]	Num total[c]	Term	Ontology
GO:0016209	0.0000	7	8	antioxidant activity	MF
GO:0051920	0.0000	7	8	peroxiredoxin activity	MF
GO:0008234	0.0000	8	13	cysteine-type peptidase activity	CC
GO:0006308	0.000	6	9	DNA catabolic process	BP
GO:0004806	0.001	7	11	triglyceride lipase activity	MF

(a) The confidence that a given category is enriched in the developmental stage. (b) Number of genes from the pathway that were found in a given developmental stage. (c) the total number of genes with that annotation in the genome.

B. Enrichment of GO categories, Upregulated in PP vs. AM (18 categories)

Category	Adj pval	Num in subset	Num total	Term	Ontology
GO:0000786	0.0000	14	29	nucleosome	CC
GO:0003924	0.0000	24	86	GTPase activity	MF
GO:0015986	0.001	6	8	ATP synthesis coupled proton transport	
GO:0005516	0.0000	8	12	calmodulin binding	MF
GO:0009190	0.0000	9	12	cyclic nucleotide biosynthetic process	
GO:0035556	0.001	10	19	intracellular signal transduction	BP
GO:0005525	0.0000	26	128	GTP binding	MF
GO:0003677	0.003	32	161	DNA binding	MF
GO:0006334	0.0000	14	33	nucleosome assembly	BP
GO:0030286	0.0000	12	15	dynein complex	CC
GO:0031514	0.0000	11	28	motile cilium	CC
GO:0043234	0.0000	24	36	protein complex	CC
GO:0046982	0.0000	14	31	protein heterodimerization activity	MF
GO:0051258	0.0000	24	35	protein polymerization	BP
GO:0016849	0.0000	9	12	phosphorus-oxygen lyase activity	MF
GO:0004198	0.033	8	23	calcium-dependent cysteine-type endopeptidase	MF
GO:0006508	0.005	22	91	proteolysis	BP
GO:0000275	0.005	4	4	mitochondrial proton-transporting ATP synthase	CC

C. Enrichment of GO categories, downregulated in NP vs. PP (7 categories)

Category	Adj pval	Num in subset	Num total	Term	Ontology
GO:0000786	0.000	16	29	nucleosome	CC
GO:0046982	0.000	16	31	protein heterodimerization	MF
GO:0006334	0.000	16	33	nucleosome assembly	BP
GO:0003677	0.000	24	161	DNA binding	MF
GO:0005634	0.000	19	149	nucleus	CC
GO:0000776	0.005	4	5	kinetochore	CC
GO:0005516	0.009	5	12	calmodulin binding	MF

D. Enrichment of GO categories, downregulated in MP vs. NP (2 categories)

Category	Adj pval	Num in subset	Num total	Term	Ontology
GO:0006364	0.025	6	15	rRNA processing	BP
GO:0032040	0.049	4	6	small-subunit processome	CC

E. Enrichment of GO categories, upregulation in MP vs. PP (14 categories)

Category	Adj pval	Num in subset	Num total	Term	Ontology
GO:0009190	0.004	6	12	cyclic nucleotide biosynthetic process	BP
GO:0016849	0.004	6	12	phosphorus-oxygen lyase activity	MF
GO:0022857	0.004	4	6	transmembrane transporter activity	MF
GO:0002036	0.004	3	3	regulation of L-glutamate transport	BP
GO:0015193	0.004	3	3	L-proline transmembrane transporter activity	MF
GO:0015893	0.004	3	3	drug transport	BP
GO:0022858	0.004	3	3	alanine transmembrane transporter activity	MF
GO:0035524	0.004	3	3	proline transmembrane transport	BP
GO:0051955	0.004	3	3	regulation of amino acid transport	BP
GO:0070881	0.004	3	3	regulation of proline transport	BP
GO:0080144	0.004	3	3	amino acid homeostasis	BP
GO:0006971	0.013	3	4	hypotonic response	BP
GO:0035556	19 0.013	6	19	intracellular signal transduction	BP
GO:0005337	0.036	3	5	nucleoside transmembrane transporter activity	MF

F. Enrichment of GO categories, downregulated in MP vs. PP (8 categories)

Category	Adj pval	Num in subset	Num total	Term	Ontology
GO:0003677	0.002	18	161	DNA binding	MF
GO:0000786	0.002	8	29	nucleosome	CC
GO:0046982	0.002	8	31	protein heterodimerization	MF
GO:0006334	0.003	8	33	nucleosome assembly	BP
GO:0044267	0.007	5	12	cellular protein metabolic process	BP
GO:0015991	0.018	6	23	ATP hydrolysis coupled proton transport	BP
GO:0051258	0.023	7	35	protein polymerization	BP
GO:0043234	0.024	7	36	protein complex	CC

G. Enrichment of GO categories, Upregulated in MP vs. AM (34 categories)

Category	Adj pval	Num in subset	Num total	Term	Ontology
GO:0000786	0.000	19	29	nucleosome	CC
GO:0046982	0.000	19	31	protein heterodimerization activity	MF
GO:0006334	0.000	19	33	nucleosome assembly	BP
GO:0051258	0.000	18	35	protein polymerization	BP
GO:0043234	0.000	18	36	protein complex	CC
GO:0009190	0.000	9	12	cyclic nucleotide biosynthetic process	BP
GO:0016849	0.000	9	12	phosphorus-oxygen lyase activity	MF
GO:0003924	0.000	18	86	GTPase activity	MF
GO:0031514	0.000	12	28	motile cilium	CC
GO:0005516	0.000	7	12	calmodulin binding	MF
GO:0035556	0.000	9	19	intracellular signal transduction	BP
GO:0022857	0.000	5	6	transmembrane transporter activity	MF
GO:0005337	0.006	4	5	nucleoside transmembrane transporter activity	MF
GO:0007155	0.010	4	6	cell adhesion	BP
GO:0002036	0.010	3	3	regulation of L-glutamate transport	BP
GO:0015193	0.010	3	3	L-proline transmembrane transporter activity	MF
GO:0015893	0.010	3	3	drug transport	BP
GO:0022858	0.010	3	3	alanine transmembrane transporter activity	MF
GO:0035524	0.010	3	3	proline transmembrane transport	BP
GO:0051955	0.010	3	3	regulation of amino acid transport	BP
GO:0070881	0.010	3	3	regulation of proline transport	BP
GO:0080144	0.010	3	3	amino acid homeostasis	BP
GO:0008410	0.010	3	3	CoA-transferase activity	MF
GO:0046952	0.010	3	3	ketone body catabolic process	BP
GO:0005353	0.010	3	3	fructose transmembrane transporter activity	MF
GO:0005354	0.010	3	3	galactose transmembrane transporter activity	MF
GO:0015578	0.010	3	3	mannose transmembrane transporter activity	MF
GO:0015758	0.010	3	3	glucose transport	BP

GO:0005525	0.013	18	129	GTP binding	MF
GO:0006971	0.031	3	3	hypotonic response	BP
GO:0008160	0.034	3	4	protein tyrosine phosphatase activator activity	MF
GO:0042025	0.034	3	4	host cell nucleus	CC
GO:0044081	0.034	3	4	modulation by symbiont of host nitric oxide-mediated	BP
GO:0075130	0.034	3	4	modulation by symbiont of host protein kinase-m	BP

H. Enrichment of GO categories, downregulated in MP vs. AM (6 categories)

**Category**	**Adj pval**	**Num in subset**	**Num total**	**Term**	**Ontology**
GO:0016209	0.000	6	8	antioxidant activity	MF
GO:0051920	0.000	6	8	peroxiredoxin activity	MF
GO:0008234	0.000	7	13	cysteine-type peptidase activity	MF
GO:0006308	0.002	5	9	DNA catabolic process	BP
GO:0004197	0.014	4	7	cysteine-type endopeptidase activity	MF
GO:0004519	0.026	5	5	endonuclease activity	MF

I. Enrichment of GO categories, Upregulated in NP vs. AM (24 categories)

Category	Adj pval	Num in subset	Num total	Term	Ontology
GO:0051258	0.000	17	35	protein polymerization	BP
GO:0043234	0.000	17	36	protein complex	CC
GO:0009190	0.000	8	12	cyclic nucleotide biosynthetic process	BP
GO:0016849	0.000	8	12	phosphorus-oxygen lyase activity	MF
GO:0003924	0.001	18	86	GTPase activity	MF
GO:0000786	0.001	8	29	nucleosome	CC
GO:0046982	0.002	8	31	protein heterodimerization	MF
GO:0006508	0.004	19	91	proteolysis	BP
GO:0006334	0.004	8	33	nucleosome assembly	BP
GO:0035556	0.004	8	19	intracellular signal transduction	BP
GO:0005516	0.009	6	12	calmodulin binding	MF
GO:0005525	0.012	20	129	GTP binding	MF
GO:0005886	0.017	6	15	plasma membrane	CC
GO:0002036	0.017	3	3	regulation of L-glutamate transport	BP
GO:0015193	0.017	3	3	proline transmembrane transporter activity	MF
GO:0080144	0.017	3	3	amino acid homeostasis	BP
GO:0008410	0.017	3	3	CoA-transferase activity	MF
GO:0046952	0.017	3	3	ketone body catabolic process	BP
GO:0022857	0.017	4	6	transmembrane transporter	MF
GO:0031514	0.017	9	28	motile cilium	CC
GO:0005353	0.017	3	3	fructose transmembrane transporter activity	MF
GO:0005354	0.017	3	3	galactose transmembrane transporter activity	MF
GO:0015578	0.017	3	3	mannose transmembrane transporter activity	MF
GO:0015758	0.017	3	3	glucose transport	BP

J. Enrichment of GO categories, downregulated in NP vs. AM (7 categories)

Category	Adj pval	Num in subset	Num total	Term	Ontology
GO:0016209	0.0000	7	8	antioxidant activity	MF
GO:0051920	0.0000	7	8	peroxiredoxin activity	MF
GO:0008234	0.0000	8	13	cysteine-type peptidase activity	CC
GO:0004519	0.006	6	15	endonuclease activity	MF
GO:0006308	0.000	6	9	DNA catabolic process	BP
GO:0004197	0.024	4	7	cysteine-type endopeptidase activity	MF
GO:0008378	0.049	4	8	galactosyltransferase activity	MF
