# Supplementary material for: The Transcriptome of Leishmania major Developmental Stages in Their Natural Sand Fly Vector
Source: mBio. 2017 Apr 4;8(2):e00029-17. doi: 10.1128/mBio.00029-17 (PMC5380837; doi:10.1128/mBio.00029-17)
Supplement: DATA SET S3 [file mbo002173254sd3.pdf]

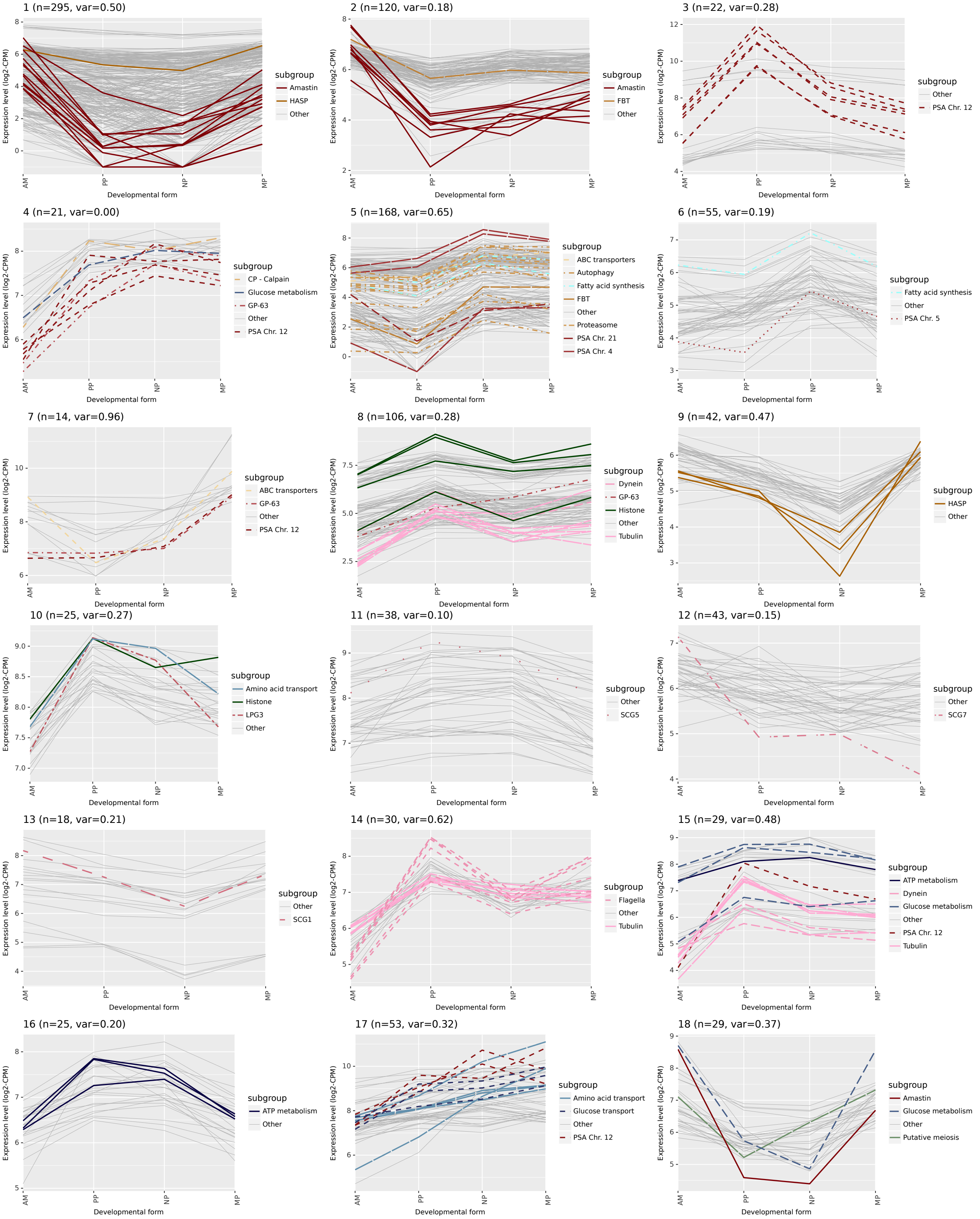

1 (n=295, var=0.50)
subgroup
Amastin
HASP
Other
2 (n=120, var=0.18)
subgroup
Amastin
FBT
Other
3 (n=22, var=0.28)
subgroup
Other
PSA Chr. 12
4 (n=21, var=0.00)
subgroup
CP - Calpain
Glucose metabolism
GP-63
Other
PSA Chr. 12
5 (n=168, var=0.65)
subgroup
ABC transporters
Autophagy
Fatty acid synthesis
FBT
Other
Proteasome
PSA Chr. 21
PSA Chr. 4
6 (n=55, var=0.19)
subgroup
Fatty acid synthesis
Other
PSA Chr. 5
7 (n=14, var=0.96)
subgroup
ABC transporters
GP-63
Other
PSA Chr. 12
8 (n=106, var=0.28)
subgroup
Dynein
GP-63
Histone
Other
Tubulin
9 (n=42, var=0.47)
subgroup
HASP
Other
10 (n=25, var=0.27)
subgroup
Amino acid transport
Histone
LPG3
Other
11 (n=38, var=0.10)
subgroup
Other
SCG5
12 (n=43, var=0.15)
subgroup
Other
SCG7
13 (n=18, var=0.21)
subgroup
Other
SCG1
14 (n=30, var=0.62)
subgroup
Flagella
Other
Tubulin
15 (n=29, var=0.48)
subgroup
ATP metabolism
Dynein
Glucose metabolism
Other
PSA Chr. 12
Tubulin
16 (n=25, var=0.20)
subgroup
ATP metabolism
Other
17 (n=53, var=0.32)
subgroup
Amino acid transport
Glucose transport
Other
PSA Chr. 12
18 (n=29, var=0.37)
subgroup
Amastin
Glucose metabolism
Other
Putative meiosis
Expression level (log2-CPM)
Developmental form
AM
PP
NP
MP

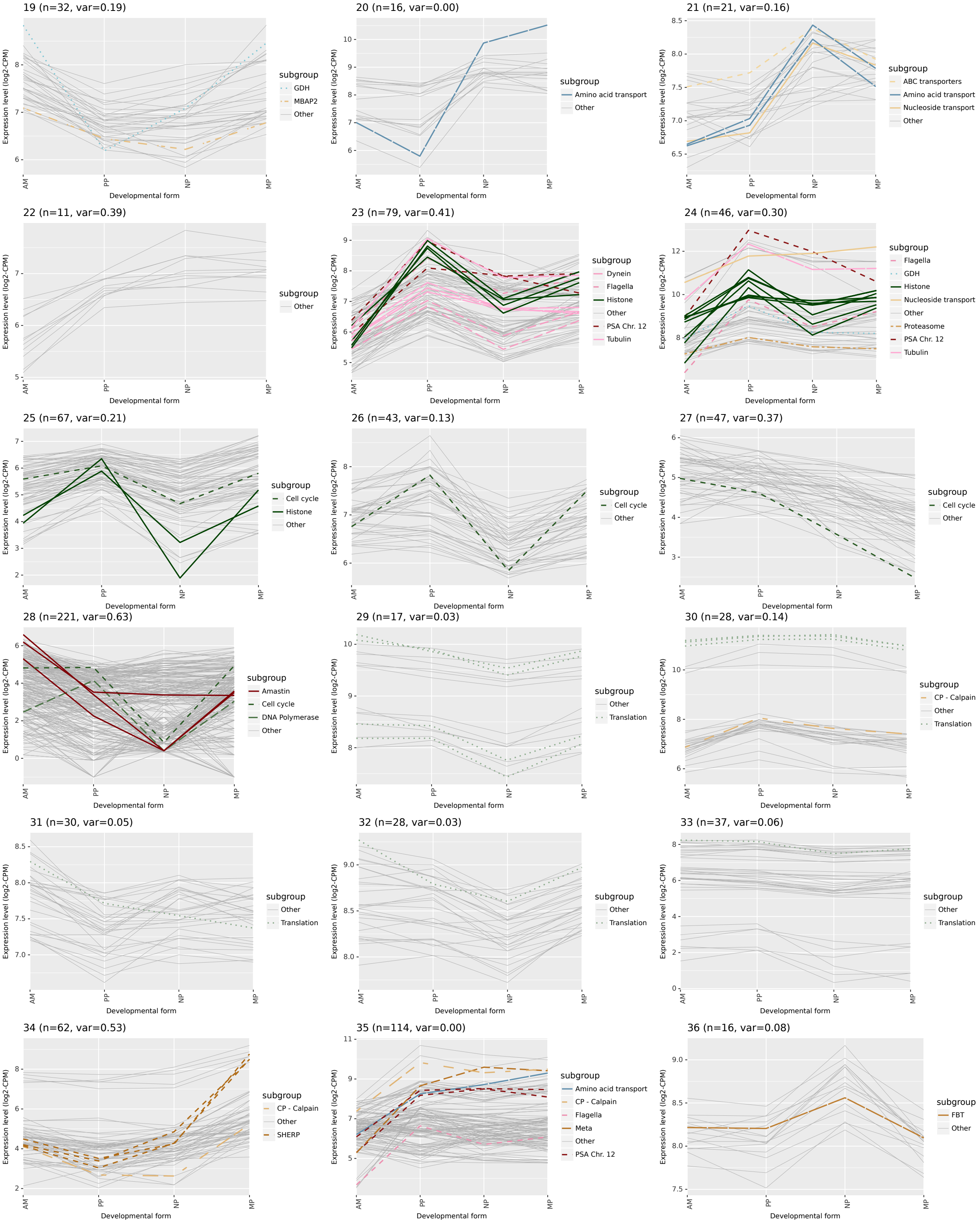
19 (n=32, var=0.19)
subgroup
GDH
MBAP2
Other
20 (n=16, var=0.00)
subgroup
Amino acid transport
Other
21 (n=21, var=0.16)
subgroup
ABC transporters
Amino acid transport
Nucleoside transport
Other
22 (n=11, var=0.39)
subgroup
Other
23 (n=79, var=0.41)
subgroup
Dynein
Flagella
Histone
Other
PSA Chr. 12
Tubulin
24 (n=46, var=0.30)
subgroup
Flagella
GDH
Histone
Nucleoside transport
Other
Proteasome
PSA Chr. 12
Tubulin
25 (n=67, var=0.21)
subgroup
Cell cycle
Histone
Other
26 (n=43, var=0.13)
subgroup
Cell cycle
Other
27 (n=47, var=0.37)
subgroup
Cell cycle
Other
28 (n=221, var=0.63)
subgroup
Amastin
Cell cycle
DNA Polymerase
Other
29 (n=17, var=0.03)
subgroup
Other
Translation
30 (n=28, var=0.14)
subgroup
CP - Calpain
Other
Translation
31 (n=30, var=0.05)
subgroup
Other
Translation
32 (n=28, var=0.03)
subgroup
Other
Translation
33 (n=37, var=0.06)
subgroup
Other
Translation
34 (n=62, var=0.53)
subgroup
CP - Calpain
Other
SHERP
35 (n=114, var=0.00)
subgroup
Amino acid transport
CP - Calpain
Flagella
Meta
Other
PSA Chr. 12
36 (n=16, var=0.08)
subgroup
FBT
Other
Expression level (log2-CPM)
Developmental form
AM
PP
NP
MP

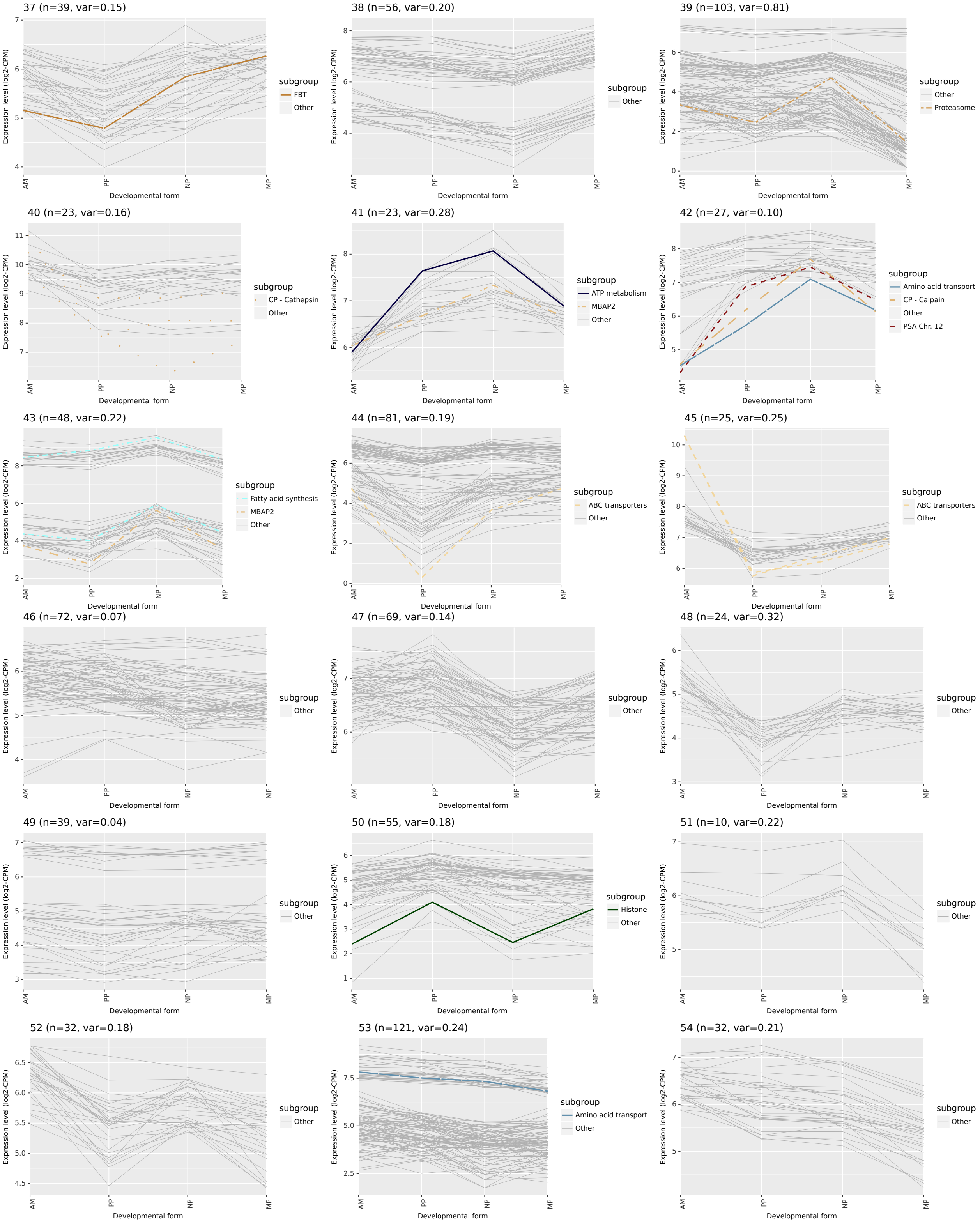
37 (n=39, var=0.15)
subgroup
FBT
Other
38 (n=56, var=0.20)
subgroup
Other
39 (n=103, var=0.81)
subgroup
Other
Proteasome
40 (n=23, var=0.16)
subgroup
CP - Cathepsin
Other
41 (n=23, var=0.28)
subgroup
ATP metabolism
MBAP2
Other
42 (n=27, var=0.10)
subgroup
Amino acid transport
CP - Calpain
Other
PSA Chr. 12
43 (n=48, var=0.22)
subgroup
Fatty acid synthesis
MBAP2
Other
44 (n=81, var=0.19)
subgroup
ABC transporters
Other
45 (n=25, var=0.25)
subgroup
ABC transporters
Other
46 (n=72, var=0.07)
subgroup
Other
47 (n=69, var=0.14)
subgroup
Other
48 (n=24, var=0.32)
subgroup
Other
49 (n=39, var=0.04)
subgroup
Other
50 (n=55, var=0.18)
subgroup
Histone
Other
51 (n=10, var=0.22)
subgroup
Other
52 (n=32, var=0.18)
subgroup
Other
53 (n=121, var=0.24)
subgroup
Amino acid transport
Other
54 (n=32, var=0.21)
subgroup
Other
Expression level (log2-CPM)
Developmental form
AM
PP
NP
MP

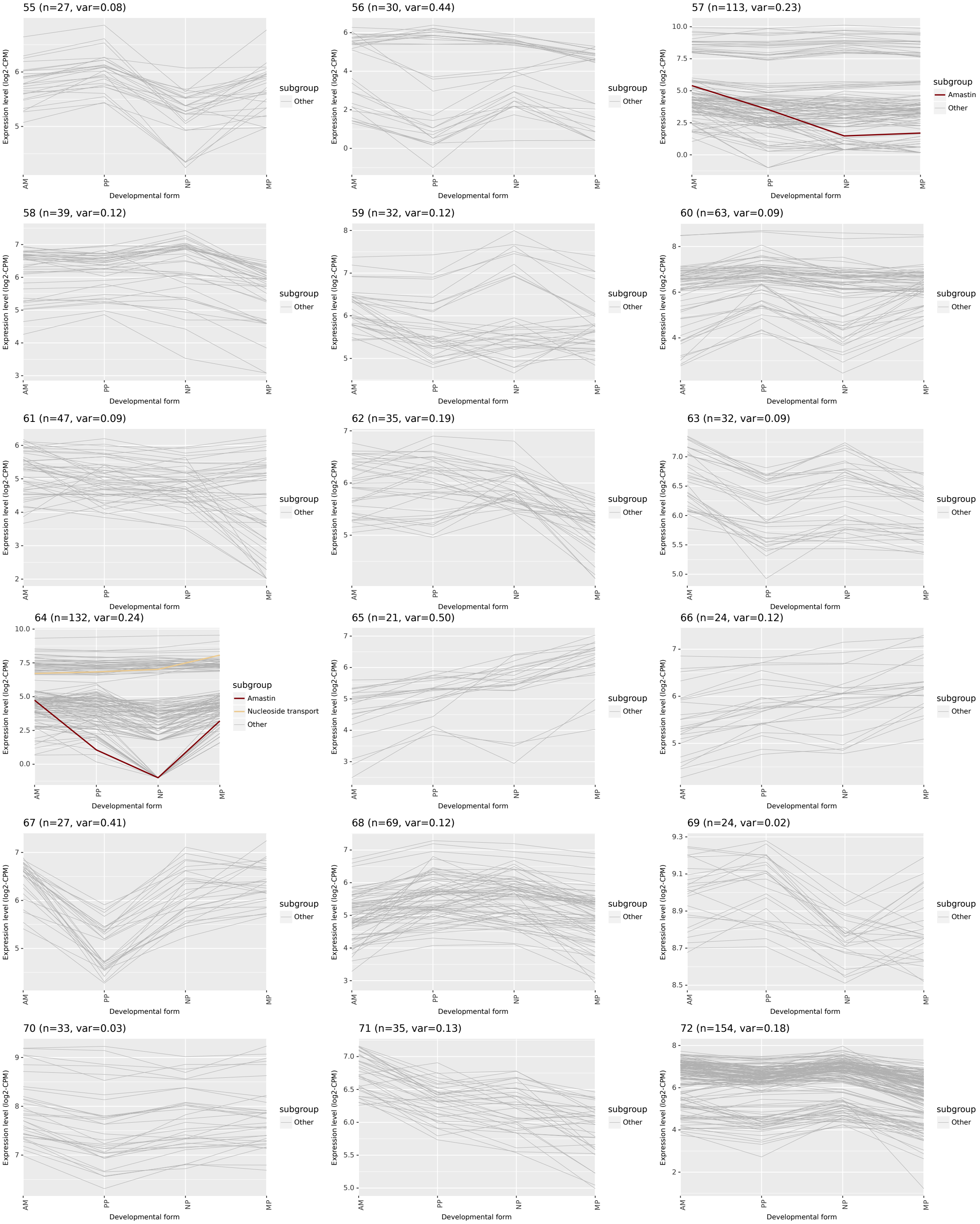
55 (n=27, var=0.08)
56 (n=30, var=0.44)
57 (n=113, var=0.23)
58 (n=39, var=0.12)
59 (n=32, var=0.12)
60 (n=63, var=0.09)
61 (n=47, var=0.09)
62 (n=35, var=0.19)
63 (n=32, var=0.09)
64 (n=132, var=0.24)
65 (n=21, var=0.50)
66 (n=24, var=0.12)
67 (n=27, var=0.41)
68 (n=69, var=0.12)
69 (n=24, var=0.02)
70 (n=33, var=0.03)
71 (n=35, var=0.13)
72 (n=154, var=0.18)
Expression level (log2-CPM)
Developmental form
AM
PP
NP
MP
subgroup
Amastin
Nucleoside transport
Other

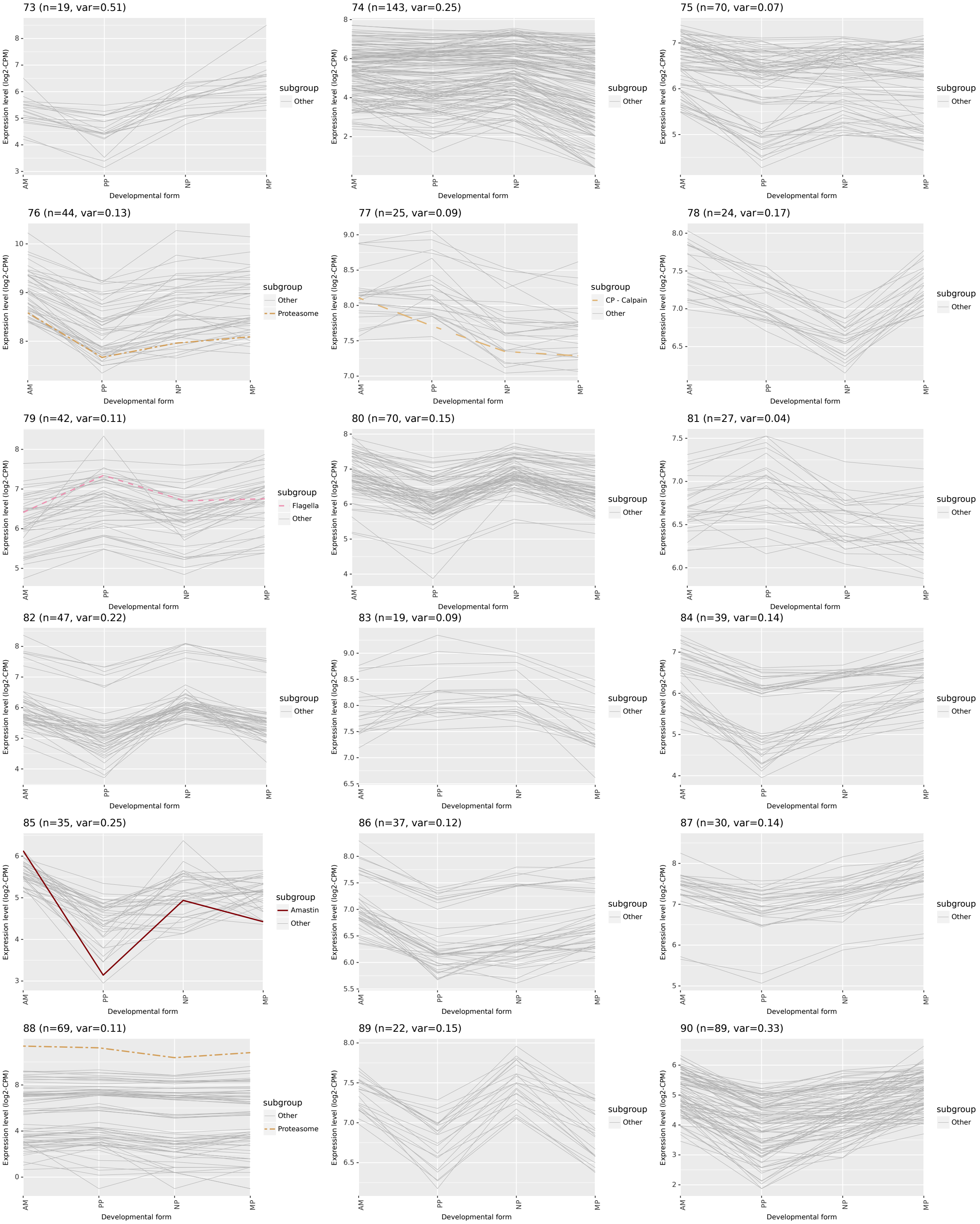
73 (n=19, var=0.51)
74 (n=143, var=0.25)
75 (n=70, var=0.07)
76 (n=44, var=0.13)
77 (n=25, var=0.09)
78 (n=24, var=0.17)
79 (n=42, var=0.11)
80 (n=70, var=0.15)
81 (n=27, var=0.04)
82 (n=47, var=0.22)
83 (n=19, var=0.09)
84 (n=39, var=0.14)
85 (n=35, var=0.25)
86 (n=37, var=0.12)
87 (n=30, var=0.14)
88 (n=69, var=0.11)
89 (n=22, var=0.15)
90 (n=89, var=0.33)
Expression level (log2-CPM)
Developmental form
AM
PP
NP
MP
subgroup
Other
Proteasome
CP - Calpain
Flagella
Amastin

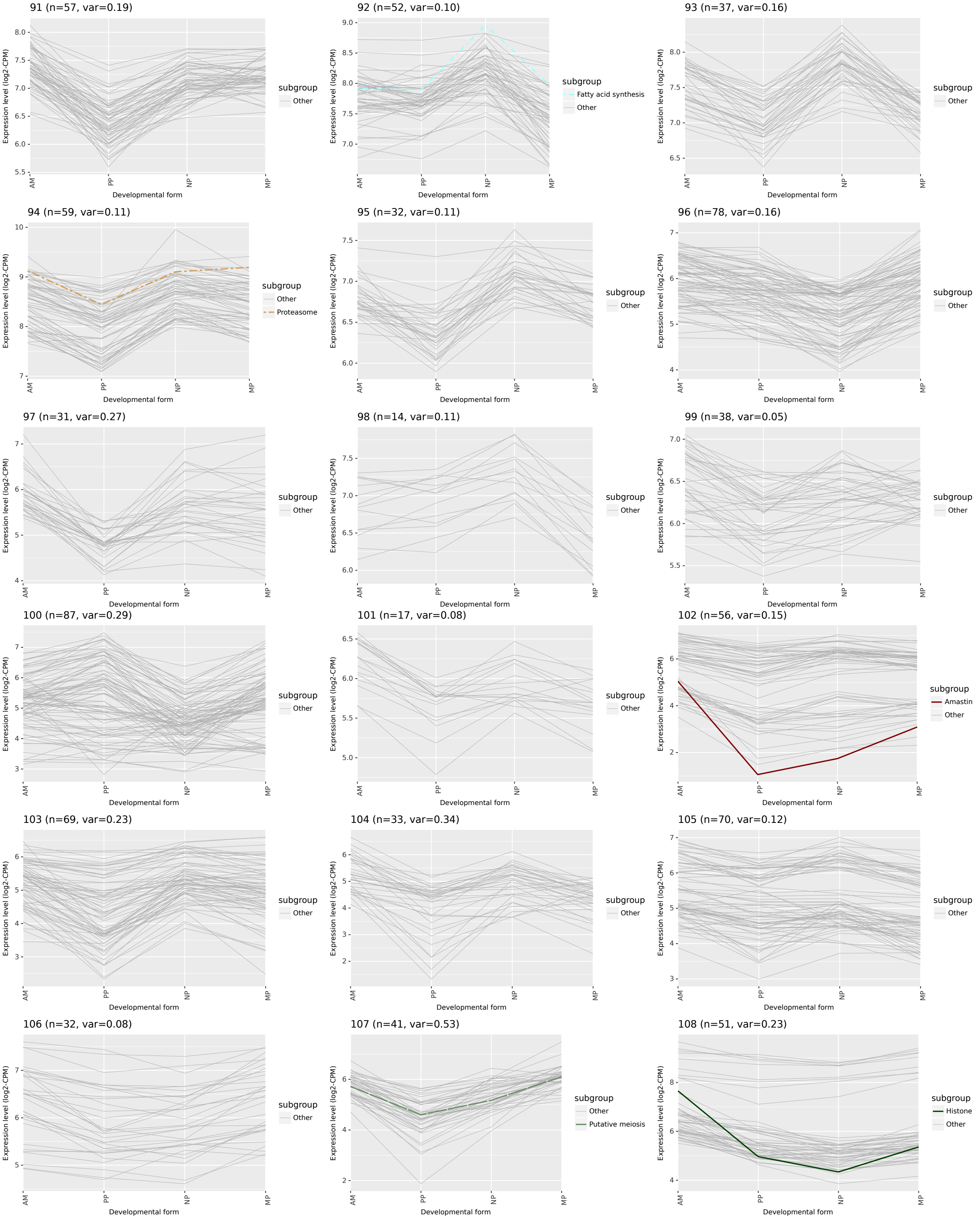
91 (n=57, var=0.19)
subgroup
Other
92 (n=52, var=0.10)
subgroup
Fatty acid synthesis
Other
93 (n=37, var=0.16)
subgroup
Other
94 (n=59, var=0.11)
subgroup
Other
Proteasome
95 (n=32, var=0.11)
subgroup
Other
96 (n=78, var=0.16)
subgroup
Other
97 (n=31, var=0.27)
subgroup
Other
98 (n=14, var=0.11)
subgroup
Other
99 (n=38, var=0.05)
subgroup
Other
100 (n=87, var=0.29)
subgroup
Other
101 (n=17, var=0.08)
subgroup
Other
102 (n=56, var=0.15)
subgroup
Amastin
Other
103 (n=69, var=0.23)
subgroup
Other
104 (n=33, var=0.34)
subgroup
Other
105 (n=70, var=0.12)
subgroup
Other
106 (n=32, var=0.08)
subgroup
Other
107 (n=41, var=0.53)
subgroup
Other
Putative meiosis
108 (n=51, var=0.23)
subgroup
Histone
Other
Expression level (log2-CPM)
Developmental form
AM
PP
NP
MP

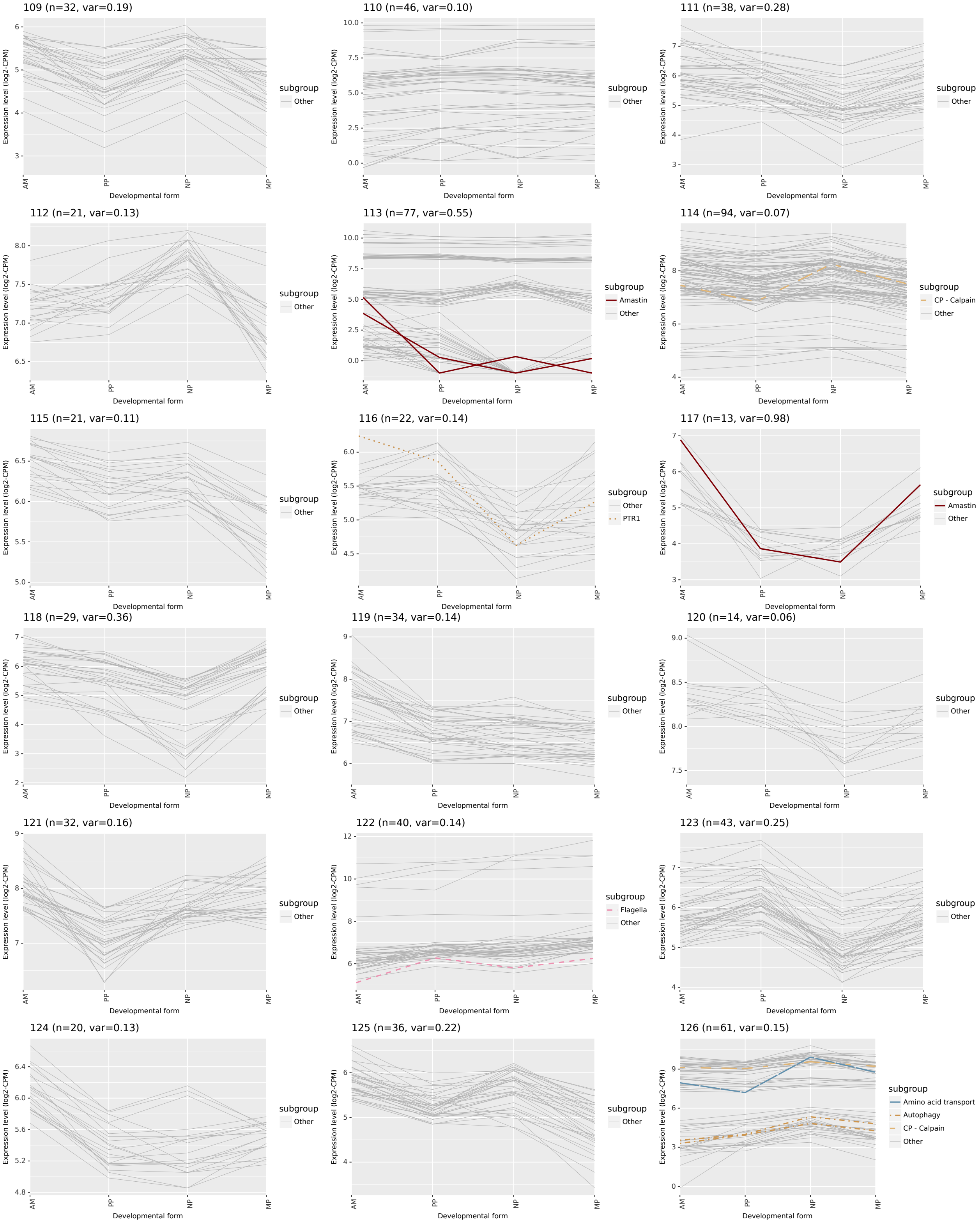
109 (n=32, var=0.19)
110 (n=46, var=0.10)
111 (n=38, var=0.28)
112 (n=21, var=0.13)
113 (n=77, var=0.55)
114 (n=94, var=0.07)
115 (n=21, var=0.11)
116 (n=22, var=0.14)
117 (n=13, var=0.98)
118 (n=29, var=0.36)
119 (n=34, var=0.14)
120 (n=14, var=0.06)
121 (n=32, var=0.16)
122 (n=40, var=0.14)
123 (n=43, var=0.25)
124 (n=20, var=0.13)
125 (n=36, var=0.22)
126 (n=61, var=0.15)
Expression level (log2-CPM)
Developmental form
AM
PP
NP
MP
subgroup
Other
Amastin
CP - Calpain
PTR1
Flagella
Amino acid transport
Autophagy

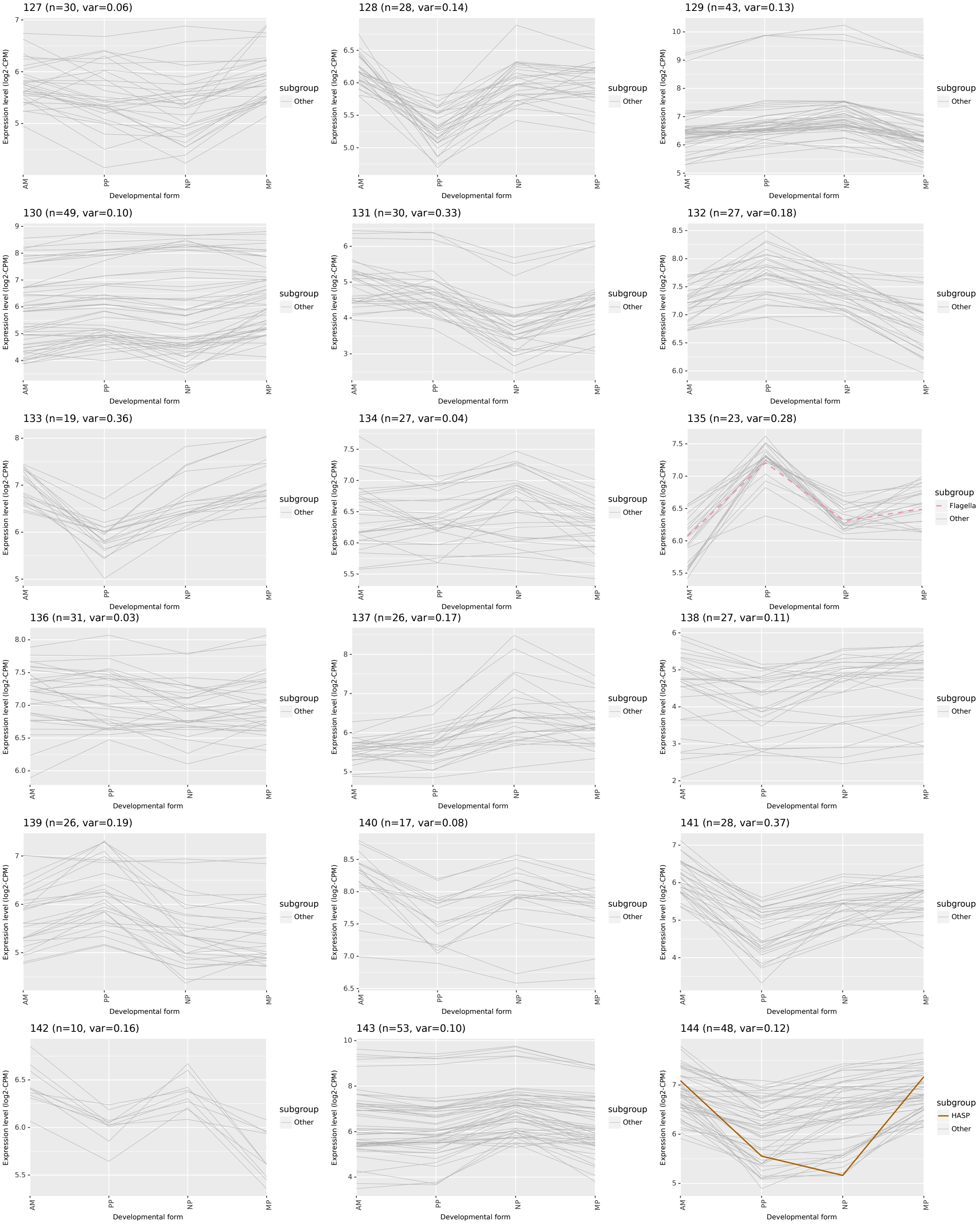
127 (n=30, var=0.06)
128 (n=28, var=0.14)
129 (n=43, var=0.13)
130 (n=49, var=0.10)
131 (n=30, var=0.33)
132 (n=27, var=0.18)
133 (n=19, var=0.36)
134 (n=27, var=0.04)
135 (n=23, var=0.28)
136 (n=31, var=0.03)
137 (n=26, var=0.17)
138 (n=27, var=0.11)
139 (n=26, var=0.19)
140 (n=17, var=0.08)
141 (n=28, var=0.37)
142 (n=10, var=0.16)
143 (n=53, var=0.10)
144 (n=48, var=0.12)
Expression level (log2-CPM)
Developmental form
AM
PP
NP
MP
subgroup
Other
Flagella
HASP

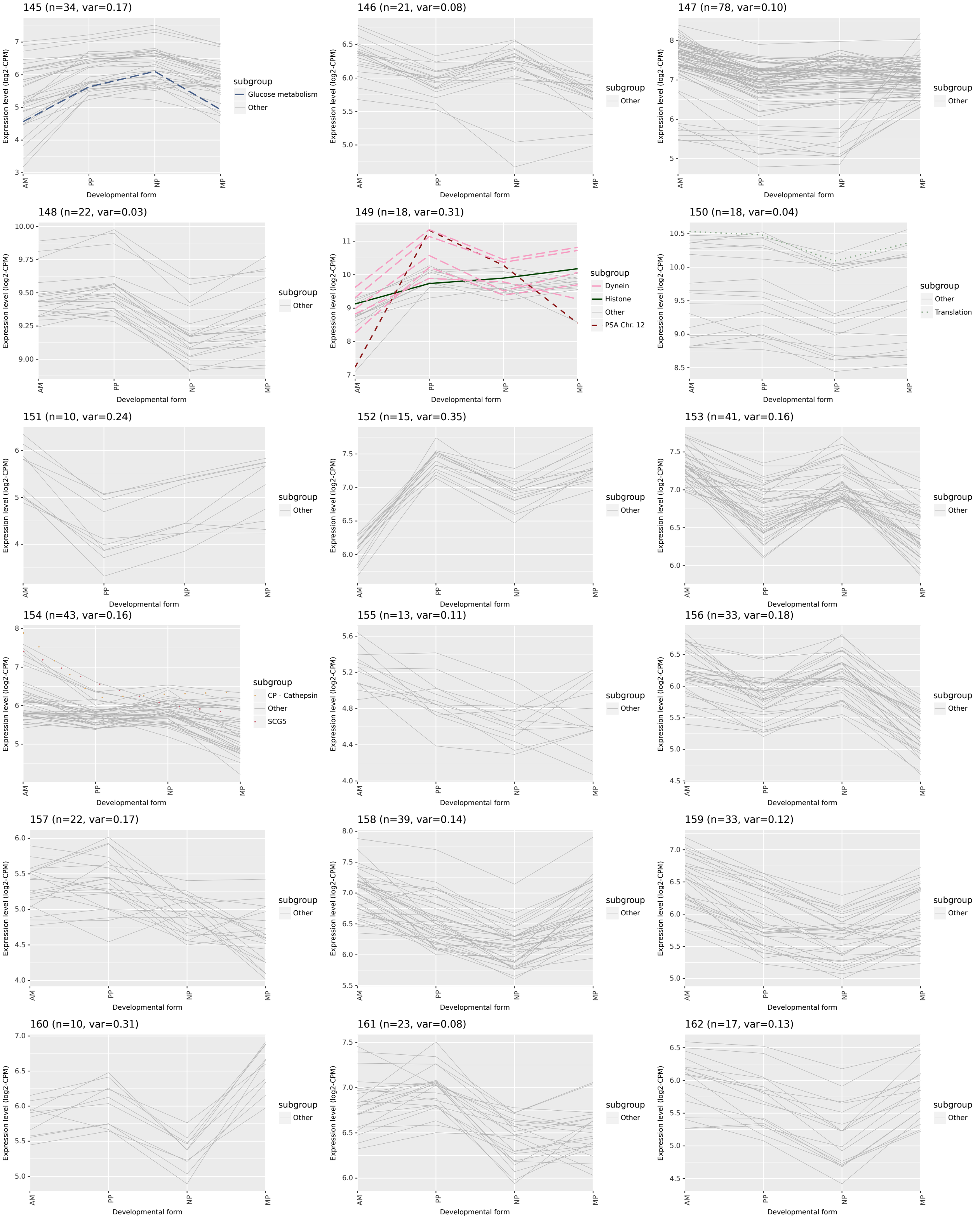
145 (n=34, var=0.17)
146 (n=21, var=0.08)
147 (n=78, var=0.10)
148 (n=22, var=0.03)
149 (n=18, var=0.31)
150 (n=18, var=0.04)
151 (n=10, var=0.24)
152 (n=15, var=0.35)
153 (n=41, var=0.16)
154 (n=43, var=0.16)
155 (n=13, var=0.11)
156 (n=33, var=0.18)
157 (n=22, var=0.17)
158 (n=39, var=0.14)
159 (n=33, var=0.12)
160 (n=10, var=0.31)
161 (n=23, var=0.08)
162 (n=17, var=0.13)
Expression level (log2-CPM)
Developmental form
AM
PP
NP
MP
subgroup
Glucose metabolism
Other
Dynein
Histone
PSA Chr. 12
Translation
CP - Cathepsin
SCG5

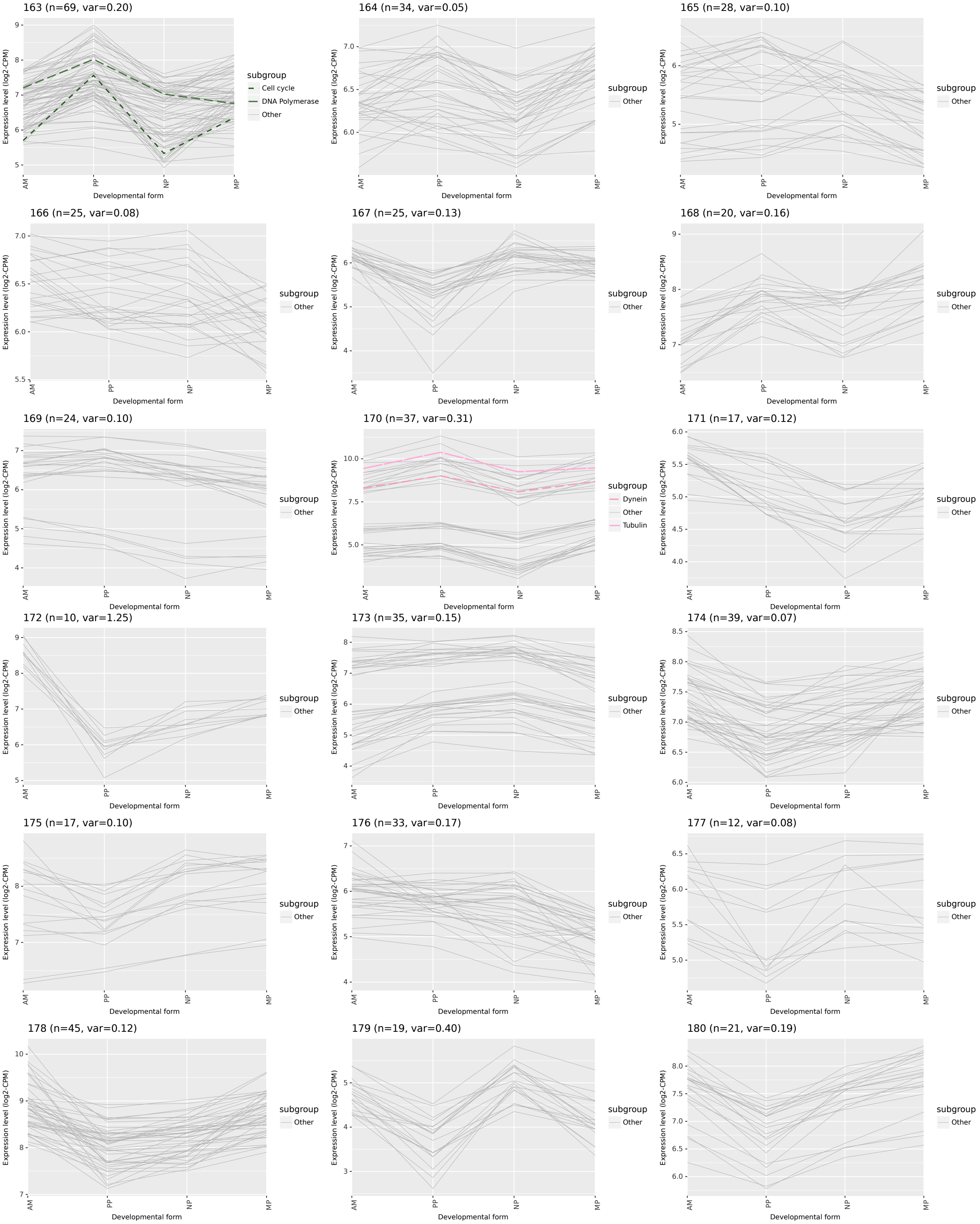
163 (n=69, var=0.20)
subgroup
Cell cycle
DNA Polymerase
Other
164 (n=34, var=0.05)
165 (n=28, var=0.10)
166 (n=25, var=0.08)
167 (n=25, var=0.13)
168 (n=20, var=0.16)
169 (n=24, var=0.10)
170 (n=37, var=0.31)
Dynein
Tubulin
171 (n=17, var=0.12)
172 (n=10, var=1.25)
173 (n=35, var=0.15)
174 (n=39, var=0.07)
175 (n=17, var=0.10)
176 (n=33, var=0.17)
177 (n=12, var=0.08)
178 (n=45, var=0.12)
179 (n=19, var=0.40)
180 (n=21, var=0.19)
Expression level (log2-CPM)
Developmental form
AM
PP
NP
MP

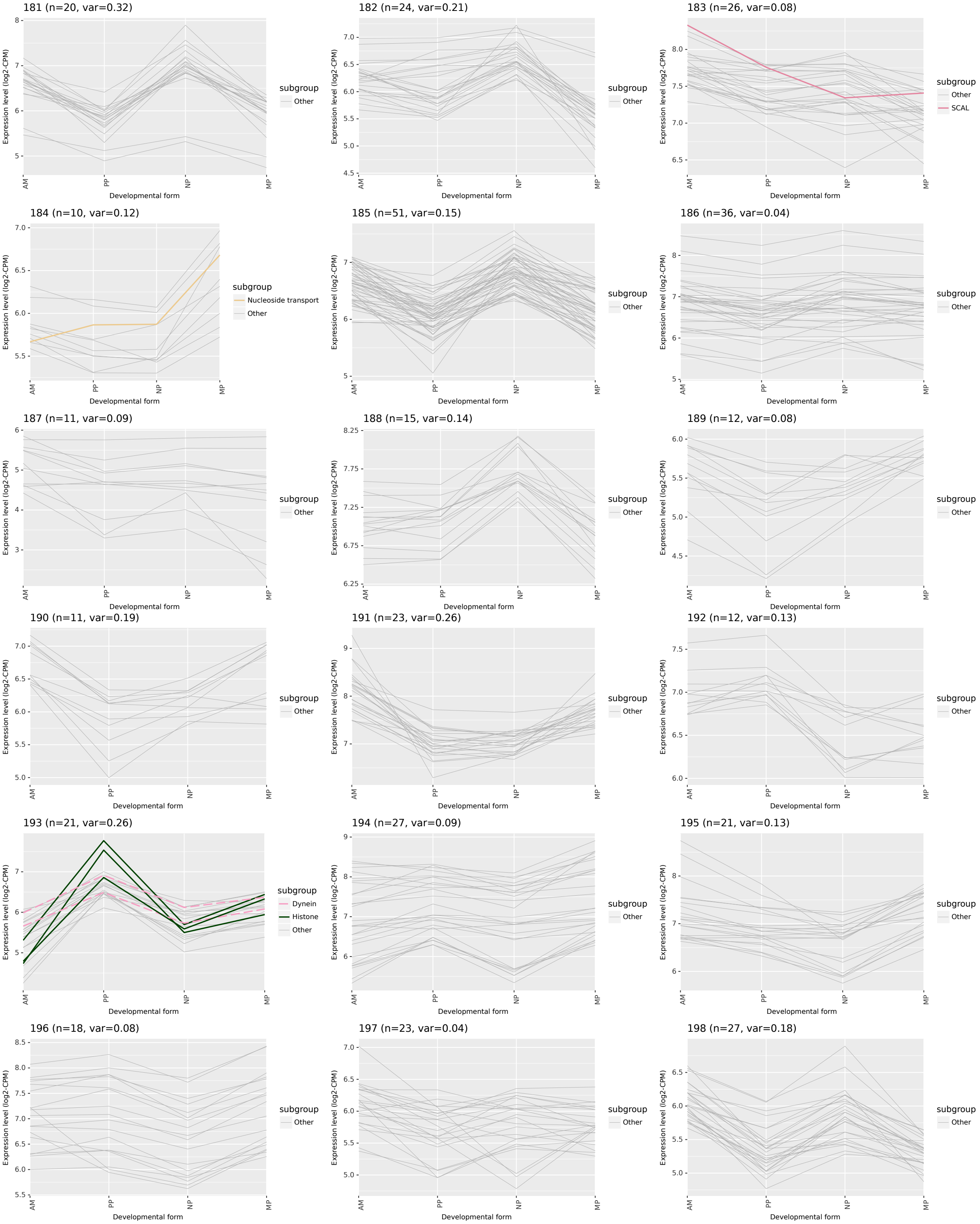
181 (n=20, var=0.32)
182 (n=24, var=0.21)
183 (n=26, var=0.08)
184 (n=10, var=0.12)
185 (n=51, var=0.15)
186 (n=36, var=0.04)
187 (n=11, var=0.09)
188 (n=15, var=0.14)
189 (n=12, var=0.08)
190 (n=11, var=0.19)
191 (n=23, var=0.26)
192 (n=12, var=0.13)
193 (n=21, var=0.26)
194 (n=27, var=0.09)
195 (n=21, var=0.13)
196 (n=18, var=0.08)
197 (n=23, var=0.04)
198 (n=27, var=0.18)
Expression level (log2-CPM)
Developmental form
AM
PP
NP
MP
subgroup
Other
SCAL
Nucleoside transport
Dynein
Histone

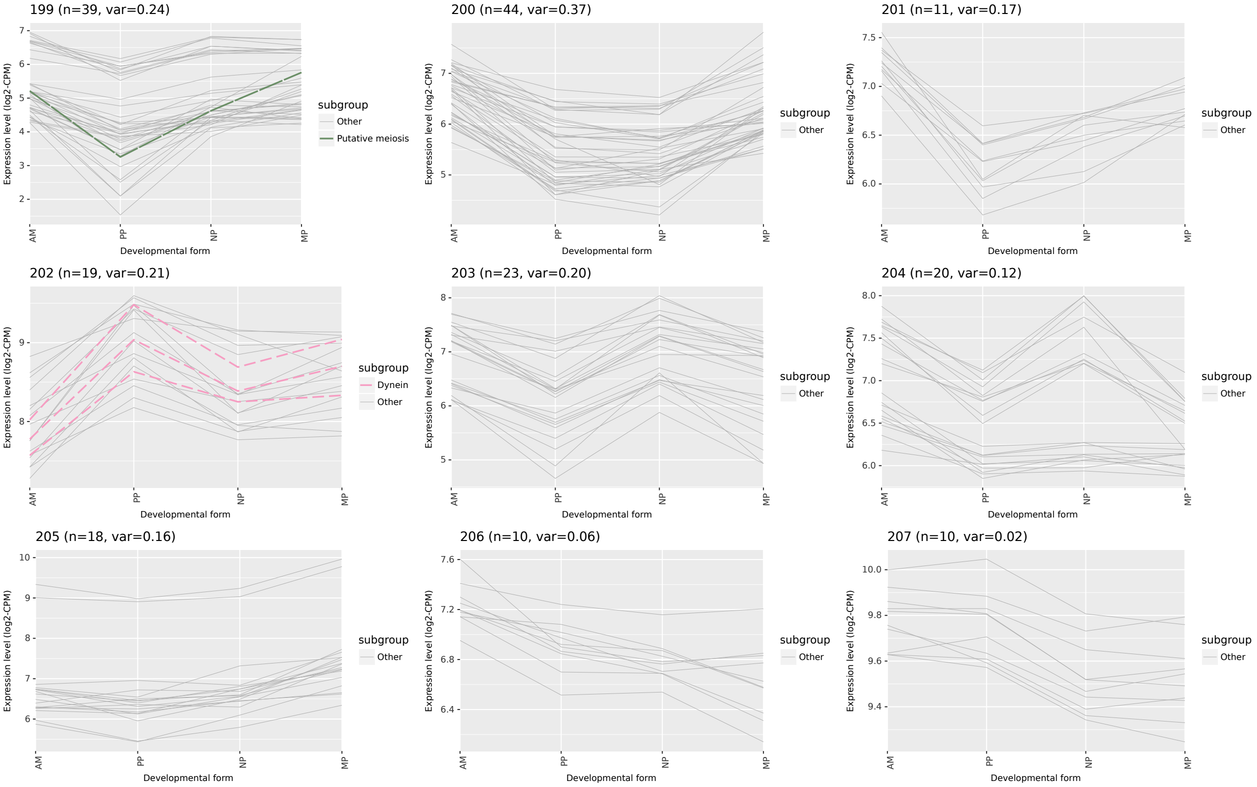

Two hundred and seven co-expression modules for genes with similar mRNA abundance patterns across the different developmental stages. Each line represents a gene, with genes of particular interest labeled with different colors and/or line patterns. The CPM (counts per million) represents the proportion of reads mapped to each Leishmania gene multiplied by one million.
