# Supplementary material for: The Transcriptome of Leishmania major Developmental Stages in Their Natural Sand Fly Vector
Source: mBio. 2017 Apr 4;8(2):e00029-17. doi: 10.1128/mBio.00029-17 (PMC5380837; doi:10.1128/mBio.00029-17)
Supplement: DATA SET S5 [file mbo002173254sd5.pdf]

Enrichment of GO categories; over represented in module 4

category	Adj pval[a]	Num in subset[b]	Num total[c]	Term	Ontology
GO:0004198	0.0328	3	23	calcium-dependent cysteine-type endopeptidase activity	MF
GO:0006508	0.0132	5	91	proteolysis	BP
GO:0007155	0.0328	2	6	cell adhesion	BP
GO:0008160	0.0164	2	4	protein tyrosine phosphatase activator activity	MF
GO:0042025	0.0164	2	4	host cell nucleus	CC
GO:0044081	0.0164	2	4	modulation by symbiont of host nitric oxide-mediated signal transduction	BP
GO:0075130	0.0164	2	4	modulation by symbiont of host protein kinase-mediated signal transduction	BP

(a**) The confidence that a given category is enriched in the developmental stage. (b) Number of genes from the pathway that were found in a given developmental stage. (c) the total number of genes with that annotation in the genome.**

Enrichment of GO categories; over represented in module 8

	Adj pval	Num in subset	Num total	Term	Ontology
GO:0000786	0.0282	5	29	nucleosome	CC
GO:0003924	0.0018	9	86	GTPase activity	MF
GO:0005525	0.0282	9	128	GTP binding	MF
GO:0006334	0.0338	5	33	nucleosome assembly	BP
GO:0043234	0.0000	8	36	protein complex	CC
GO:0046982	0.0293	5	31	protein heterodimerization activity	MF
GO:0051258	0.0000	8	35	protein polymerization	BP

Enrichment of GO categories; over represented in module 14

Category	Adj pval	Num in subset	Num total	Term	Ontology
GO:0003924	0.0017	6	86	GTPase activity	MF
GO:0005516	0.0000	6	12	calmodulin binding	MF
GO:0005525	0.0130	6	128	GTP binding	MF
GO:0031514	0.0000	6	28	motile cilium	CC
GO:0043234	0.0000	6	36	protein complex	CC
GO:0051258	0.0000	6	35	protein polymerization	BP

Enrichment of GO/Kegg categories; over represented in module 15

Category	Adj pval	Num in subset	Num total	Term	Ontology
GO:0003924	0.0005	6	86	GTPase activity	MF
GO:0005525	0.0038	6	128	GTP binding	MF
GO:0043234	0.0000	6	36	protein complex	CC
GO:0051258	0.0000	6	35	protein polymerization	BP
GO:0051287	0.0040	3	13	NAD binding	MF
path:lma00010	0.0381	4	39	Glycolysis / Gluconeogenesis	Metabolism; Carbohydrate metabolism
path:lma04145	0.0019	6	61	Phagosome	Cellular Processes; Transport and catabolism

Enrichment of GO categories; over represented in module 16

Category	Adj pval	Num in subset	Num total	Term	Ontology
GO:0000275	0.0468	2	4	mitochondrial proton-transporting ATP synthase complex, catalytic core F(1)	CC
GO:0004252	0.0468	2	5	serine-type endopeptidase activity	MF
GO:0015991	0.0468	3	23	ATP hydrolysis coupled proton transport	BP
GO:0046034	0.0468	2	4	ATP metabolic process	BP
GO:0070012	0.0298	2	2	oligopeptidase activity	MF

Enrichment of GO categories; over represented in module 17

Category	Adj pval	Num in subset	Num total	Term	Ontology
GO:0002036	0.0001	3	3	regulation of L-glutamate transport	BP
GO:0005353	0.0001	3	3	fructose transmembrane transporter activity	MF
GO:0005354	0.0001	3	3	galactose transmembrane transporter activity	MF
GO:0005886	0.0007	4	15	plasma membrane	CC
GO:0006971	0.0001	3	4	hypotonic response	BP
GO:0009190	0.0014	4	12	cyclic nucleotide biosynthetic process	BP
GO:0015193	0.0001	3	3	L-proline transmembrane transporter activity	MF
GO:0015238	0.0014	3	7	drug transmembrane transporter activity	MF
GO:0015578	0.0001	3	3	mannose transmembrane transporter activity	MF
GO:0015591	0.0045	2	2	D-ribose transmembrane transporter activity	MF
GO:0015752	0.0045	2	2	D-ribose transport	BP
GO:0015758	0.0001	3	3	glucose transport	BP
GO:0015893	0.0001	3	3	drug transport	BP
GO:0016849	0.0014	4	12	phosphorus-oxygen lyase activity	MF
GO:0022857	0.0012	3	6	transmembrane transporter activity	MF
GO:0022858	0.0001	3	3	alanine transmembrane transporter activity	MF
GO:0035524	0.0001	3	3	proline transmembrane transport	BP
GO:0035556	0.0045	4	20	intracellular signal transduction	BP
GO:0051955	0.0001	3	3	regulation of amino acid transport	BP
GO:0055056	0.0045	2	2	D-glucose transmembrane transporter activity	MF
GO:0070258	0.0045	2	2	inner membrane complex	CC
GO:0070881	0.0001	3	3	regulation of proline transport	BP
GO:0080144	0.0001	3	3	amino acid homeostasis	BP

Enrichment of GO categories; over represented in module 24

Category	Adj pval	Num in subset	Num total	Term	Ontology
GO:0000786	0.0000	7	29	nucleosome	CC
GO:0003677	0.0236	7	161	DNA binding	MF
GO:0005634	0.0057	7	149	nucleus	CC
GO:0006334	0.0000	7	33	nucleosome assembly	BP
GO:0043234	0.0030	4	36	protein complex	CC
GO:0046982	0.0000	7	31	protein heterodimerization activity	MF
GO:0051258	0.0030	4	35	protein polymerization	BP

Enrichment of GO/Kegg categories; over represented in module 29

Category	Adj pval	Num in subset	Num total	Term	Ontology
GO:0003735	0	13	166	structural constituent of ribosome	MF
GO:0005622	0	12	299	intracellular	CC
GO:0005840	0	13	157	ribosome	CC
GO:0006412	0	11	171	translation	BP
path:lma03010	0	13	169	Ribosome	Genetic Information Processing; Translation

Enrichment of GO/Kegg categories; over represented in module 30

Category	Adj pval	Num in subset	Num total	Term	Ontology
GO:0003746	0.037	3	15	translation elongation factor activity	MF
GO:0003924	0.037	5	86	GTPase activity	MF
GO:0005525	0.037	6	128	GTP binding	MF

Enrichment of GO/Kegg categories; over represented in module 32

Category	Adj pval	Num in subset	Num total	Term	Ontology
GO:0003735	0.0000	19	166	structural constituent of ribosome	MF
GO:0005622	0.0000	17	299	intracellular	CC
GO:0005840	0.0000	17	157	ribosome	CC
GO:0006412	0.0000	18	171	translation	BP
GO:0015934	0.0051	3	10	large ribosomal subunit	CC
path:lma03010	0	22	169	Ribosome	Genetic Information Processing; Translation

Enrichment of Kegg categories; over represented in module 39

Category	Adj pval	Num in subset	Num total	Term	Ontology
path:lma03013	0.0329	5	49	RNA transport	Genetic Information Processing; Translation

Enrichment of Kegg categories; over represented in module 40

Category	Adj pval	Num in subset	Num total	Term	Ontology
GO:0006950	0.0030	3	21	response to stress	BP
GO:0008234	0.0014	3	13	cysteine-type peptidase activity	MF
GO:0016209	0.0002	3	8	antioxidant activity	MF
GO:0051082	0.0388	3	53	unfolded protein binding	MF
GO:0051920	0.0002	3	8	peroxiredoxin activity	MF

Enrichment of GO/Kegg categories; over represented in module 65

Category	Adj pval	Num in subset	Num total	Term	Ontology
GO:0022891	0.0088	2	2	substrate-specific transmembrane transporter activity	MF

Enrichment of GO/Kegg categories; over represented in module 69

Category	Adj pval	Num in subset	Num total	Term	Ontology
GO:0003735	0e+00	19	166	structural constituent of ribosome	MF
GO:0005622	0e+00	15	299	intracellular	CC
GO:0005840	0e+00	19	157	ribosome	CC
GO:0006412	0e+00	19	171	translation	BP
GO:0015935	2e-04	5	16	small ribosomal subunit	CC
path:lma03010	0	24	169	Ribosome	Genetic Information Processing; Translation

Enrichment of Kegg categories; over represented in module 70

Category	Adj pval	Num in subset	Num total	Term	Ontology
path:lma03010	0.0446	4	169	Ribosome	Genetic Information Processing; Translation

Enrichment of GO/Kegg categories; over represented in module 98

Category	Adj pval	Num in subset	Num total	Term	Ontology
GO:0004812	0.0118	3	24	aminoacyl-tRNA ligase activity	MF
GO:0006418	0.0118	3	24	tRNA aminoacylation for protein translation	BP
path:lma00970	0.0112	3	28	Aminoacyl-tRNA biosynthesis	Genetic Information Processing; Translation

Enrichment of GO/Kegg categories; over represented in module 110

Category	Adj pval	Num in subset	Num total	Term	Ontology
GO:0004478	0.0097	2	2	methionine adenosyltransferase activity	MF
GO:0006556	0.0097	2	2	S-adenosylmethionine biosynthetic process	BP
GO:0015948	0.0097	2	2	methanogenesis	BP

Enrichment of GO/Kegg categories; over represented in module 113

Category	Adj pval	Num in subset	Num total	Term	Ontology
GO:0006412	0.0327	13	171	translation	BP
path:lma03010	1e-04	17	169	Ribosome	Genetic Information Processing; Translation

Enrichment of GO/Kegg categories; over represented in module120

Category	Adj pval	Num in subset	Num total	Term	Ontology
path:lma03010	0.0072	7	169	Ribosome	Genetic Information Processing; Translation

Enrichment of GO/Kegg categories; over represented in module132

Category	Adj pval	Num in subset	Num total	Term	Ontology
GO:0044267	0.0309	3	12	cellular protein metabolic process	BP

Enrichment of GO/Kegg categories; over represented in module143

Category	Adj pval	Num in subset	Num total	Term	Ontology
GO:0016593	0.0495	2	2	Cdc73/Paf1 complex	CC
GO:0032968	0.0495	2	2	positive regulation of transcription elongation from RNA polymerase II promoter	BP

Enrichment of GO/Kegg categories; over represented in module 148

Category	Adj pval	Num in subset	Num total	Term	Ontology
GO:0003735	0	19	180	structural constituent of ribosome	MF
GO:0005622	0	19	377	intracellular	CC
GO:0005840	0	18	173	ribosome	CC
GO:0006412	0	19	224	translation	BP
path:lma03010	0	21	169	Ribosome	Genetic Information Processing; Translation

Enrichment of GO/Kegg categories; over represented in module 149

Category	Adj pval	Num in subset	Num total	Term	Ontology
GO:0030286	0.0054	7	15	dynein complex	CC

Enrichment of GO/Kegg categories; over represented in module 150

Category	Adj pval	Num in subset	Num total	Term	Ontology
GO:0003735	0	16	180	structural constituent of ribosome	MF
GO:0005622	0	16	377	intracellular	CC
GO:0005840	0	16	173	ribosome	CC
GO:0006412	0	15	224	translation	BP
path:lma03010	0	16	169	Ribosome	Genetic Information Processing; Translation

Enrichment of GO/Kegg categories; over represented in module 172

Category	Adj pval	Num in subset	Num total	Term	Ontology
GO:0004197	0.0250	2	7	cysteine-type endopeptidase activity	MF
GO:0004519	0.0009	3	15	endonuclease activity	MF
GO:0006308	0.0004	3	9	DNA catabolic process	BP

Enrichment of GO/Kegg categories; over represented in module 207

Category	Adj pval	Num in subset	Num total	Term	Ontology
GO:0003735	0.0000	8	166	structural constituent of ribosome	MF
GO:0005622	0.0299	6	299	intracellular	CC
GO:0005840	0.0000	9	157	ribosome	CC
GO:0006412	0.0000	8	171	translation	BP
GO:0032465	0.0073	2	3	regulation of cytokinesis	BP
path:lma03010	3e-04	8	169	Ribosome	Genetic Information Processing; Translation
