# Supplementary material for: The Transcriptome of Leishmania major Developmental Stages in Their Natural Sand Fly Vector
Source: mBio. 2017 Apr 4;8(2):e00029-17. doi: 10.1128/mBio.00029-17 (PMC5380837; doi:10.1128/mBio.00029-17)
Supplement: TEXT S1 [file mbo002173254s1.pdf]

Supplemental file: sandfly co-expression cluster analysis

V. Keith Hughitt

12 December, 2016

Contents

Overview 1
Supplemental Figures and Tables 1
Data Preparation 44
Reproducing this analysis 45
References 46
System Information 47

Overview

This document contains all of the code used for the co-expression cluster analysis portion of the manuscript, including all relevant figures and tables from the manuscript.

The analyses in this document start with raw RNA-Seq count tables. For information on how the original RNA-Seq samples were processed into count tables, see the section title "Data Preparation" below.

In order to ensure that all results described here can be eaisly reproduced, this document and all figures and tables herein have been automatically generated. For instructions on how to re-run these analyses, see the sectioned titled "Reproducing this analysis".

Supplemental Figures and Tables

```
library('knitcitations')

# hide information about knit progress from output
opts_knit$set(
    progress=FALSE,
    verbose=FALSE,
    message=FALSE,
    warning=FALSE,
    error=FALSE
)

# If rmarkdown.pandoc.to not specified (for example, when kniting
# piece-by-piece in Vim-R), have it default to 'latex' output.
if (is.null(opts_knit$get("rmarkdown.pandoc.to"))) {
    opts_knit$set(rmarkdown.pandoc.to='latex')
}

# For images containing unicode text, we will use cairo for PDF output
opts_chunk$set(dev='cairo_pdf',
               dev.args=list(cairo_pdf=list(family="DejaVu Sans")))

# Other options
```

```r
options(digits=4)
options(stringsAsFactors=FALSE)
options(knitr.duplicate.label='allow')
options(citation_format='pandoc')

# Clean up any existing variables
rm(list=ls())

# Make sure results are reproducible
set.seed(1)
```

```r
# Load required libraries
library('Biobase')
library('biomaRt')
library('doParallel')
library('flashClust')
library('foreach')
library('ggplot2')
library('goseq')
library('GO.db')
library('gplots')
library('limma')
library('Matrix')
library('dplyr')
library('readr')
library('tools')
library('rtracklayer')
library('preprocessCore')
library('reshape2')
library('RColorBrewer')
library('RCurl')
library('hpgltools')
library('gridExtra')
library('WGCNA')

# Enable WGCNA multithreading
allowWGCNAThreads()
enableWGCNAThreads()

# Load helper functions
# https://github.com/elsayed-lab/manuscript-shared-rnaseq
source('../R/annotations.R')
source('../R/count_tables.R')
source('../R/enrichment_analysis.R')
source('../R/filtering.R')
source('../R/plots.R')
source('../R/wgcna.R')

# Side-by-side plots (PDF)
combined_plot_width = '3.5in'
```

```r
samples <- tbl_df(read.csv('samples.csv')) %>%
    filter(parasite == 'L. major Ryan' & condition != "Culture metac")
    #filter(parasite == 'L. major Ryan')
```

```r
# fix repeat column name; repeat is conserved word in R and using as column
# name causes problems with dplyr; rename to replicate
colnames(samples)[6] <- 'replicate'

# fix condition names (spaces aren't allowed in makeContrasts)
samples$condition <- gsub(' ', '_', samples$condition)

# input directory
parts <- unlist(strsplit(getwd(), '/'))
input_dir <- do.call(file.path, as.list(c(parts[1:length(parts) - 1], 'input')))

# DE comparisons
condition_pairs <- t(combn(unique(samples$condition), 2))
de_comparisons <- list()
for (i in 1:nrow(condition_pairs)) {
    de_comparisons[[i]] <- as.character(condition_pairs[i,])
}

CONFIG <- list(
    # General
    'pathogen'            = 'L. major',
    'host'                = 'P. duboscqi',
    'target'              = 'pathogen',
    'analysis_name'       = 'lmajor-ryan-intracellular-v5.3',
    'use_cache'           = TRUE,
    'verbose'             = FALSE,
    'debug'               = FALSE,
    'save_results'        = TRUE,
    'include_tables'      = TRUE,

    # Sample variables of interest
    'sample_id'           = 'sample_id',
    'condition'           = 'condition',
    'batch'               = 'replicate',

    # Covariates
    'covariates'          = c('replicate'),

    # Plotting
    'fig_width'           = 1080,
    'fig_height'          = 1080,
    'dpi'                 = 96,

    # Input count tables
    "input_count_tables"  = file.path("/cbcb/nelsayed-scratch/dillonl/sacks/count_tables",
                                  "20150611_Lmajor60_6961-72_6974_6976-77_6979-80_CDSonly.count"),

    # Gene filtering
    'low_count_threshold' = 1,
    'id_filter_string'    = 'rRNA|5SRRNA|snRNA|snoRNA|SLRNA|TRNA|SRP',
    'type_filter_string'  = 'rRNAencoding|snRNAencoding|snoRNAencoding|tRNAencoding',

    # Normalization / data transformation
```

```r
    'network_cpm'                    = TRUE,
    'network_log2'                   = TRUE,
    'network_voom'                   = FALSE,
    'network_quantile_normalize'     = FALSE,
    'network_ebayes_robust'          = TRUE,
    'network_batch_adjust'           = 'none',

    # Annotations
    "organism_db"          = "Leishmania.major.Friedlin",
    "organism_genome"      = "LmJF",
    "orgdb_key"            = "GID",

    # Output filepaths
    "output_dir"           = file.path(Sys.getenv('SCRATCH'), "coex_networks",
                                       "Lmajor_infecting_Pduboscqi"),

    # Network construction
    'network_type'         = 'signed',
    'similarity_measure'   = 'cor-dist',
    'adjmatrix_power'      = 4,
    'topological_overlap' = FALSE,

    # Module detection
    'cut_tree_method'         = 'tree',
    'min_module_size'         = 10,
    'deep_split'              = FALSE,

    # Samples to include
    "samples" = samples,
    "wgcna_samples"  = samples,

    # Differential expression contrasts
    "de_comparisons"    = de_comparisons,
    "de_max_pvalue"     = 0.05,
    "de_min_log2fc"     = 0,

    # Alternate condition values to use for plotting, etc.
    "condition_mapping" = data.frame(
        long=unique(samples$condition),
        short=c("AM", 'PP', 'NP', 'MP')
    )
)
```

```r
# set figure dimensions and dpi
opts_chunk$set(fig.width=CONFIG$fig_width/CONFIG$dpi,
               fig.height=CONFIG$fig_height/CONFIG$dpi,
               fig.retina=1,
               dpi=CONFIG$dpi)
```

```r
# Samples included in this analysis
if (CONFIG$include_tables) {
    kable(CONFIG$samples, caption='RNA-Seq samples.')
}
```

Table 1: RNA-Seq samples.

sample_id	parasite	host	condition	day	replicate
6961	L. major Ryan	M. musculus	Footpad_amast	NA	L
6962	L. major Ryan	P. duboscqi	Sandfly_day_2	2	L
6963	L. major Ryan	P. duboscqi	Sandfly_day_4	4	L
6964	L. major Ryan	P. duboscqi	Sandfly_day_15	15	L
6966	L. major Ryan	M. musculus	Footpad_amast	NA	M
6967	L. major Ryan	P. duboscqi	Sandfly_day_2	2	M
6968	L. major Ryan	P. duboscqi	Sandfly_day_4	4	M
6969	L. major Ryan	P. duboscqi	Sandfly_day_15	15	M

```
# Sample metadata
sample_ids <- as.character(CONFIG$samples[[CONFIG$sample_id]])
condition  <- factor(CONFIG$samples[[CONFIG$condition]])
batch      <- factor(CONFIG$samples[[CONFIG$batch]])

# Covariate dataframe
covariates <- CONFIG$samples %>% select_(.dots=CONFIG$covariates)

# Design matrix; this may be updated later if batch adjustment is enabled
design_condition_only  <- model.matrix(~0+condition)
design_including_batch <- model.matrix(~0+condition+batch)
```

```
message("Preparing count matrix")

count_table <- read.table(CONFIG$input_count_tables, header=TRUE,
                          row.names=1, check.names=FALSE)

# Filter out samples not needed for this analysis
count_table <- count_table[,sample_ids]

# Drop any rows with NA's (should only occur when comparing counts across
# experiments where different reference GFF's were used)
count_table <- count_table[complete.cases(count_table),]

# Fix gene identifiers
rownames(count_table) <- sub('-1', '', rownames(count_table))

# Genes with alternative transcripts defined (86 total for L. major Friedlin)
alt_txids <- rownames(count_table)[grepl('-[0-9]$', rownames(count_table))]

# Collapse multi-exon gene CDSs, e.g. "LmjF.02.0100-2" "LmjF.02.0100-3"...
multi_exon_counts <- count_table[rownames(count_table) %in% alt_txids,]

# Remove non-primary CDSs from count table
count_table <- count_table[!rownames(count_table) %in% alt_txids,]

# Add counts from other CDSs for gene
multi_exon_genes <- unique(sub('-[2-9]', '', alt_txids))
for (x in multi_exon_genes) {
    counts <- colSums(multi_exon_counts[grepl(x, alt_txids),])
    count_table[x,] = count_table[x,] + counts
```

```
}
```

```r
# Load gene annotations
library(CONFIG$organism_db, character.only=TRUE)
orgdb <- get(CONFIG$organism_db)

# Fix AnnotationDbi namespace mess
assign('select', dplyr::select, envir=.GlobalEnv)
assign('get',    base::get, envir=.GlobalEnv)

gene_info <- load_parasite_annotations(orgdb, rownames(count_table),
                                       keytype=CONFIG$orgdb_key)

# Get transcript lengths (sum of all exon lengths for each gene)
txdb <- orgdb@txdbSlot
transcript_lengths <- transcriptLengths(txdb)
transcript_lengths <- transcript_lengths[transcript_lengths$gene_id %in%
                                         gene_info$gene_id,]

gene_info[match(transcript_lengths$gene_id, gene_info$gene_id),
            'transcript_length'] <- transcript_lengths$tx_len
gene_info$transcript_length <- as.numeric(gene_info$transcript_length)

# 2015/06/22 Add placeholder entry for LmjF.28.2965;
# currently excluded from annotation database because of potentially
# problematic annotation (strange exon structure). Contracted TriTrypDB
# regarding the gene.
gene_info <- rbind(gene_info,
                    data.frame(gene_id='LmjF.28.2965',
                                chromosome='LmjF.28',
                                description=NA, strand=NA, type=NA,
                                transcript_length=NA))

# Keep only the feature information remaining genes
gene_info <- gene_info[gene_info$gene_id %in% rownames(count_table),]

# For now, just grab the description for the first transcript
gene_info <- gene_info[!duplicated(gene_info$gene_id),]

# Gene IDs
gene_ids <- rownames(count_table)
```

```r
# Load GO annotations
go_terms <- load_go_terms(orgdb, rownames(count_table),
                          keytype=CONFIG$orgdb_key)

# Gene / GO term mapping
gene_go_mapping <- as.data.frame(unique(
    go_terms %>% select(get(CONFIG$orgdb_key), GO, ONTOLOGY)
))
colnames(gene_go_mapping) <- c('gene', 'category', 'ontology')

# Mapping from GO ID to term and ontology
go_term_id_mapping <- as.data.frame(unique(go_terms[c('GO', 'TERM', 'ONTOLOGY')]))
```

```r
colnames(go_term_id_mapping) <- c("category", "term", "ontology")
```

```r
# L. major, T. cruzi, etc
gene_kegg_mapping <- load_kegg_mapping(orgdb, rownames(count_table),
                                        keytype=CONFIG$orgdb_key)
kegg_pathways <- load_kegg_pathways(orgdb, rownames(count_table),
                                    keytype=CONFIG$orgdb_key)

# Rename gene/KEGG mapping columns to be consistent with GO mapping
colnames(gene_kegg_mapping) <- c('gene', 'category')
colnames(kegg_pathways)     <- c('category', 'name', 'class', 'description')

kegg_pathways <- unique(kegg_pathways)
```

```r
# Assay data (RNA-Seq count table)
assay_data <- as.matrix(count_table)

# Sort rows by gene id
assay_data <- assay_data[order(row.names(assay_data)),]

# Pheno data (sample information)
pheno_data <- new("AnnotatedDataFrame",
                  data.frame(sample=sample_ids, condition=condition, batch=batch))
sampleNames(pheno_data) <- colnames(assay_data)

# Feature data (gene annotations)
gene_info <- gene_info[order(gene_info$gene_id),]
feature_data <- new("AnnotatedDataFrame", as.data.frame(gene_info))

#featureNames(feature_data) <- rownames(assay_data)
featureNames(feature_data) <- gene_info$gene_id

# List to store counts used for co-expression analysis
network_counts = list()

network_counts$raw <- new("ExpressionSet", exprs=assay_data,
                          phenoData=pheno_data, featureData=feature_data)
```

```r
reps_per_batch <- table(factor(condition))
min_replicates <- min(reps_per_batch)
network_counts$raw <- filter_lowcount_genes(network_counts$raw,
                                            threshold=CONFIG$low_count_threshold,
                                            min_samples=min_replicates,
                                            verbose=FALSE)
```

```r
# Filter by gene id or type
ncrna_mask <- rep(FALSE, nrow(network_counts$raw))

if (!is.null(CONFIG$id_filter_string)) {
    ncrna_mask <- ncrna_mask | grepl(CONFIG$id_filter_string,
                                     rownames(network_counts$raw))
}

if (!is.null(CONFIG$type_filter_string)) {
```

```r
    gene_types <- (gene_info %>% filter(gene_id %in% rownames(network_counts$raw)))$type
    ncrna_mask <- ncrna_mask | grepl(CONFIG$type_filter_string, gene_types)
}
network_counts$raw <- network_counts$raw[!ncrna_mask,]
```

```r
# Keep annotations for remaining genes only
gene_info <- gene_info[gene_info$gene_id %in% rownames(network_counts$raw),]
```

```r
# Create copies of raw count ExpressionSet
network_counts$log2cpm <- network_counts$raw

# Keep a copy of the Log2-CPM normalized counts for plotting purposes
exprs(network_counts$log2cpm) <- log2(counts_per_million(exprs(network_counts$raw)) + 0.5)
```

```r
# Differential expression parameters
counts              <- network_counts
batch_adjust        <- CONFIG$network_batch_adjust
quantile_normalize  <- CONFIG$network_quantile_normalize
use_cpm             <- CONFIG$network_cpm
use_log2            <- CONFIG$network_log2
use_voom            <- CONFIG$network_voom
robust_ebayes       <- CONFIG$network_ebayes_robust
```

```r
# Apply voom/quantile normalization on counts
counts$normed <- counts$raw

# voom (includes log2-CPM transformation)
if (use_voom) {
    # quantile normalize?
    norm_method <- ifelse(quantile_normalize, 'quantile', 'none')

    # voom data
    voom_result <- voom(exprs(counts$raw), design_condition_only,
                        normalize.method=norm_method,
                        plot=FALSE)

    # create normed_counts
    exprs(counts$normed) <- voom_result$E
} else {
    # Based on a suggestion from HCB, we will apply quantile normalization
    # prior to logging when CPM is not used. This is in contrast to voom
    # which applies qnorm after log-CPM transforming the data.
    gene_ids <- rownames(counts$normed)

    # Counts-per-million
    if (use_cpm) {
        exprs(counts$normed) <- counts_per_million(exprs(counts$raw))

        # Log2 transformation
        if (use_log2) {
            exprs(counts$normed) <- log2(exprs(counts$normed) + 0.5)
        }

        # Quantile normalize?
```

```
        if (quantile_normalize) {
            exprs(counts$normed) <- normalize.quantiles(exprs(counts$normed))
        }
    } else {
        # Quantile normalize?
        if (quantile_normalize) {
            exprs(counts$normed) <- normalize.quantiles(exprs(counts$normed))
        }

        # Log2 transformation
        if (use_log2) {
            exprs(counts$normed) <- log2(exprs(counts$normed) + 0.5)
        }
    }
    rownames(counts$normed) <- gene_ids
    colnames(counts$normed) <- sample_ids
}

counts$final <- counts$normed
```

```
# Store transformed network counts
network_counts <- counts

# Update condition, batch, etc. to include only network-specific samples
condition <- factor(CONFIG$wgcna_samples[[CONFIG$condition]])
batch     <- factor(CONFIG$wgcna_samples[[CONFIG$batch]])

# Samples to use for network construction
sample_ids_network  <- as.character(CONFIG$wgcna_samples[[CONFIG$sample_id]])
network_sample_mask <- sampleNames(network_counts$raw) %in% sample_ids_network

covariates <- covariates[network_sample_mask,]

# drop samples not used in network construction
network_counts$final            <- network_counts$final[,network_sample_mask]
```

```
# Update gene annotations
gene_info <- gene_info[gene_info$gene_id %in% rownames(network_counts$final),]

# Update rows and columns for each version of network_counts to only
# include those genes that remain in network_counts$final
gene_ids <- rownames(network_counts$final)

for (name in names(network_counts)) {
    # Update network count tables
    if (ncol(network_counts[[name]]) == length(network_sample_mask)) {
        sample_ind <- network_sample_mask
    } else {
        sample_ind <- rep(TRUE, ncol(network_counts[[name]]))
    }
    network_counts[[name]] <- network_counts[[name]][rownames(network_counts[[name]]) %in% gene_ids, sa
}
```

```r
# WGCNA count input
wgcna_input <- exprs(network_counts$final)

# Convert to float (necessary for cor)
wgcna_input <- wgcna_input * 1.0

# Combination of Pearson correlation and Euclidean distance
cor_matrix  <- cor(t(wgcna_input))
dist_matrix <- as.matrix(dist(wgcna_input, diag=TRUE, upper=TRUE))
dist_matrix <- log1p(dist_matrix)
dist_matrix <- 1 - (dist_matrix / max(dist_matrix))

similarity_matrix <- sign(cor_matrix) * ((abs(cor_matrix) + dist_matrix)/ 2)

# clean-up
rm(cor_matrix)
rm(dist_matrix)
gc()
```

```r
# Set the diagonal to zero to remove uninformative correlations
diag(similarity_matrix) <- 0

# Construct adjacency matrix
adjacency_matrix <- adjacency.fromSimilarity(similarity_matrix,
                                             power=CONFIG$adjmatrix_power,
                                             type=CONFIG$network_type)

# Delete similarity matrix to free up memory
rm(similarity_matrix)
gc()

# Convert to matrix
adjacency_matrix <- matrix(adjacency_matrix, nrow=nrow(adjacency_matrix))
rownames(adjacency_matrix) <- gene_ids
colnames(adjacency_matrix) <- gene_ids
```

```r
# Dissimilarity matrix (used for clustering distance metric)
dissimilarity_matrix <- 1 - adjacency_matrix

# Delete adjacency matrix for now to free up memory
rm(adjacency_matrix)
gc()
```

```r
# Create a transposed version of the count table
sample_counts <- as.data.frame(t(wgcna_input))

# Cluster gene expression profiles
gene_tree <- flashClust(as.dist(dissimilarity_matrix), method="average")

# 2015/11/05: Work-around for cutree bug:
# "the 'height' component of 'tree' is not sorted (increasingly)"
# See: https://stat.ethz.ch/pipermail/r-help/2008-May/163409.html
gene_tree$height <- round(gene_tree$height, 6)
```

```r
module_labels <- cutreeDynamicTree(dendro=gene_tree,
                                   deepSplit=CONFIG$deep_split,
                                   minModuleSize=CONFIG$min_module_size)
module_colors <- labels2colors(module_labels)
```

```r
num_genes   <- ncol(sample_counts)
num_samples <- nrow(sample_counts)

# Calculate eigengenes
module_eigengenes <- moduleEigengenes(sample_counts,
                                      colors=module_colors)$eigengenes

# total number of modules remaining after merge
num_modules <- length(unique(module_colors))
```

```r
# Create result data frame
result <- cbind(gene_info, color=module_colors)

# drop unneeded columns
keep_cols <- intersect(c('gene_id', 'color', 'description', 'chromosome',
                         'strand', 'transcript_length'), colnames(result))
result <- tbl_df(result[,keep_cols])

# add expression-related fields
result$expr_variance <- apply(wgcna_input, 1, var)
result$expr_mean     <- apply(wgcna_input, 1, mean)

# Recreate adjacency matrix and free up space from dissimilarity matrix
adjacency_matrix <- 1 - dissimilarity_matrix
rm(dissimilarity_matrix)
gc()

COEXPRESSION_NETWORK_RESULT <- list(
    adj_matrix=adjacency_matrix,
    wgcna_input=wgcna_input,
    gene_tree=gene_tree,
    gene_info=gene_info,
    module_colors=module_colors,
    module_eigengenes=module_eigengenes,
    result=result
)
```

```r
# replace color names with module numbers; modules of interest are first
# assigned lower numbers and remaining modules assigned arbitrary numbers
modules_of_interest <- c('turquoise', 'pink', 'cornflowerblue', 'lightskyblue3',
                         'brown', 'skyblue3', 'lightblue1', 'purple',
                         'antiquewhite4', 'firebrick2', 'indianred4',
                         'lightpink4', 'green2', 'blueviolet', 'salmon1',
                         'indianred2', 'orangered4', 'palevioletred1',
                         'skyblue4', 'pink1', 'pink2', 'tomato4',
                         'lightcyan', 'thistle1', 'saddlebrown', 'honeydew1',
                         'darkslateblue', 'blue', 'wheat3', 'magenta3', 'tan4',
                         'darkseagreen2', 'darkviolet', 'paleturquoise',
                         'grey', 'orange3', 'skyblue1', 'sienna3',
```

```
                         'greenyellow', 'lavenderblush', 'lightpink1',
                         'antiquewhite1', 'brown4', 'midnightblue',
                         'darkolivegreen1')
remaining_modules <- unique(module_colors)[!unique(module_colors) %in%
                                           modules_of_interest]
module_label_mapping <- rbind(cbind(modules_of_interest,
                                    seq_along(modules_of_interest)),
                              cbind(remaining_modules,
                                    seq_along(remaining_modules) +
                                    length(modules_of_interest)))

module_label_mapping <- as.data.frame(module_label_mapping)
colnames(module_label_mapping) <- c('color', 'number')

# replace colors with numbers
module_colors <- module_label_mapping$number[match(module_colors,
                                                   module_label_mapping$color)]
result$color <- module_label_mapping$number[match(result$color,
                                                  module_label_mapping$color)]

# create output directory
timestring <- format(Sys.time(), "%Y%m%d")

# output directory
output_dir <- file.path(CONFIG$output_dir,
                        sprintf("%s_%s", CONFIG$analysis_name, timestring))
outdir <- file.path(output_dir, 'output')

# Directory to store module enrichment results to
module_output_dir <- file.path(outdir, 'modules')
if (!dir.exists(module_output_dir)) {
    dir.create(module_output_dir, recursive=TRUE)
}

# Data frame of module sizes
module_counts <- c()
for (color in unique(module_colors)) {
    module_counts <- append(module_counts, sum(module_colors == color))
}

# create a mapping from module id to number of genes for later use
module_sizes <- data.frame(
    module_id=unique(module_colors),
    num_genes=module_counts
)
```

Co-expression modules

```
# To get a sense for how the genes were clustered, let's plot plot a subset of
# the expression profiles and highlight the genes in each module.
combined_counts <- combine_replicates(exprs(network_counts$final), condition)
combined_counts_table <- cbind(gene_id=rownames(network_counts$final),
```

```r
                                combined_counts)

# convert to long
counts_long <- melt_counts(combined_counts, CONFIG$condition_mapping)
colnames(counts_long) <- c('gene_id', 'condition', 'log_cpm')

# add clustering results and convert to a factor
counts_long <- cbind(counts_long, cluster=as.factor(module_colors))

# save result
COEXPRESSION_NETWORK_RESULT$counts_long <- counts_long

# load list of genes to highlight
genes_of_interest <- read_tsv('../../settings/genes_of_interest.tsv')
```

```
## Parsed with column specification:
## cols(
##   gene_id = col_character(),
##   description = col_character(),
##   group = col_character(),
##   subgroup = col_character()
## )
```

```r
# fix newlines
genes_of_interest$subgroup <- gsub('\\\\n', '\n', genes_of_interest$subgroup)

# set default line color and linetype
genes_of_interest$color <- '#B0B0B0'
genes_of_interest$linetype <- 'solid'

# Assign a separate color palette for each group and subgroup
gene_groups <- unique(genes_of_interest$group)

# list of linetypes to use when differentiating subgroups
linetypes <- rep(c("solid", "dashed", "longdash", "F1", "dotted", "dotdash",
                   "twodash", "1F", "4C88C488", "12345678"), 2)

# Palettes: Purple, Green, Red, Blue
palette_ranges <- list(rev(c('#FFAAD4', '#800007')), rev(c('#99FFFC', '#060042')),
                       rev(c('#8CAB8C', '#004200')), rev(c('#F2D69D', '#A86000')))

color_palettes <- lapply(palette_ranges, colorRampPalette, space='Lab')
names(color_palettes) <- gene_groups

# iterate over main gene groups
for (gene_group in gene_groups) {
    # get a palette
    genes <- genes_of_interest %>% filter(group == gene_group)
    num_subgroups <- length(unique(genes$subgroup))
    pal <- color_palettes[[gene_group]](num_subgroups)

    ltypes <- linetypes[1:num_subgroups]

    # iterate over gene sub-groups
```

```r
    gene_subgroups <- unique(genes$subgroup)

    for (i in seq_along(gene_subgroups)) {
        gene_subgroup <- gene_subgroups[i]
        genes_in_subgroup <- (genes %>% filter(subgroup == gene_subgroup))$gene_id
        genes_of_interest[genes_of_interest$gene_id %in% genes_in_subgroup,'color'] <- pal[i]
        genes_of_interest[genes_of_interest$gene_id %in% genes_in_subgroup,'linetype'] <- ltypes[i]
    }
}

# Subgroup color mapping
color_mapping <- unique(genes_of_interest %>% select(subgroup, color))
color_mapping <- rbind(color_mapping, c('Other', '#B0B0B0'))

# Linetype mapping
linetype_mapping <- unique(genes_of_interest %>% select(subgroup, linetype))
linetype_mapping <- rbind(linetype_mapping, c('Other', 'solid'))

# Add group information to long counts
counts_long <- merge(counts_long, genes_of_interest, by='gene_id', all.x=TRUE)
counts_long$subgroup[is.na(counts_long$subgroup)] <- "Other"

# work-around to ensure proper stacking order
# http://blog.mckuhn.de/2011/08/ggplot2-determining-order-in-which.html
counts_long$order <- ifelse(counts_long$subgroup == 'Other', 0, 1)
counts_long$gene_id <- factor(paste0(counts_long$order, counts_long$gene_id))

# Default color
counts_long$color[is.na(counts_long$color)] <- '#B0B0B0'
```

```r
# Number of modules to include in each plot
modules_per_plot <- 9

# Get a list of all modules, excluding 11 -- accidentally mislabeled in early
# versions of analysis; leaving in to avoid having to change rest of the
# module numbering
module_order <- module_label_mapping$number
#module_order <- module_order[module_order != 11]

num_modules <- length(module_order)

# Split modules into groups of n or less for plotting
module_groups <- split(module_order, ceiling(seq_along(module_order) /
                                              modules_per_plot))

for (i in names(module_groups)) {
    plots <- list()
    counter <- 1

    for (cluster_num in module_groups[[i]]) {
        # colors specified in variable
        cluster_subgroups <- unique((counts_long %>% filter(cluster == cluster_num))$subgroup)

        # get named vector representation of subgroup colors
```

```
        x <- color_mapping %>% filter(subgroup %in% cluster_subgroups)
        color_scale <- setNames(x$color, x$subgroup)

        # get named vector representation of subgroup linetypes
        x <- linetype_mapping %>% filter(subgroup %in% cluster_subgroups)
        linetype_scale <- setNames(x$linetype, x$subgroup)

        # let's also increase the size of the highlighted genes
        size_scale <- setNames(ifelse(x$subgroup=='Other', 0.2, 0.8), x$subgroup)

        # generate the plot
        plt <- module_profile_plot(counts_long, cluster_num,
                                   highlight_group='subgroup',
                                   scale_color_values=color_scale,
                                   scale_size_values=size_scale,
                                   scale_linetype_values=linetype_scale,
                                   font_size=9,
                                   xlabel='Developmental form')
        plots[[counter]] <- plt

        # save plot to disk
        outfile <- sprintf("%s/%s.png", module_output_dir, cluster_num)
        ggsave(filename=outfile, plot=plt, width=7, height=5, units='in', dpi=300)

        counter <- counter + 1
    }
    print(do.call("grid.arrange", c(plots, ncol=3)))
}
```

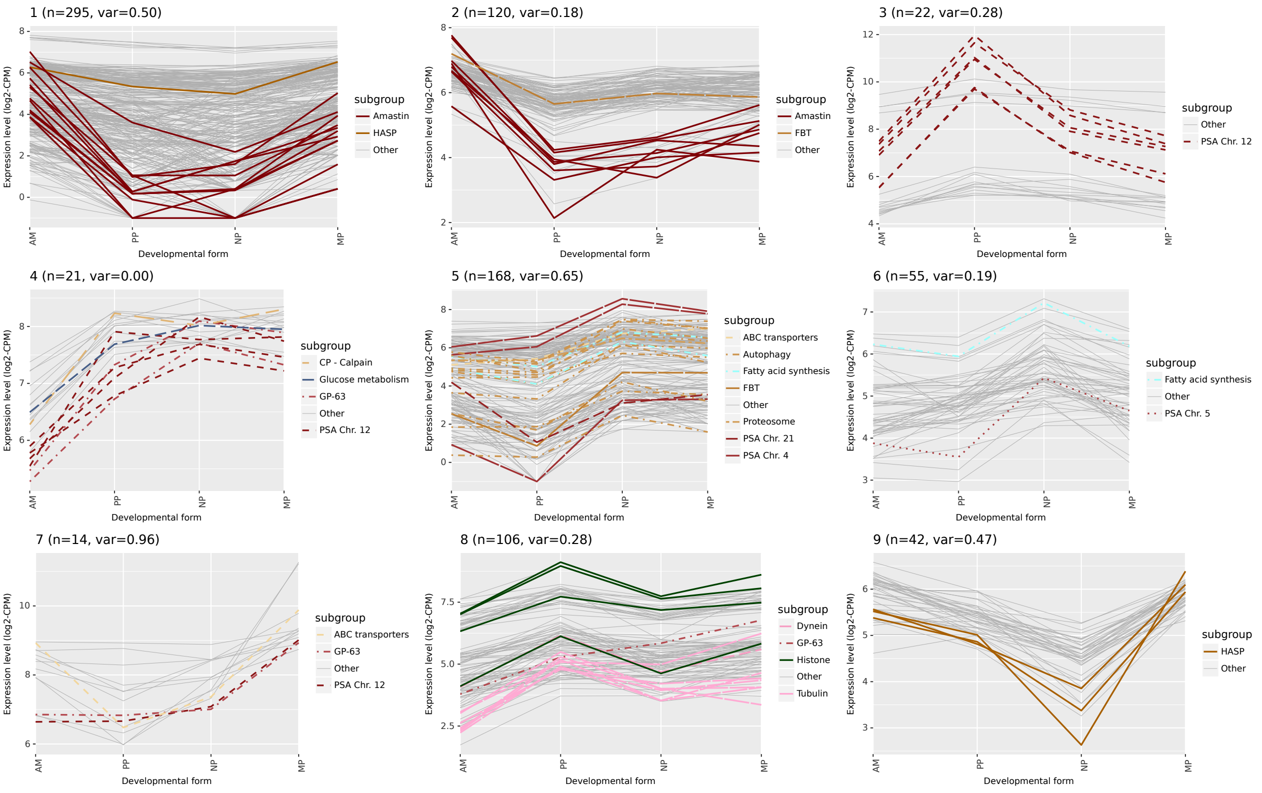

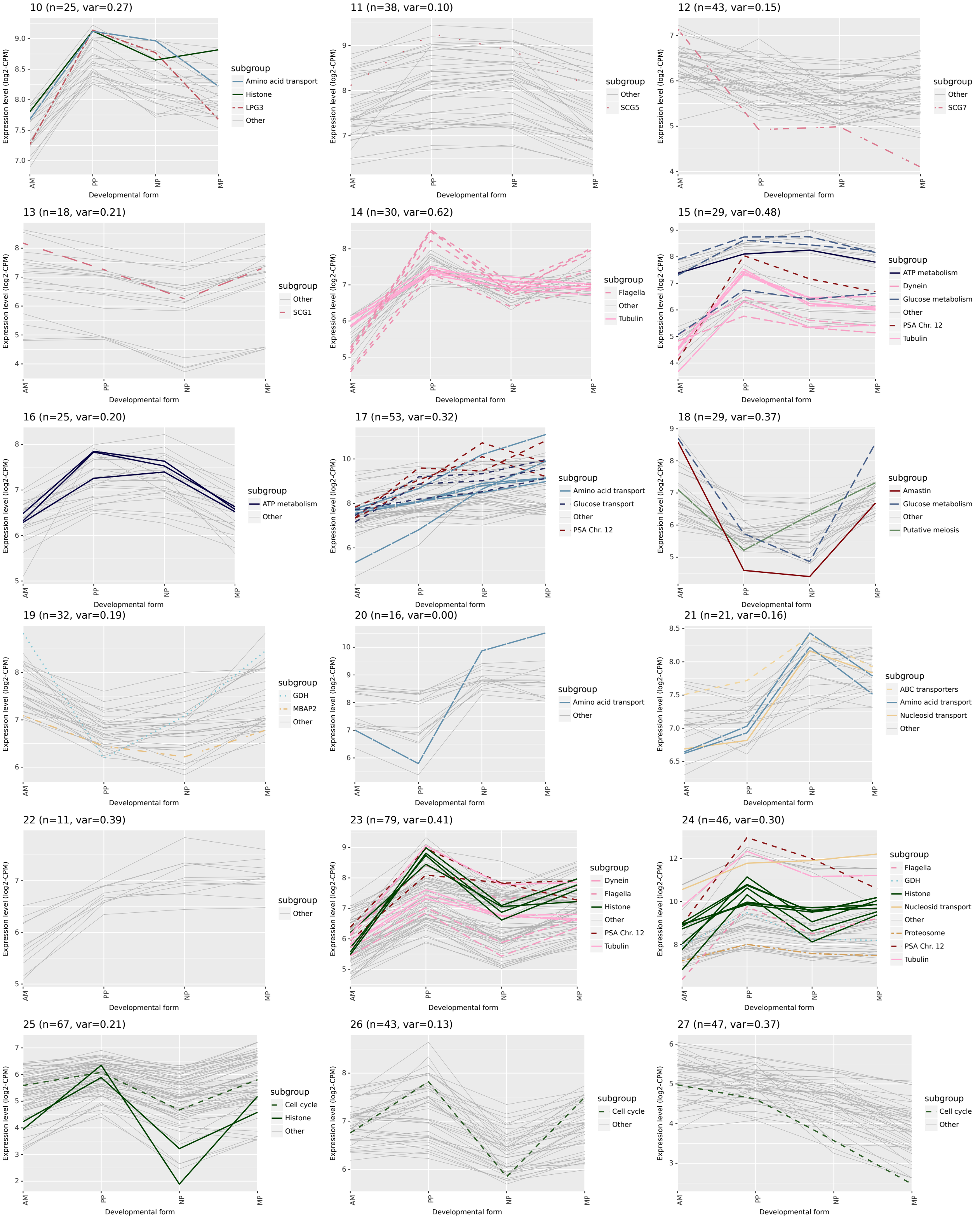
10 (n=25, var=0.27)
subgroup
Amino acid transport
Histone
LPG3
Other
11 (n=38, var=0.10)
subgroup
Other
SCG5
12 (n=43, var=0.15)
subgroup
Other
SCG7
13 (n=18, var=0.21)
subgroup
Other
SCG1
14 (n=30, var=0.62)
subgroup
Flagella
Other
Tubulin
15 (n=29, var=0.48)
subgroup
ATP metabolism
Dynein
Glucose metabolism
Other
PSA Chr. 12
Tubulin
16 (n=25, var=0.20)
subgroup
ATP metabolism
Other
17 (n=53, var=0.32)
subgroup
Amino acid transport
Glucose transport
Other
PSA Chr. 12
18 (n=29, var=0.37)
subgroup
Amastin
Glucose metabolism
Other
Putative meiosis
19 (n=32, var=0.19)
subgroup
GDH
MBAP2
Other
20 (n=16, var=0.00)
subgroup
Amino acid transport
Other
21 (n=21, var=0.16)
subgroup
ABC transporters
Amino acid transport
Nucleosid transport
Other
22 (n=11, var=0.39)
subgroup
Other
23 (n=79, var=0.41)
subgroup
Dynein
Flagella
Histone
Other
PSA Chr. 12
Tubulin
24 (n=46, var=0.30)
subgroup
Flagella
GDH
Histone
Nucleosid transport
Other
Proteosome
PSA Chr. 12
Tubulin
25 (n=67, var=0.21)
subgroup
Cell cycle
Histone
Other
26 (n=43, var=0.13)
subgroup
Cell cycle
Other
27 (n=47, var=0.37)
subgroup
Cell cycle
Other
Expression level (log2-CPM)
Developmental form
AM
PP
NP
MP

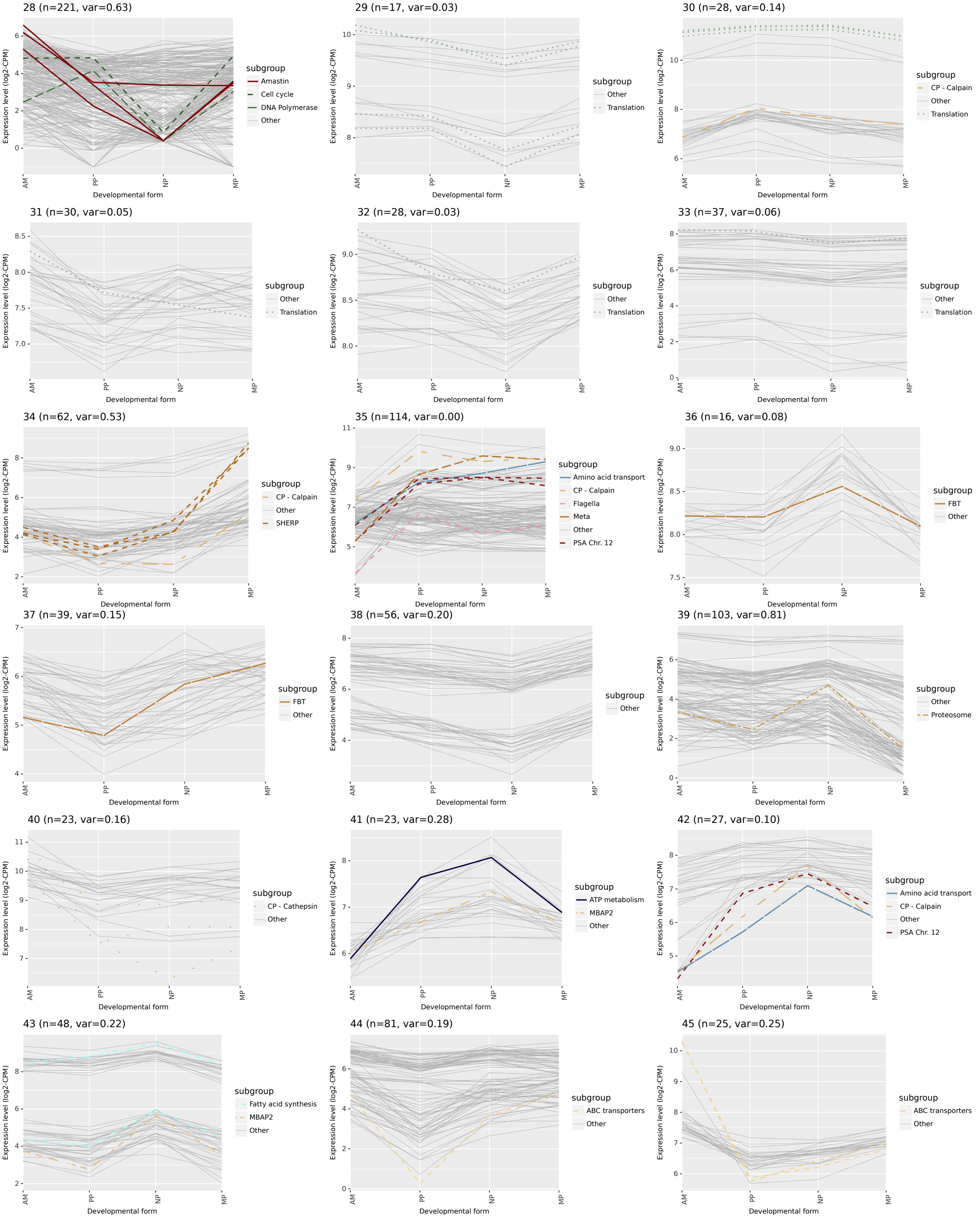

28 (n=221, var=0.63)
subgroup
Amastin
Cell cycle
DNA Polymerase
Other
29 (n=17, var=0.03)
subgroup
Other
Translation
30 (n=28, var=0.14)
subgroup
CP - Calpain
Other
Translation
31 (n=30, var=0.05)
subgroup
Other
Translation
32 (n=28, var=0.03)
subgroup
Other
Translation
33 (n=37, var=0.06)
subgroup
Other
Translation
34 (n=62, var=0.53)
subgroup
CP - Calpain
Other
SHERP
35 (n=114, var=0.00)
subgroup
Amino acid transport
CP - Calpain
Flagella
Meta
Other
PSA Chr. 12
36 (n=16, var=0.08)
subgroup
FBT
Other
37 (n=39, var=0.15)
subgroup
FBT
Other
38 (n=56, var=0.20)
subgroup
Other
39 (n=103, var=0.81)
subgroup
Other
Proteosome
40 (n=23, var=0.16)
subgroup
CP - Cathepsin
Other
41 (n=23, var=0.28)
subgroup
ATP metabolism
MBAP2
Other
42 (n=27, var=0.10)
subgroup
Amino acid transport
CP - Calpain
Other
PSA Chr. 12
43 (n=48, var=0.22)
subgroup
Fatty acid synthesis
MBAP2
Other
44 (n=81, var=0.19)
subgroup
ABC transporters
Other
45 (n=25, var=0.25)
subgroup
ABC transporters
Other
Expression level (log2-CPM)
Developmental form
AM
PP
NP
MP

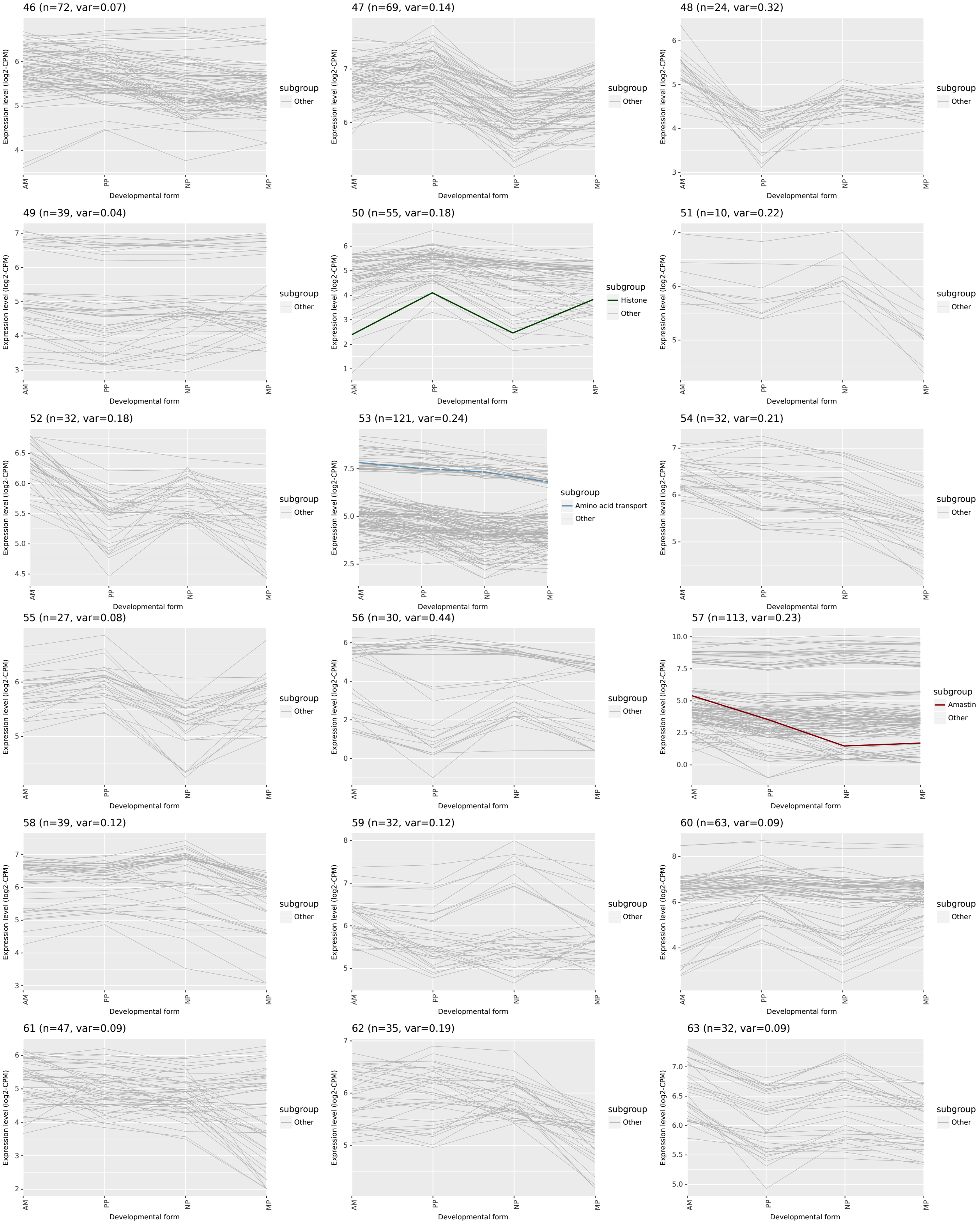
46 (n=72, var=0.07)
47 (n=69, var=0.14)
48 (n=24, var=0.32)
49 (n=39, var=0.04)
50 (n=55, var=0.18)
51 (n=10, var=0.22)
52 (n=32, var=0.18)
53 (n=121, var=0.24)
54 (n=32, var=0.21)
55 (n=27, var=0.08)
56 (n=30, var=0.44)
57 (n=113, var=0.23)
58 (n=39, var=0.12)
59 (n=32, var=0.12)
60 (n=63, var=0.09)
61 (n=47, var=0.09)
62 (n=35, var=0.19)
63 (n=32, var=0.09)
Expression level (log2-CPM)
Developmental form
AM
PP
NP
MP
subgroup
Other
Histone
Amino acid transport
Amastin

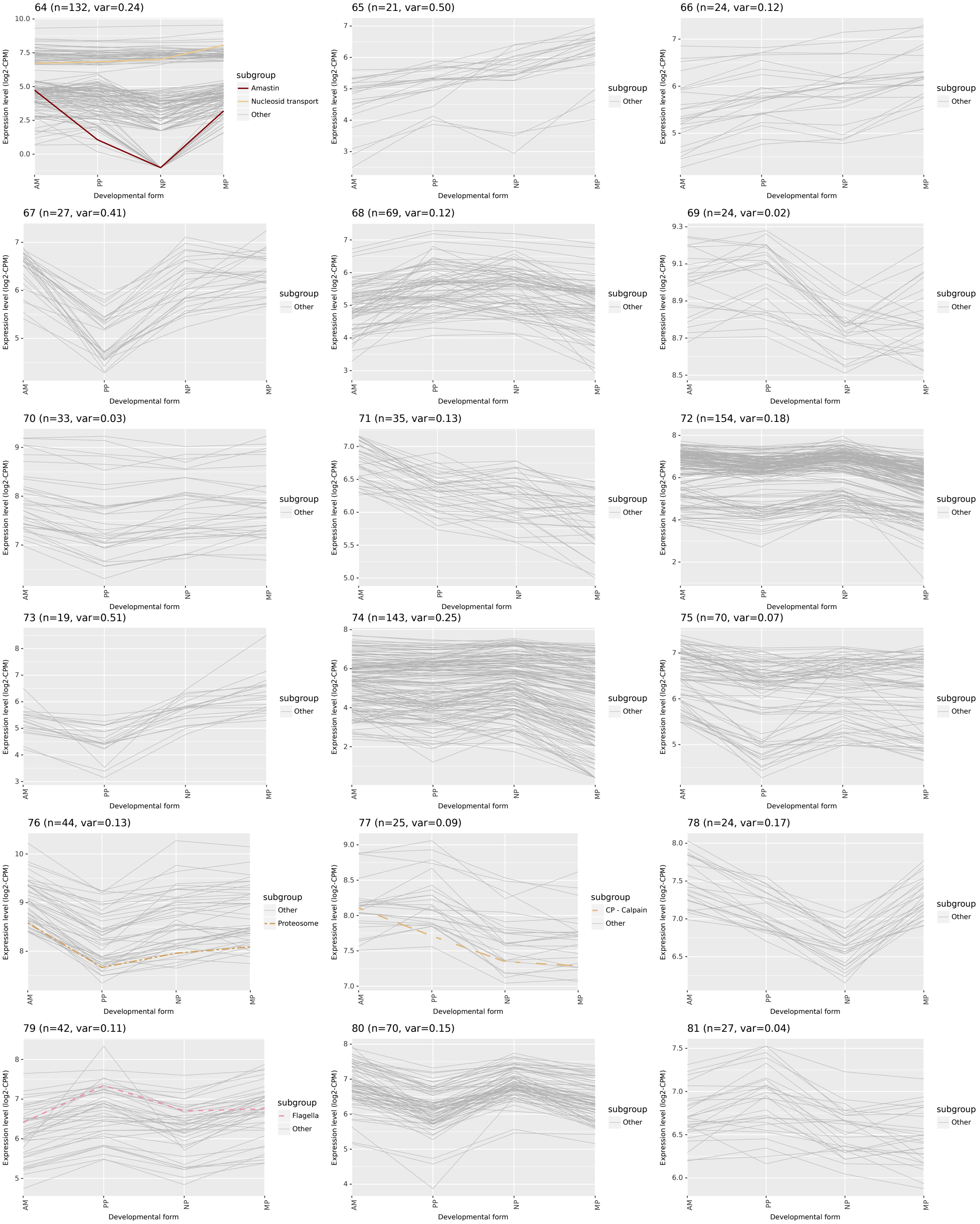
64 (n=132, var=0.24)
subgroup
Amastin
Nucleosid transport
Other
65 (n=21, var=0.50)
subgroup
Other
66 (n=24, var=0.12)
subgroup
Other
67 (n=27, var=0.41)
subgroup
Other
68 (n=69, var=0.12)
subgroup
Other
69 (n=24, var=0.02)
subgroup
Other
70 (n=33, var=0.03)
subgroup
Other
71 (n=35, var=0.13)
subgroup
Other
72 (n=154, var=0.18)
subgroup
Other
73 (n=19, var=0.51)
subgroup
Other
74 (n=143, var=0.25)
subgroup
Other
75 (n=70, var=0.07)
subgroup
Other
76 (n=44, var=0.13)
subgroup
Other
Proteosome
77 (n=25, var=0.09)
subgroup
CP - Calpain
Other
78 (n=24, var=0.17)
subgroup
Other
79 (n=42, var=0.11)
subgroup
Flagella
Other
80 (n=70, var=0.15)
subgroup
Other
81 (n=27, var=0.04)
subgroup
Other
Expression level (log2-CPM)
Developmental form
AM
PP
NP
MP

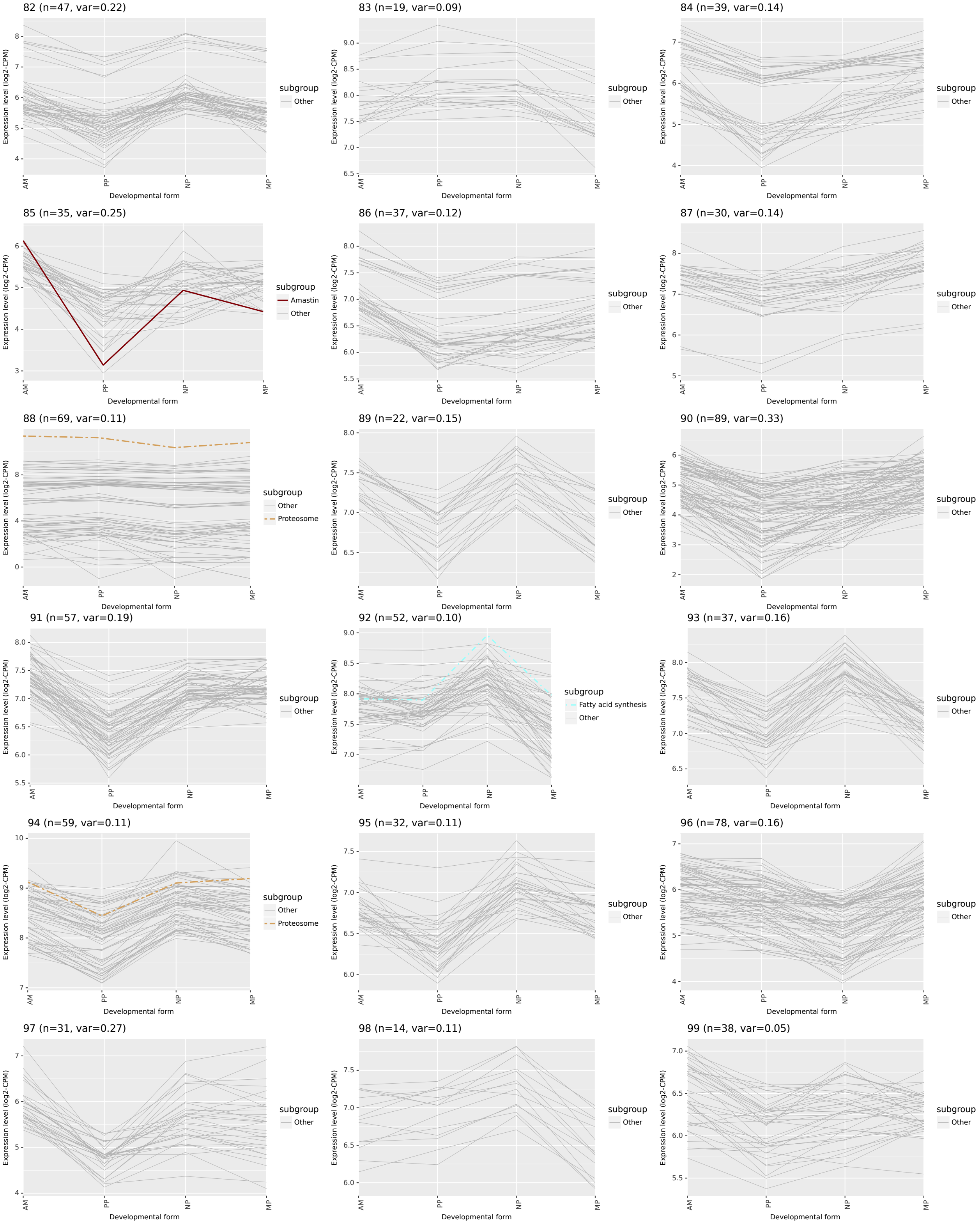
82 (n=47, var=0.22)
83 (n=19, var=0.09)
84 (n=39, var=0.14)
85 (n=35, var=0.25)
86 (n=37, var=0.12)
87 (n=30, var=0.14)
88 (n=69, var=0.11)
89 (n=22, var=0.15)
90 (n=89, var=0.33)
91 (n=57, var=0.19)
92 (n=52, var=0.10)
93 (n=37, var=0.16)
94 (n=59, var=0.11)
95 (n=32, var=0.11)
96 (n=78, var=0.16)
97 (n=31, var=0.27)
98 (n=14, var=0.11)
99 (n=38, var=0.05)
Expression level (log2-CPM)
Developmental form
AM
PP
NP
MP
subgroup
Other
Amastin
Proteosome
Fatty acid synthesis

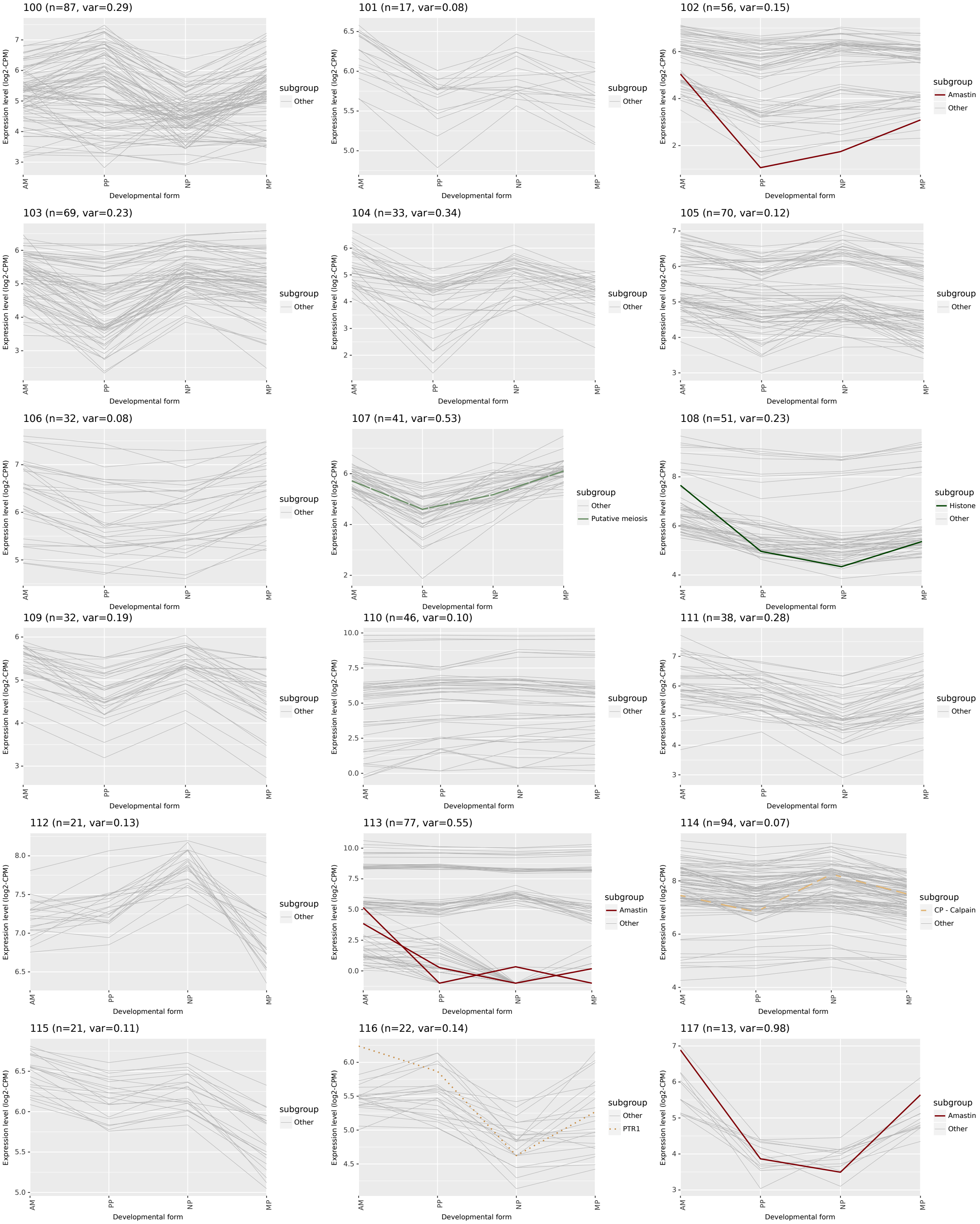
100 (n=87, var=0.29)
101 (n=17, var=0.08)
102 (n=56, var=0.15)
103 (n=69, var=0.23)
104 (n=33, var=0.34)
105 (n=70, var=0.12)
106 (n=32, var=0.08)
107 (n=41, var=0.53)
108 (n=51, var=0.23)
109 (n=32, var=0.19)
110 (n=46, var=0.10)
111 (n=38, var=0.28)
112 (n=21, var=0.13)
113 (n=77, var=0.55)
114 (n=94, var=0.07)
115 (n=21, var=0.11)
116 (n=22, var=0.14)
117 (n=13, var=0.98)
Expression level (log2-CPM)
Developmental form
AM
PP
NP
MP
subgroup
Other
Amastin
Putative meiosis
Histone
CP - Calpain
PTR1

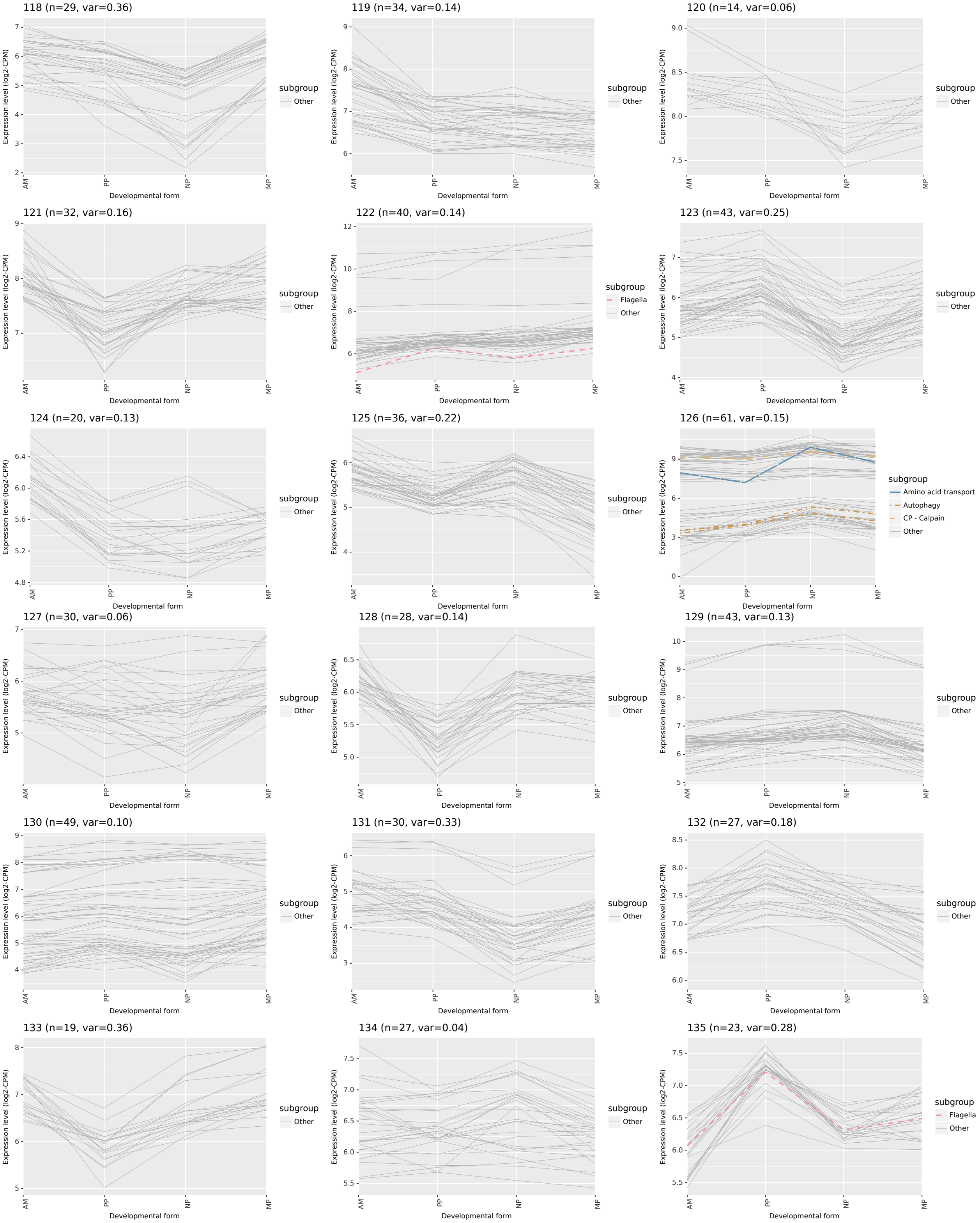

118 (n=29, var=0.36)
119 (n=34, var=0.14)
120 (n=14, var=0.06)
121 (n=32, var=0.16)
122 (n=40, var=0.14)
123 (n=43, var=0.25)
124 (n=20, var=0.13)
125 (n=36, var=0.22)
126 (n=61, var=0.15)
127 (n=30, var=0.06)
128 (n=28, var=0.14)
129 (n=43, var=0.13)
130 (n=49, var=0.10)
131 (n=30, var=0.33)
132 (n=27, var=0.18)
133 (n=19, var=0.36)
134 (n=27, var=0.04)
135 (n=23, var=0.28)
Expression level (log2-CPM)
Developmental form
AM
PP
NP
MP
subgroup
Other
Flagella
Amino acid transport
Autophagy
CP - Calpain

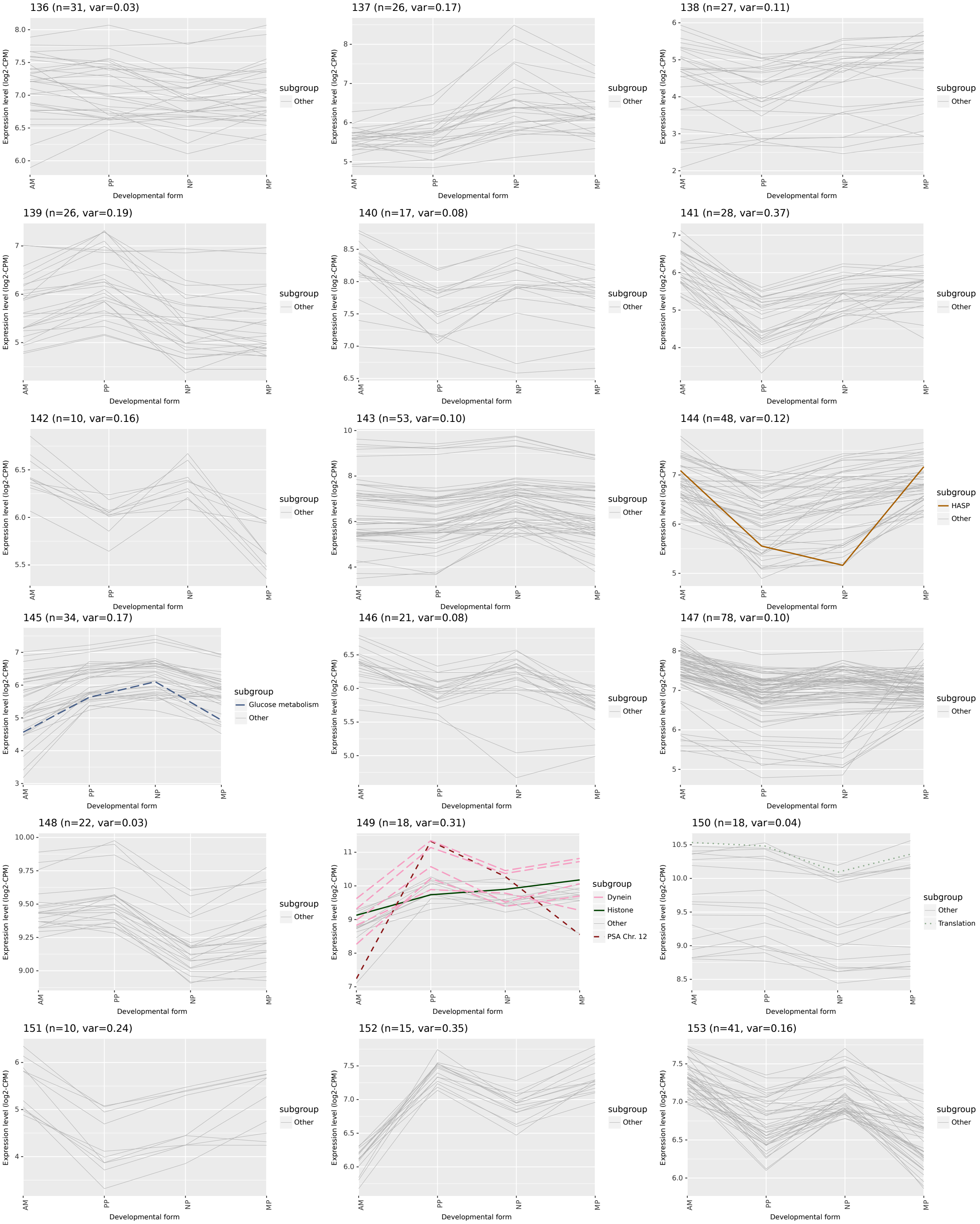
136 (n=31, var=0.03)
137 (n=26, var=0.17)
138 (n=27, var=0.11)
139 (n=26, var=0.19)
140 (n=17, var=0.08)
141 (n=28, var=0.37)
142 (n=10, var=0.16)
143 (n=53, var=0.10)
144 (n=48, var=0.12)
145 (n=34, var=0.17)
146 (n=21, var=0.08)
147 (n=78, var=0.10)
148 (n=22, var=0.03)
149 (n=18, var=0.31)
150 (n=18, var=0.04)
151 (n=10, var=0.24)
152 (n=15, var=0.35)
153 (n=41, var=0.16)
Expression level (log2-CPM)
Developmental form
AM
PP
NP
MP
subgroup
Other
HASP
Glucose metabolism
Dynein
Histone
PSA Chr. 12
Translation

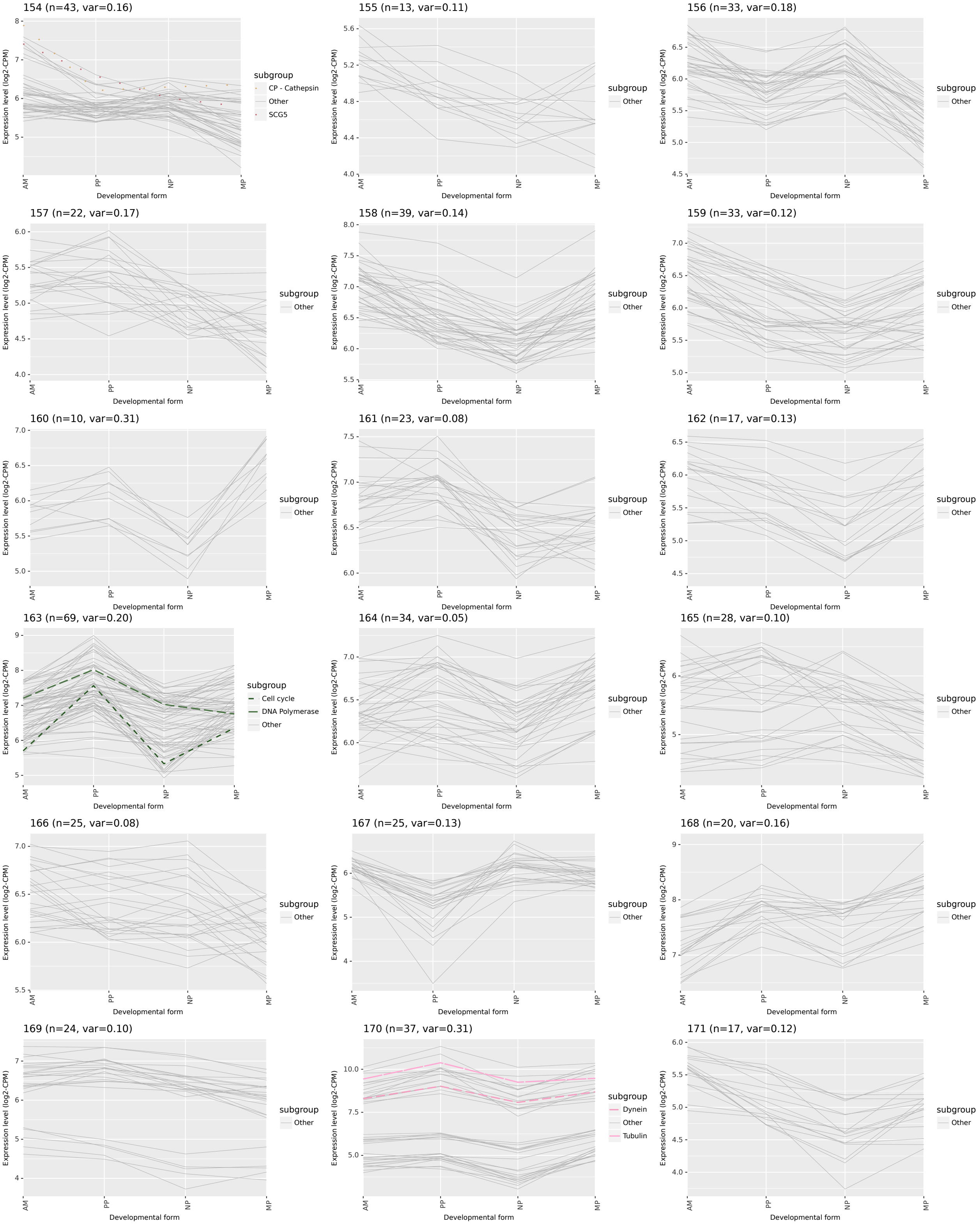
154 (n=43, var=0.16)
subgroup
CP - Cathepsin
Other
SCG5
155 (n=13, var=0.11)
subgroup
Other
156 (n=33, var=0.18)
subgroup
Other
157 (n=22, var=0.17)
subgroup
Other
158 (n=39, var=0.14)
subgroup
Other
159 (n=33, var=0.12)
subgroup
Other
160 (n=10, var=0.31)
subgroup
Other
161 (n=23, var=0.08)
subgroup
Other
162 (n=17, var=0.13)
subgroup
Other
163 (n=69, var=0.20)
subgroup
Cell cycle
DNA Polymerase
Other
164 (n=34, var=0.05)
subgroup
Other
165 (n=28, var=0.10)
subgroup
Other
166 (n=25, var=0.08)
subgroup
Other
167 (n=25, var=0.13)
subgroup
Other
168 (n=20, var=0.16)
subgroup
Other
169 (n=24, var=0.10)
subgroup
Other
170 (n=37, var=0.31)
subgroup
Dynein
Other
Tubulin
171 (n=17, var=0.12)
subgroup
Other
Expression level (log2-CPM)
Developmental form
AM
PP
NP
MP

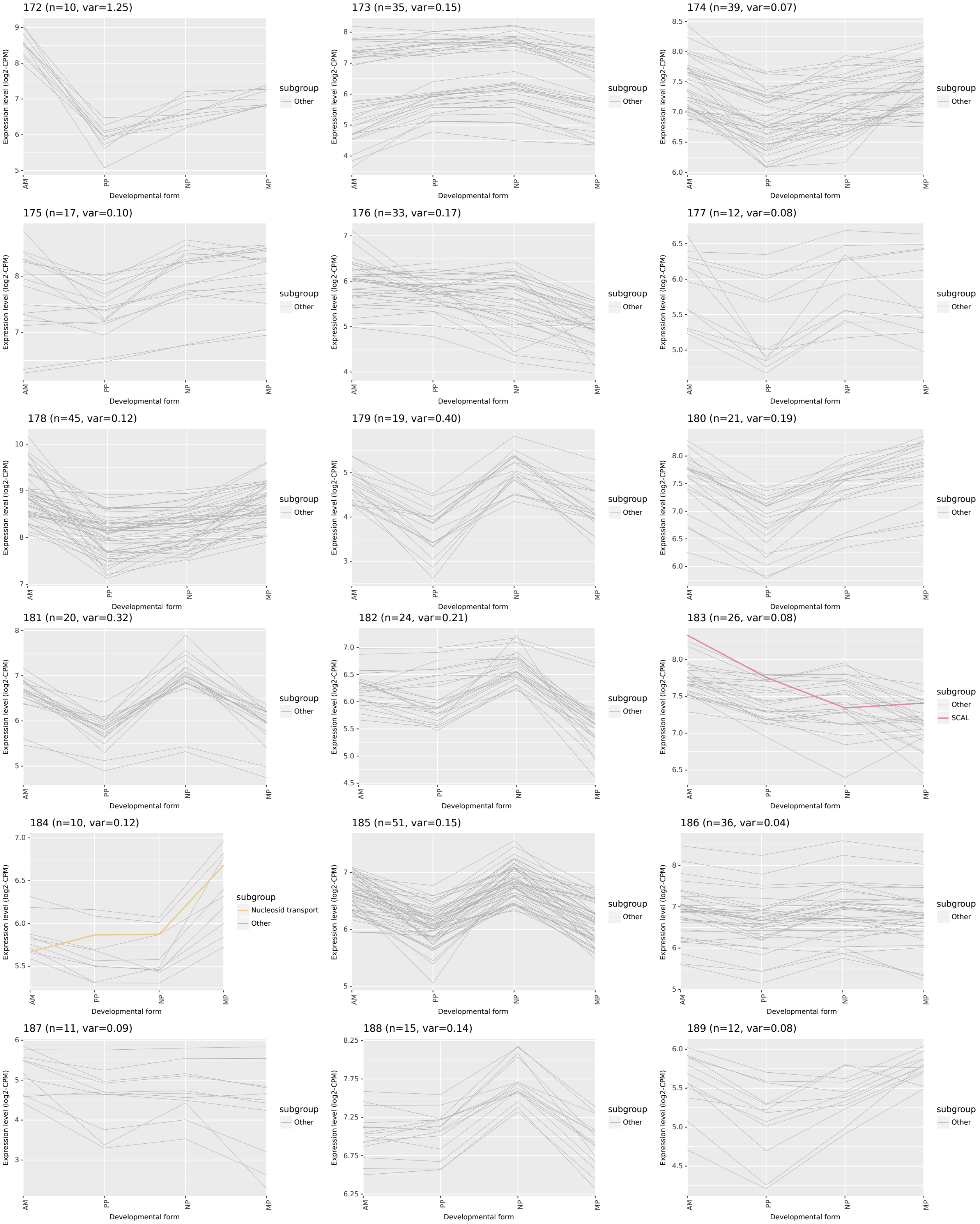
172 (n=10, var=1.25)
173 (n=35, var=0.15)
174 (n=39, var=0.07)
175 (n=17, var=0.10)
176 (n=33, var=0.17)
177 (n=12, var=0.08)
178 (n=45, var=0.12)
179 (n=19, var=0.40)
180 (n=21, var=0.19)
181 (n=20, var=0.32)
182 (n=24, var=0.21)
183 (n=26, var=0.08)
184 (n=10, var=0.12)
185 (n=51, var=0.15)
186 (n=36, var=0.04)
187 (n=11, var=0.09)
188 (n=15, var=0.14)
189 (n=12, var=0.08)
Expression level (log2-CPM)
Developmental form
AM
PP
NP
MP
subgroup
Other
SCAL
Nucleosid transport

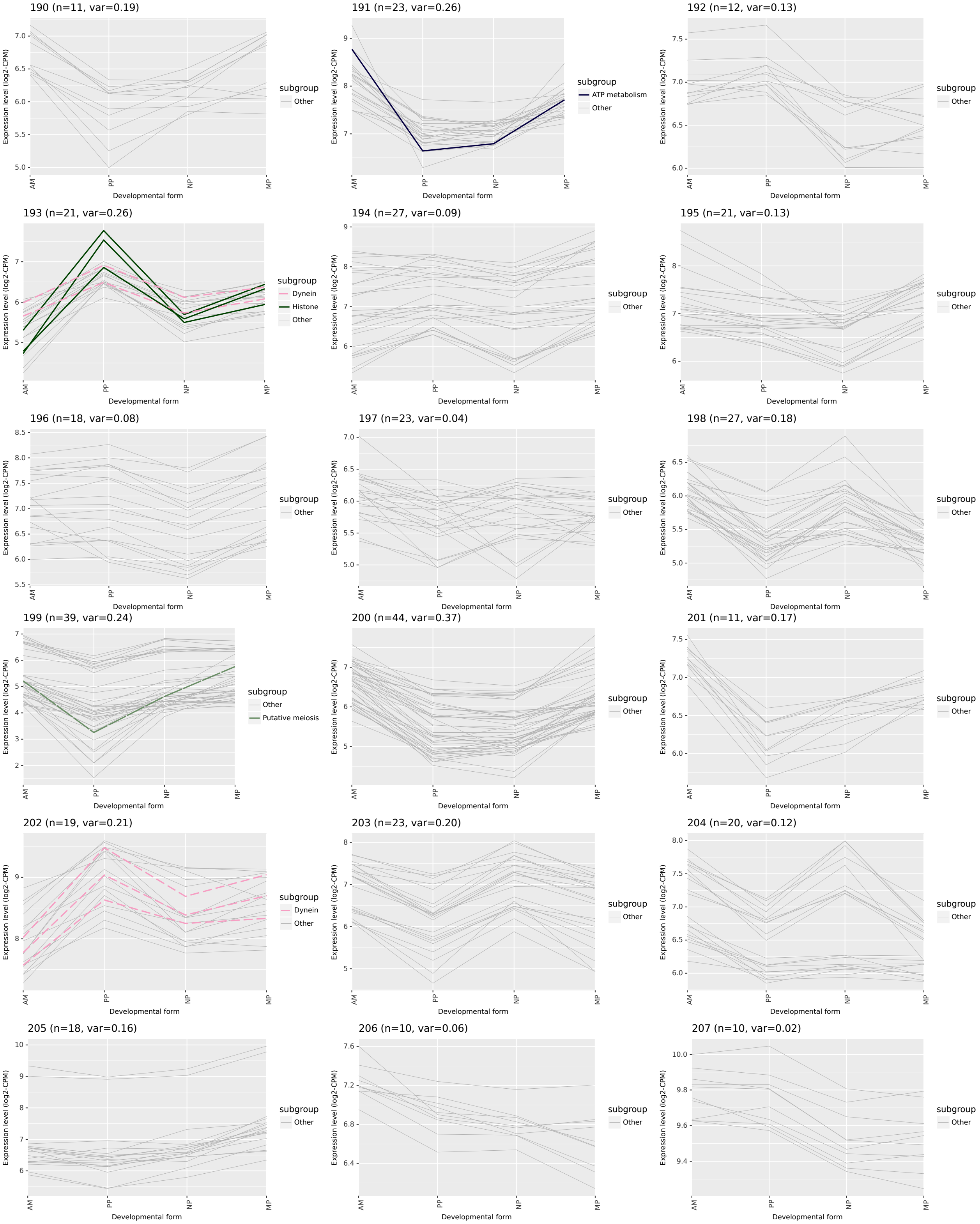
190 (n=11, var=0.19)
191 (n=23, var=0.26)
192 (n=12, var=0.13)
193 (n=21, var=0.26)
194 (n=27, var=0.09)
195 (n=21, var=0.13)
196 (n=18, var=0.08)
197 (n=23, var=0.04)
198 (n=27, var=0.18)
199 (n=39, var=0.24)
200 (n=44, var=0.37)
201 (n=11, var=0.17)
202 (n=19, var=0.21)
203 (n=23, var=0.20)
204 (n=20, var=0.12)
205 (n=18, var=0.16)
206 (n=10, var=0.06)
207 (n=10, var=0.02)
Expression level (log2-CPM)
Developmental form
AM
PP
NP
MP
subgroup
Other
ATP metabolism
Dynein
Histone
Putative meiosis

Average expression profiles for selected co-expression modules

```r
#'
#' Takes a set of subgroup names, finds the dominant co-expression module for
#' each subgroup, and plots the average co-expression profile for each of
#' those modules.
#'
plot_dominant_coex_modules <- function(counts_long, genes_of_interest, subgroups, plot_title='',
                                       additional_modules=NULL) {
    # find dominant module for each subgroup
    dominant_modules <- genes_of_interest %>%
        filter(subgroup %in% subgroups) %>%
        group_by(subgroup) %>%
        summarize(dominant_module=names(which.max(table(module_color))))

    # Include any other additional modules manually requested
    if (!is.null(additional_modules)) {
        dominant_modules <- rbind(dominant_modules, additional_modules)
    }

    # Work-around: manually add alpha- and beta-tubulin modules (14 & 15)
    if (subgroup == 'Surface and structure') {
        dominant_modules <- rbind(dominant_modules,
                                  data.frame(subgroup=c('Tubulin', 'Tubulin'),
                                             dominant_module=c('blueviolet',
                                                               'salmon1')))
    }

    # add module numbers
    dominant_modules <- merge(dominant_modules, module_label_mapping,
                              by.x='dominant_module', by.y='color')

    # compute average expression profiles for each of the dominant modules
    average_profiles <- counts_long %>%
        filter(cluster %in% dominant_modules$number) %>%
        group_by(condition, cluster) %>%
        summarize(average_log_cpm=median(log_cpm))

    # add labels
    x <- dominant_modules[match(average_profiles$cluster, dominant_modules$number),]
    average_profiles$Module <- sprintf("%s\n(module %s)", x$subgroup, x$number)

    # work-around for glucose/AA transport (both associated with the same
    # co-expression module)
    average_profiles$Module[average_profiles$Module == 'Amino acid transport\n(Module 17)'] <- 'Glucose[illegible]

    # work-around for DNA polymerase (similar issue to above)
    average_profiles$Module[average_profiles$Module == 'Cell cycle\n(Module 28)'] <- 'DNA Polymerase\n([illegible]

    # get color palette
    pal <- colorRampPalette(brewer.pal(8, 'Set2'))(nrow(dominant_modules))

    # generate plot
```

```r
    ggplot(average_profiles,
           aes(x=condition, y=average_log_cpm, group=Module, colour=Module)) +
           geom_line(size=1.5) +
           xlab("Developmental stage") +
           ylab("Expression level (log2-CPM)") +
           scale_color_manual(values=pal) +
           scale_x_discrete(expand=c(0,0)) +
           theme(axis.text=element_text(colour="#333333", size=16),
                 axis.text.x=element_text(hjust=1),
                 legend.key.size = unit(2.5, 'lines'))
}

# extend genes of interest list to include module colors/numbers
genes_of_interest <- merge(genes_of_interest,
                           result %>% select(gene_id, module=color))
genes_of_interest <- merge(genes_of_interest,
                           module_label_mapping %>% transmute(module=number, module_color=color))

# specific functions and modules to generate average expression plots for
subgroups_of_interest <- list(
    'Surface and structure'=c(
        'Amastin', 'PSA Chr. 4', 'PSA Chr. 12', 'GP-63', 'LPG3', 'SCG1',
        'Flagella', 'Tubulin'),
    'Metabolism'=c(
        'ATP metabolism', 'Glucose transport', 'Glucose metabolism',
        'Amino acid transport', 'GDH', 'Ketone bodies', 'Fatty acid synthesis'),
    'Cell cycle'=c('Histone', 'Cell cycle', 'Translation', 'Putative meiosis'),
    'Differentiation, stress, and virulence factors'=c(
        'SHERP', 'Meta', 'Autophagy', 'MBAP2', 'FBT', 'ABC transporters',
        'CP - Cathepsin', 'CP - Calpain', 'HASP')
)

# iterate over subgroups and generate expression profile plots
i = 1

for (subgroup in names(subgroups_of_interest)) {
    # For Metabolism plot, also include relevant cluster 20 (pink1)
    if (subgroup == 'Metabolism') {
        additional_modules <- rbind(c('Amino acid transport', 'pink1'),
                                    c('ATP metabolism / P27', 'green3'))
        colnames(additional_modules) <- c('subgroup', 'dominant_module')

        plt <- plot_dominant_coex_modules(counts_long, genes_of_interest,
                                          subgroups_of_interest[[subgroup]],
                                          subgroup, additional_modules)
    } else if (subgroup == 'Cell cycle') {
        # additional modules to include in the plot
        additional_modules <- rbind(c('Putative meiosis', 'palevioletred1'),
                                    c('Cell cycle', 'honeydew1'))
        colnames(additional_modules) <- c('subgroup', 'dominant_module')

        plt <- plot_dominant_coex_modules(counts_long, genes_of_interest,
                                          subgroups_of_interest[[subgroup]],
```

```
                                          subgroup,
                                          additional_modules)

    } else {
        plt <- plot_dominant_coex_modules(counts_long, genes_of_interest,
                                          subgroups_of_interest[[subgroup]],
                                          subgroup)
        # convert to gtable to allow for normalizing dimensions in
        # the plot grid
        #plt <- ggplot_gtable(ggplot_build(plt))
    }
    assign(sprintf('plt%d', i), plt)
    i = i + 1
}

# normalize widths of plots
# http://stackoverflow.com/a/19321139/554531
plots <- list(plt1, plt2, plt3, plt4)
grobs <- list()
widths <- list()

for (i in 1:length(plots)){
    grobs[[i]] <- ggplotGrob(plots[[i]])
    widths[[i]] <- grobs[[i]]$widths[2:5]
}

maxwidth <- do.call(grid::unit.pmax, widths)
for (i in 1:length(grobs)){
     grobs[[i]]$widths[2:5] <- as.list(maxwidth)
}

# plot as a 2x2 grid
do.call("grid.arrange", c(grobs, ncol=2))
```

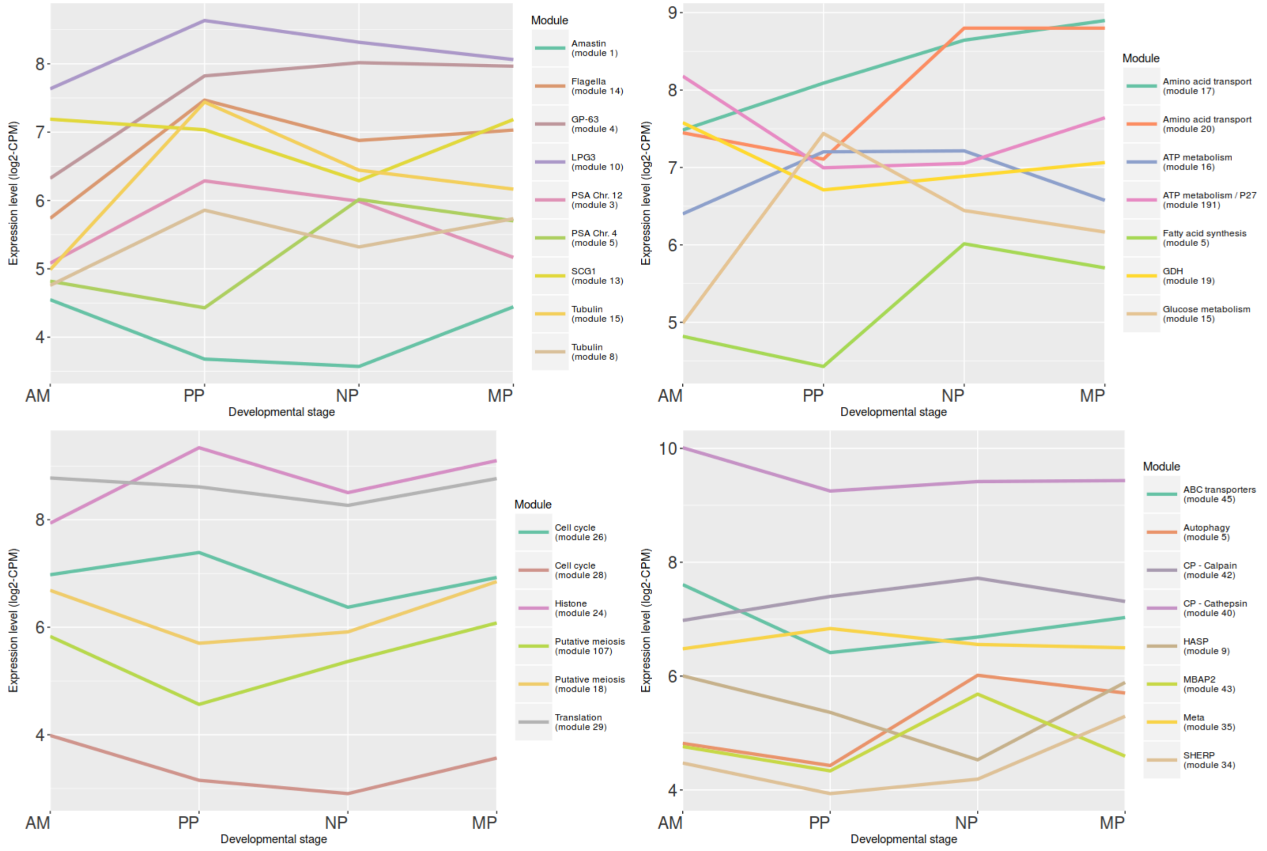

Enrichment

GO Enrichment

```r
# Create gene lengths vector
gene_ids <- rownames(network_counts$final)

gene_lengths <- gene_info$transcript_length
names(gene_lengths) <- gene_info$gene_id
```

```r
# Initiallize parallelization
cl <- makeCluster(min(12, detectCores() - 1))
registerDoParallel(cl)

# Check each module for enrichment in GO terms and save result in a list
module_go_enrichment <- foreach(color=unique(module_colors), .packages=c('goseq')) %dopar% {
    # Measure GO enrichment for module
    enriched <- tryCatch({
        # module gene ids
        in_module_geneids <- gene_ids[module_colors == color]

        enriched <- test_gene_enrichment(in_module_geneids, gene_ids,
                                         gene_go_mapping, gene_lengths)

        # Add descriptions
        #enriched <- merge(enriched, go_term_id_mapping, by='category')
```

```
    }, error=function(e) {
        # goseq fails in some cases; have not been able to track down cause yet
        # to avoid errors we will just return an empty result set
        warning(sprintf("GO enrichment failed for module %s", color))
        cbind(
            get_enrichment_placeholder(),
            term=numeric(0),
            ontology=numeric(0)
        )
    })
    enriched
}
names(module_go_enrichment) <- unique(module_colors)

# remove any null entries from the results
module_go_enrichment <- module_go_enrichment[!sapply(module_go_enrichment, is.null)]

# unregister cpus
stopCluster(cl)
```

```
# Print enrichment results
# TODO: Refactor enrichment results printing logic
tmp <- cbind(gene=gene_ids, color=module_colors)
gene_mapping <- merge(gene_go_mapping, tmp, by='gene')

if (CONFIG$include_tables) {
    print_enrichment_results(module_go_enrichment, module_sizes, 'GO terms',
                             go_term_id_mapping, gene_mapping, 'output/modules',
                             'go', include_gene_lists=FALSE)
}
```

8 enrichment (106 genes)

Over-represented GO terms:

category	adj_pval	num_in_subset	num_total	term	ontology
GO:0000786	0.0282	5	29	nucleosome	CC
GO:0003924	0.0018	9	86	GTPase activity	MF
GO:0005525	0.0282	9	128	GTP binding	MF
GO:0006334	0.0338	5	33	nucleosome assembly	BP
GO:0043234	0.0000	8	36	protein complex	CC
GO:0046982	0.0293	5	31	protein heterodimerization activity	MF
GO:0051258	0.0000	8	35	protein polymerization	BP

65 enrichment (21 genes)

Over-represented GO terms:

category	adj_pval	num_in_subset	num_total	term	ontology
GO:0022891	0.0088	2	2	substrate-specific transmembrane transporter activity	MF

69 enrichment (24 genes)

Over-represented GO terms:

category	adj_pval	num_in_subset	num_total	term	ontology
GO:0003735	0e+00	19	166	structural constituent of ribosome	MF
GO:0005622	0e+00	15	299	intracellular	CC
GO:0005840	0e+00	19	157	ribosome	CC
GO:0006412	0e+00	19	171	translation	BP
GO:0015935	2e-04	5	16	small ribosomal subunit	CC

24 enrichment (46 genes)

Over-represented GO terms:

category	adj_pval	num_in_subset	num_total	term	ontology
GO:0000786	0.0000	7	29	nucleosome	CC
GO:0003677	0.0236	7	161	DNA binding	MF
GO:0005634	0.0057	7	149	nucleus	CC
GO:0006334	0.0000	7	33	nucleosome assembly	BP
GO:0043234	0.0030	4	36	protein complex	CC
GO:0046982	0.0000	7	31	protein heterodimerization activity	MF
GO:0051258	0.0030	4	35	protein polymerization	BP

98 enrichment (14 genes)

Over-represented GO terms:

category	adj_pval	num_in_subset	num_total	term	ontology
GO:0004812	0.0125	3	24	aminoacyl-tRNA ligase activity	MF
GO:0006418	0.0125	3	24	tRNA aminoacylation for protein translation	BP

14 enrichment (30 genes)

Over-represented GO terms:

category	adj_pval	num_in_subset	num_total	term	ontology
GO:0003924	0.0017	6	86	GTPase activity	MF
GO:0005516	0.0000	6	12	calmodulin binding	MF
GO:0005525	0.0130	6	128	GTP binding	MF
GO:0031514	0.0000	6	28	motile cilium	CC
GO:0043234	0.0000	6	36	protein complex	CC
GO:0051258	0.0000	6	35	protein polymerization	BP

110 enrichment (46 genes)

Over-represented GO terms:

category	adj_pval	num_in_subset	num_total	term	ontology

category	adj_pval	num_in_subset	num_total	term	ontology
GO:0004478	0.0097	2	2	methionine adenosyltransferase activity	MF
GO:0006556	0.0097	2	2	S-adenosylmethionine biosynthetic process	BP
GO:0015948	0.0097	2	2	methanogenesis	BP

113 enrichment (77 genes)

Over-represented GO terms:

category	adj_pval	num_in_subset	num_total	term	ontology
GO:0006412	0.0327	13	171	translation	BP

120 enrichment (14 genes)

Over-represented GO terms:

category	adj_pval	num_in_subset	num_total	term	ontology
GO:0003735	0.0027	7	166	structural constituent of ribosome	MF
GO:0005840	0.0027	7	157	ribosome	CC

132 enrichment (27 genes)

Over-represented GO terms:

category	adj_pval	num_in_subset	num_total	term	ontology
GO:0044267	0.0309	3	12	cellular protein metabolic process	BP

30 enrichment (28 genes)

Over-represented GO terms:

category	adj_pval	num_in_subset	num_total	term	ontology
GO:0003746	0.037	3	15	translation elongation factor activity	MF
GO:0003924	0.037	5	86	GTPase activity	MF
GO:0005525	0.037	6	128	GTP binding	MF

143 enrichment (53 genes)

Over-represented GO terms:

category	adj_pval	num_in_subset	num_total	term
GO:0016593	0.0495	2	2	Cdc73/Paf1 complex
GO:0032968	0.0495	2	2	positive regulation of transcription elongation from RNA polyme

148 enrichment (22 genes)

Over-represented GO terms:

category	adj_pval	num_in_subset	num_total	term	ontology
GO:0003735	0	18	166	structural constituent of ribosome	MF
GO:0005622	0	15	299	intracellular	CC
GO:0005840	0	17	157	ribosome	CC
GO:0006412	0	18	171	translation	BP

149 enrichment (18 genes)

Over-represented GO terms:

category	adj_pval	num_in_subset	num_total	term	ontology
GO:0030286	0.0054	7	15	dynein complex	CC

4 enrichment (21 genes)

Over-represented GO terms:

category	adj_pval	num_in_subset	num_total	term
GO:0004198	0.0328	3	23	calcium-dependent cysteine-type endopeptidase activity
GO:0006508	0.0132	5	91	proteolysis
GO:0007155	0.0328	2	6	cell adhesion
GO:0008160	0.0164	2	4	protein tyrosine phosphatase activator activity
GO:0042025	0.0164	2	4	host cell nucleus
GO:0044081	0.0164	2	4	modulation by symbiont of host nitric oxide-mediated signal tran
GO:0075130	0.0164	2	4	modulation by symbiont of host protein kinase-mediated signal t

150 enrichment (18 genes)

Over-represented GO terms:

category	adj_pval	num_in_subset	num_total	term	ontology
GO:0003735	0e+00	15	166	structural constituent of ribosome	MF
GO:0005622	1e-04	11	299	intracellular	CC
GO:0005840	0e+00	15	157	ribosome	CC
GO:0006412	0e+00	14	171	translation	BP

29 enrichment (17 genes)

Over-represented GO terms:

category	adj_pval	num_in_subset	num_total	term	ontology
GO:0003735	0	13	166	structural constituent of ribosome	MF
GO:0005622	0	12	299	intracellular	CC
GO:0005840	0	13	157	ribosome	CC
GO:0006412	0	11	171	translation	BP

32 enrichment (28 genes)

Over-represented GO terms:

category	adj_pval	num_in_subset	num_total	term	ontology
GO:0003735	0.0000	19	166	structural constituent of ribosome	MF
GO:0005622	0.0000	17	299	intracellular	CC
GO:0005840	0.0000	17	157	ribosome	CC
GO:0006412	0.0000	18	171	translation	BP
GO:0015934	0.0051	3	10	large ribosomal subunit	CC

15 enrichment (29 genes)

Over-represented GO terms:

category	adj_pval	num_in_subset	num_total	term	ontology
GO:0003924	0.0005	6	86	GTPase activity	MF
GO:0005525	0.0038	6	128	GTP binding	MF
GO:0043234	0.0000	6	36	protein complex	CC
GO:0051258	0.0000	6	35	protein polymerization	BP

172 enrichment (10 genes)

Over-represented GO terms:

category	adj_pval	num_in_subset	num_total	term	ontology
GO:0004197	0.0250	2	7	cysteine-type endopeptidase activity	MF
GO:0004519	0.0009	3	15	endonuclease activity	MF
GO:0006308	0.0004	3	9	DNA catabolic process	BP

16 enrichment (25 genes)

Over-represented GO terms:

category	adj_pval	num_in_subset	num_total	term
GO:0000275	0.0468	2	4	mitochondrial proton-transporting ATP synthase complex, catal
GO:0004252	0.0468	2	5	serine-type endopeptidase activity
GO:0015991	0.0468	3	23	ATP hydrolysis coupled proton transport
GO:0046034	0.0468	2	4	ATP metabolic process
GO:0070012	0.0298	2	2	oligopeptidase activity

17 enrichment (53 genes)

Over-represented GO terms:

category	adj_pval	num_in_subset	num_total	term	ontology
GO:0002036	0.0001	3	3	regulation of L-glutamate transport	BP
GO:0005353	0.0001	3	3	fructose transmembrane transporter activity	MF
GO:0005354	0.0001	3	3	galactose transmembrane transporter activity	MF

category	adj_pval	num_in_subset	num_total	term	ontology
GO:0005886	0.0007	4	15	plasma membrane	CC
GO:0006971	0.0001	3	4	hypotonic response	BP
GO:0009190	0.0015	4	12	cyclic nucleotide biosynthetic process	BP
GO:0015193	0.0001	3	3	L-proline transmembrane transporter activity	MF
GO:0015238	0.0015	3	7	drug transmembrane transporter activity	MF
GO:0015578	0.0001	3	3	mannose transmembrane transporter activity	MF
GO:0015591	0.0046	2	2	D-ribose transmembrane transporter activity	MF
GO:0015752	0.0046	2	2	D-ribose transport	BP
GO:0015758	0.0001	3	3	glucose transport	BP
GO:0015893	0.0001	3	3	drug transport	BP
GO:0016849	0.0015	4	12	phosphorus-oxygen lyase activity	MF
GO:0022857	0.0012	3	6	transmembrane transporter activity	MF
GO:0022858	0.0001	3	3	alanine transmembrane transporter activity	MF
GO:0035524	0.0001	3	3	proline transmembrane transport	BP
GO:0035556	0.0046	4	19	intracellular signal transduction	BP
GO:0051955	0.0001	3	3	regulation of amino acid transport	BP
GO:0055056	0.0046	2	2	D-glucose transmembrane transporter activity	MF
GO:0070258	0.0046	2	2	inner membrane complex	CC
GO:0070881	0.0001	3	3	regulation of proline transport	BP
GO:0080144	0.0001	3	3	amino acid homeostasis	BP

40 enrichment (23 genes)

Over-represented GO terms:

category	adj_pval	num_in_subset	num_total	term	ontology
GO:0006950	0.0030	3	21	response to stress	BP
GO:0008234	0.0014	3	13	cysteine-type peptidase activity	MF
GO:0016209	0.0002	3	8	antioxidant activity	MF
GO:0051082	0.0388	3	53	unfolded protein binding	MF
GO:0051920	0.0002	3	8	peroxiredoxin activity	MF

207 enrichment (10 genes)

Over-represented GO terms:

category	adj_pval	num_in_subset	num_total	term	ontology
GO:0003735	0.0000	8	166	structural constituent of ribosome	MF
GO:0005622	0.0299	6	299	intracellular	CC
GO:0005840	0.0000	9	157	ribosome	CC
GO:0006412	0.0000	8	171	translation	BP
GO:0032465	0.0073	2	3	regulation of cytokinesis	BP

Total enriched GO terms

Total: 110 (Mean adjusted pval=0.009336)

```
enriched_colors_go <- get_enriched_modules(module_go_enrichment)

# save results
```

```
COEXPRESSION_NETWORK_RESULT$enriched_colors_go <- enriched_colors_go
COEXPRESSION_NETWORK_RESULT$module_go_enrichment <- module_go_enrichment
COEXPRESSION_NETWORK_RESULT$go_term_id_mapping <- go_term_id_mapping
```

KEGG Enrichment

```
# Check each module for enrichment in KEGG terms and save result in a list
cl <- makeCluster(min(12, detectCores() - 1))
registerDoParallel(cl)

# Check each module for enrichment in GO terms and save result in a list
module_kegg_enrichment <- foreach(color=unique(module_colors), .packages=c('goseq')) %dopar% {
    # Measure KEGG enrichment for module
    enriched <- tryCatch({
        in_module_geneids <- gene_ids[module_colors == color]
        enriched <- test_gene_enrichment(in_module_geneids, gene_ids,
                                        gene_kegg_mapping, gene_lengths)
        enriched <- unique(merge(enriched, kegg_pathways[,c('category','name')],
                                by='category'))
    }, error=function(e) {
        # goseq fails in some cases; have not been able to track down cause yet
        warning(sprintf("KEGG enrichment failed for module %s", color))
        return(get_enrichment_placeholder())
    })
    enriched
}

names(module_kegg_enrichment) <- unique(module_colors)

# remove any null entries from the results
module_kegg_enrichment <- module_kegg_enrichment[!sapply(module_kegg_enrichment, is.null)]

# unregister cpus
stopCluster(cl)
```

```
if (CONFIG$include_tables) {
    print_enrichment_results(module_kegg_enrichment, module_sizes, 'KEGG pathway',
                                kegg_pathways %>% select(-description),
                                'output/modules', 'kegg')
}
```

8 enrichment (106 genes)

Over-represented KEGG pathway:

category	adj_pval	num_in_subset	num_total	name	class
path:lma04145	5e-04	8	61	Phagosome	Cellular Processes; Transport and catabolism

69 enrichment (24 genes)

Over-represented KEGG pathway:

category	adj_pval	num_in_subset	num_total	name	class
path:lma03010	0	24	169	Ribosome	Genetic Information Processing; Translation

Under-represented KEGG pathway:

category	adj_pval	num_in_subset	num_total	name	class
path:lma01100	0.0139	0	426	Metabolic pathways	

70 enrichment (33 genes)

Over-represented KEGG pathway:

category	adj_pval	num_in_subset	num_total	name	class
path:lma03010	0.0446	4	169	Ribosome	Genetic Information Processing; Translation

72 enrichment (154 genes)

Over-represented KEGG pathway:

category	adj_pval	num_in_subset	num_total	name	class
path:lma00563	0.0092	3	8	Glycosylphosphatidylinositol(GPI)-anchor biosynthesis	Meta

24 enrichment (46 genes)

Over-represented KEGG pathway:

category	adj_pval	num_in_subset	num_total	name	class
path:lma04145	0.035	4	61	Phagosome	Cellular Processes; Transport and catabolism

98 enrichment (14 genes)

Over-represented KEGG pathway:

category	adj_pval	num_in_subset	num_total	name	class
path:lma00970	0.0113	3	28	Aminoacyl-tRNA biosynthesis	Genetic Information Processin

14 enrichment (30 genes)

Over-represented KEGG pathway:

category	adj_pval	num_in_subset	num_total	name	class
path:lma04145	0	6	61	Phagosome	Cellular Processes; Transport and catabolism

39 enrichment (103 genes)

Over-represented KEGG pathway:

category	adj_pval	num_in_subset	num_total	name	class
path:lma03013	0.0335	5	49	RNA transport	Genetic Information Processing; Translation

113 enrichment (77 genes)

Over-represented KEGG pathway:

category	adj_pval	num_in_subset	num_total	name	class
path:lma03010	1e-04	17	169	Ribosome	Genetic Information Processing; Translation

120 enrichment (14 genes)

Over-represented KEGG pathway:

category	adj_pval	num_in_subset	num_total	name	class
path:lma03010	0.0073	7	169	Ribosome	Genetic Information Processing; Translation

145 enrichment (34 genes)

Over-represented KEGG pathway:

category	adj_pval	num_in_subset	num_total	name	class
path:lma00051	0.0124	3	13	Fructose and mannose metabolism	Metabolism; Carbohydr
path:lma00524	0.0124	2	3	Butirosin and neomycin biosynthesis	Metabolism; Biosynthes

148 enrichment (22 genes)

Over-represented KEGG pathway:

category	adj_pval	num_in_subset	num_total	name	class
path:lma03010	0	21	169	Ribosome	Genetic Information Processing; Translation

Under-represented KEGG pathway:

category	adj_pval	num_in_subset	num_total	name	class
path:lma01100	0.0205	0	426	Metabolic pathways	

150 enrichment (18 genes)

Over-represented KEGG pathway:

category	adj_pval	num_in_subset	num_total	name	class
path:lma03010	0	16	169	Ribosome	Genetic Information Processing; Translation

29 enrichment (17 genes)

Over-represented KEGG pathway:

category	adj_pval	num_in_subset	num_total	name	class
path:lma03010	0	13	169	Ribosome	Genetic Information Processing; Translation

32 enrichment (28 genes)

Over-represented KEGG pathway:

category	adj_pval	num_in_subset	num_total	name	class
path:lma03010	0	22	169	Ribosome	Genetic Information Processing; Translation

Under-represented KEGG pathway:

category	adj_pval	num_in_subset	num_total	name	class
path:lma01100	0.0026	0	426	Metabolic pathways	

15 enrichment (29 genes)

Over-represented KEGG pathway:

category	adj_pval	num_in_subset	num_total	name	class
path:lma00010	0.0384	4	39	Glycolysis / Gluconeogenesis	Metabolism; Carbohydrate met
path:lma04145	0.0019	6	61	Phagosome	Cellular Processes; Transport a

23 enrichment (79 genes)

Over-represented KEGG pathway:

category	adj_pval	num_in_subset	num_total	name	class
path:lma04145	0.0083	4	61	Phagosome	Cellular Processes; Transport and catabolism

172 enrichment (10 genes)

Over-represented KEGG pathway:

category	adj_pval	num_in_subset	num_total	name	class
path:lma05140	0.0268	2	19	Leishmaniasis	Human Diseases; Infectious diseases

40 enrichment (23 genes)

Over-represented KEGG pathway:

category	adj_pval	num_in_subset	num_total	name	class
path:lma00480	0.0222	3	28	Glutathione metabolism	Metabolism; Metabolism of other ar

207 enrichment (10 genes)

Over-represented KEGG pathway:

category	adj_pval	num_in_subset	num_total	name	class
path:lma03010	3e-04	8	169	Ribosome	Genetic Information Processing; Translation

Total enriched KEGG pathway

Total: 25 (Mean adjusted pval=0.012046)

```
enriched_colors_kegg <- get_enriched_modules(module_kegg_enrichment)

# save results
COEXPRESSION_NETWORK_RESULT$enriched_colors_kegg <- enriched_colors_kegg
COEXPRESSION_NETWORK_RESULT$module_kegg_enrichment <- module_kegg_enrichment
COEXPRESSION_NETWORK_RESULT$kegg_pathways <- kegg_pathways
```

```
go_enrichment_status   <- as.numeric(module_colors %in% enriched_colors_go)
kegg_enrichment_status <- as.numeric(module_colors %in% enriched_colors_kegg)
```

LeishCyc enrichment

```
# load data (source: http://www.biocyc.org/LEISH/organism-summary?object=LEISH)
leishcyc_infile <- file.path(Sys.getenv('REF'), 'lmajor_friedlin', 'annotation',
                            'LeishCyc_19.5_mapping.csv')

# load full LeishCyc mapping
leishcyc_mapping <- read.csv(leishcyc_infile)
colnames(leishcyc_mapping) <- c('category', 'gene_id')
```

```
# Initialize CPUs
cl <- makeCluster(min(12, detectCores() - 1))
registerDoParallel(cl)

# Check each module for pathway enrichment and save result in a list
leishcyc_pathway_enrichment <- foreach(color=unique(module_colors), .packages=c('goseq')) %dopar% {
    # Measure enrichment for module
    enriched <- tryCatch({
        in_module_geneids <- gene_ids[module_colors == color]
        enriched <- test_gene_enrichment(in_module_geneids, gene_ids,
                                        leishcyc_mapping, gene_lengths)
    }, error=function(e) {
        # goseq fails in some cases; have not been able to track down cause yet
        # Note: goseq will also fail if supplied with a dplyr tbl_df so first
        # make sure that is not the case
        warning(sprintf("LeishCyc enrichment failed for module %s", color))
        return(get_enrichment_placeholder())
    })
```

```
    enriched
}
names(leishcyc_pathway_enrichment) <- unique(module_colors)

# remove any null entries from the results
leishcyc_pathway_enrichment <- leishcyc_pathway_enrichment[!sapply(leishcyc_pathway_enrichment, is.null

# unregister cpus
stopCluster(cl)
```

```
if (CONFIG$include_tables) {
    print_enrichment_results(leishcyc_pathway_enrichment, module_sizes, 'LeishCyc
                             Pathway', output_dir='output/modules',
                             enrichment_type='leishcyc')
}
```

110 enrichment (46 genes)

Over-represented LeishCyc Pathway:

	category	adj_pval	num_in_subset	num_total
106	S-adenosyl-L-methionine biosynthesis	0.0043	2	2
73	methionine degradation I (to homocysteine)	0.0063	2	3
65	S-adenosyl-L-methionine cycle	0.0174	2	6
74	methionine salvage pathway	0.0304	2	8

15 enrichment (29 genes)

Over-represented LeishCyc Pathway:

	category	adj_pval	num_in_subset	num_total
119	superpathway of central carbon metabolism	0.0056	5	62

40 enrichment (23 genes)

Over-represented LeishCyc Pathway:

	category	adj_pval	num_in_subset	num_total
138	trypanothione redox reactions	7e-04	3	11

194 enrichment (27 genes)

Over-represented LeishCyc Pathway:

	category	adj_pval	num_in_subset	num_total
40	GDP-arabinose biosynthesis	0.0071	2	2
41	GDP-L-fucose biosynthesis II (from L-fucose)	0.0071	2	2

Total enriched LeishCyc

```
Pathway
```

Total: 8 (Mean adjusted pval=0.009882)

```r
# temporarily recreate correlation matrix to compute module stats
correlation_matrix <- cor(t(wgcna_input))
module_stats <- create_module_stats_df(result, correlation_matrix, wgcna_input,
                                       main_contrast)
# free up memory
rm(correlation_matrix)
gc()
```

```r
if ('descriptiion' %in% colnames(result)) {
    sorted_result <- tbl_df(result[with(result, order(color, description)),])
} else {
    sorted_result <- tbl_df(result[with(result, order(color)),])
}

# Also save a simplified version of the table for display in the HTML output
simple_result <- sorted_result
```

```r
# Write out a csv file to be used as a node table input in cytoscape.
write.table(sorted_result,
            file.path(output_dir, "output", "network-annotation-full.tab"),
            row.names=FALSE, quote=FALSE, sep="\t")

# Also save a simplified version of the table for display in the HTML output
write.table(simple_result,
            file.path(output_dir, "output", "network-annotation.tab"),
            row.names=FALSE, quote=FALSE, sep="\t")

# Save dendrogram and module information
save(gene_tree, file=file.path(outdir, 'gene_tree.RData'))
save(module_colors, file=file.path(outdir, 'module_colors.RData'))

# Save GO/KEGG network module enrichment results
save(module_go_enrichment,
     file=file.path(outdir, 'module_go_enrichment.RData'))

save(module_kegg_enrichment,
     file=file.path(outdir, 'module_kegg_enrichment.RData'))

write.csv(combined_counts_table,
          file.path(output_dir, "output", "combined_counts.csv"),
          row.names=FALSE, quote=FALSE)

# Write out a csv file to be used as a node table input in cytoscape.
write.table(sorted_result,
            file.path(output_dir, "output", "network-annotation-full.tab"),
            row.names=FALSE, quote=FALSE, sep="\t")

write.table(simple_result,
            file.path(output_dir, "output", "network-annotation.tab"),
```

```
            row.names=FALSE, quote=FALSE, sep="\t")

# save inidividual module information
for (mod_color in module_colors) {
    # module genes
    module_genes <- result %>% filter(color==mod_color)
    write.table(module_genes,
                file.path(module_output_dir, sprintf("%s_genes.txt", mod_color)),
                row.names=FALSE, quote=FALSE, sep="\t")
}

# Save enrichment results as text files
output_module_enrichment_results(module_go_enrichment, module_output_dir,
                                 'go', go_term_id_mapping)
output_module_enrichment_results(module_kegg_enrichment, module_output_dir,
                                 'kegg', kegg_pathways %>% select(-description))

if (CONFIG$target == "pathogen" && CONFIG$pathogen == "L. major") {
    output_module_enrichment_results(leishcyc_pathway_enrichment,
                                     module_output_dir, 'leishcyc')
}

# Write module stats to a file
write.csv(module_stats, file.path(output_dir, "output", "module_stats.csv"),
          row.names=FALSE, quote=FALSE)
```

Data Preparation

This document describes the steps used for co-expression cluster analysis. It begins with a raw count-table generated using HTSeq. The commands used to go from the raw RNA-Seq reads to a count table are described below. Filepaths containing `69XX` are used representative sample names. In each such case, the commands should be run separately for each sample (e.g. 6961).

Read trimming

Trimmomatic v0.32 [@Bolger_2014] was used to remove adaptor sequence from reads and trim low-quality bases from the ends of reads:

```
java -jar trimmomatic.jar PE -phred33       \
    69XX_R1_filtered.fastq                  \
    69XX_R2_filtered.fastq                  \
    69XX_R1_filtered_adaptertrim.fastq tmp1 \
    69XX_R2_filtered_adaptertrim.fastq tmp2 \
    ILLUMINACLIP:TruSeq_both.fa:2:30:10     \
    LEADING:20 TRAILING:20                  \
    MINLEN:36
```

Sample quality check

FastQC v0.11.2 was then run to check the quality of the of the untrimmed and trimmed reads:

```
fastqc *_R1_filtered.fastq -o 69XX/QC/
fastqc *_R1_filtered_adaptertrim.fastq -o 69XX/QC/
```

Read mapping

Next, each sample was mapped to the TriTrypDB [@Aslett_2009] *L. major* Friedlin reference genome (v6.0) using TopHat v2.0.13 [@Kim_2013] (bowtie version 2.2.4).

```
tophat -o 69XX_tophat                                   \
    -g 1 -G TriTrypDB-6.0_LmajorFriedlin.gff            \
    --no-novel-juncs TriTrypDB-6.0_LmajorFriedlin_Genome \
    69XX_R1_filtered_adaptertrim.fastq                  \
    69XX_R2_filtered_adaptertrim.fastq
```

Reads were then sorted by read name using samtools v1.3 [@Li_2009]:

```
samtools sort -n 69XX_tophat/accepted_hits.bam \
    -o 69XX_tophat/accepted_hits_sorted.bam
```

Read counting

Next, HTSeq v0.6.0 [@Anders_2014] was used to quantify expression at the exon level:

```
samtools view 69XX_tophat/accepted_hits_sorted.bam |\
    htseq-count -s no -i ID                         -\
    TriTrypDB-6.0_LmajorFriedlin.gff                >\
    htseq_69XX.txt
```

Finally, sample-specific count tables were merged together to form a single comma-separated count table, which was used as input for differential expression and co-expression clustering analysis.

Reproducing this analysis

This PDF was created using the knitr and rmarkdown packages for R.

To recreate this PDF starting from the original RNA-Seq count tables, begin by using the `biocLite()` function to install the following dependencies:

- goseq
- gplots
- ggplot2
- GO.db
- RColorBrewer
- genomeIntervals
- dplyr
- limma
- preprocessCore
- venneuler
- knitr
- rmarkdown
- knitcitations

Further, you will also need to download and install the following *L. major* Friedlin annotation packages.

- Leishmania.major.Friedlin

- org.LmjF.tritryp.db
- TxDb.LmajorFriedlin.tritryp27.genes

The devtools `install_github()` function can be used for achieve this.

Next, open up an R console in the directory containing XXX.Rmd, and run:

```
library('rmarkdown')
render('XXX.Rmd', output_format='pdf_document')
```

Note that you can also generate an HTML version of the output by switching `pdf_document` for `html_document` in the function call above.

If all of the dependencies specified above are properly installed, and the versions are not significantly different from those used at the time of writing this manuscript, then you should be able to regenerate all of the tables and figures in the PDF as they appear below.

To find out which specific library versions were used, refer to the "System Information" section at the bottom of this document.

The code used in this analyses has been adopted from a more general set of R and Rmarkdown code which can be found at:

- github.com/elsayed-lab/manuscript-shared-rnaseq

References

1. Allaire,J.J., Cheng,J., Xie,Y., McPherson,J., Chang,W., Allen,J., Wickham,H., Atkins,A. and Hyndman,R. (2016) rmarkdown: Dynamic Documents for R.
2. Huber,W., Carey,V.J., Gentleman,R., Anders,S., Carlson,M., Carvalho,B.S., Bravo,H.C., Davis,S., Gatto,L., Girke,T., Gottardo,R., Hahne,F., Hansen,K.D., Irizarry,R.A., Lawrence,M., et al. (2015) Orchestrating high-throughput genomic analysis with Bioconductor. Nat. Methods, 12, 115–21.
3. Xie,Y. (2015) Dynamic Documents with {R} and knitr 2nd ed. Chapman and Hall/CRC, Boca Raton, Florida.
4. Ritchie,M.E., Phipson,B., Wu,D., Hu,Y., Law,C.W., Shi,W. and Smyth,G.K. (2015) limma powers differential expression analyses for RNA-sequencing and microarray studies. 43.
5. Kim,D., Pertea,G., Trapnell,C., Pimentel,H., Kelley,R. and Salzberg,S.L. (2013) TopHat2: accurate alignment of transcriptomes in the presence of insertions, deletions and gene fusions. Genome Biol., 14, R36.
6. Gentleman,R.C., Carey,V.J., Bates,D.M., Bolstad,B., Dettling,M., Dudoit,S., Ellis,B., Gautier,L., Ge,Y., Gentry,J., Hornik,K., Hothorn,T., Huber,W., Iacus,S., Irizarry,R., et al. (2004) Bioconductor: open software development for computational biology and bioinformatics. Genome Biol., 5, R80.
7. Anders,S., Pyl,P.T. and Huber,W. (2014) HTSeq - A Python framework to work with high-throughput sequencing data. Bioinformatics, 10.1093/bioinformatics/btu638.
8. Kanehisa,M. and Goto,S. (2000) KEGG: kyoto encyclopedia of genes and genomes. Nucleic Acids Res., 28, 27–30.
9. Ashburner,M., Ball,C.A., Blake,J.A., Botstein,D., Butler,H., Cherry,J.M., Davis,A.P., Dolinski,K., Dwight,S.S., Eppig,J.T., Harris,M.A., Hill,D.P., Issel-Tarver,L., Kasarskis,A., Lewis,S., et al. (2000) Gene ontology: tool for the unification of biology. The Gene Ontology Consortium. Nat. Genet., 25, 25–9.
10. Andrews,S. (2010) FastQC: A quality control tool for high throughput sequence data. http://www.bioinformatics.babraham.ac.uk/projects/fastqc/.
11. Smyth,G.K. (2000) Bioinformatics and Computational Biology Solutions Using R and Bioconductor,. In Gentleman,R., Carey,V.J., Huber,W., Irizarry,R.A., Dudoit,S. (eds), Solutions, Statistics for Biology and Health. Springer-Verlag, New York, Vol. 1, pp. 9–10.

12. Gilson,P.R., Nebl,T., Vukcevic,D., Moritz,R.L., Sargeant,T., Speed,T.P., Schofield,L. and Crabb,B.S. (2006) Identification and stoichiometry of glycosylphosphatidylinositol-anchored membrane proteins of the human malaria parasite Plasmodium falciparum. Mol. Cell. Proteomics, 5, 1286–99.
13. Sonnhammer,E.L., von Heijne,G. and Krogh,A. (1998) A hidden Markov model for predicting transmembrane helices in protein sequences. Proc. Int. Conf. Intell. Syst. Mol. Biol., 6, 175–82.
14. Doyle,M. a, MacRae,J.I., De Souza,D.P., Saunders,E.C., McConville,M.J. and Likić,V. a (2009) LeishCyc: a biochemical pathways database for Leishmania major. BMC Syst. Biol., 3, 57.
15. Bolger,A.M., Lohse,M. and Usadel,B. (2014) Trimmomatic: a flexible trimmer for Illumina sequence data. Bioinformatics, 30, 2114–2120.
16. Petersen,T.N., Brunak,S., von Heijne,G. and Nielsen,H. (2011) SignalP 4.0: discriminating signal peptides from transmembrane regions. Nat. Methods, 8, 785–6.
17. Law,C.W., Chen,Y., Shi,W. and Smyth,G.K. (2014) Voom: precision weights unlock linear model analysis tools for RNA-seq read counts. Genome Biol., 15, R29.
18. Hong,G., Zhang,W., Li,H., Shen,X. and Guo,Z. (2014) Separate enrichment analysis of pathways for up- and downregulated genes. J. R. Soc. Interface, 11, 20130950.
19. Bolstad,B.M., Irizarry,R.., Astrand,M. and Speed,T.P. (2003) A comparison of normalization methods for high density oligonucleotide array data based on variance and bias. Bioinformatics, 19, 185–193.
20. Zhang,B. and Horvath,S. (2005) A general framework for weighted gene co-expression network analysis. Stat. Appl. Genet. Mol. Biol., 4, Article17.
21. Young,M.D., Wakefield,M.J., Smyth,G.K. and Oshlack,A. (2010) Gene ontology analysis for RNA-seq: accounting for selection bias. Genome Biol., 11, R14.
22. Aslett,M., Aurrecoechea,C., Berriman,M., Brestelli,J., Brunk,B.P., Carrington,M., Depledge,D.P., Fischer,S., Gajria,B., Gao,X., Gardner,M.J., Gingle,A., Grant,G., Harb,O.S., Heiges,M., et al. (2010) TriTrypDB: a functional genomic resource for the Trypanosomatidae. Nucleic Acids Res., 38, D457-62.

System Information

```
if (opts_knit$get("rmarkdown.pandoc.to") == 'latex') {
    toLatex(sessionInfo())
} else {
    library('pander')
    pander(sessionInfo())
}
```

- R version 3.3.2 (2016-10-31), `x86_64-pc-linux-gnu`
- Locale: `LC_CTYPE=en_US.UTF-8`, `LC_NUMERIC=C`, `LC_TIME=en_US.UTF-8`, `LC_COLLATE=en_US.UTF-8`, `LC_MONETARY=en_US.UTF-8`, `LC_MESSAGES=en_US.UTF-8`, `LC_PAPER=en_US.UTF-8`, `LC_NAME=C`, `LC_ADDRESS=C`, `LC_TELEPHONE=C`, `LC_MEASUREMENT=en_US.UTF-8`, `LC_IDENTIFICATION=C`
- Base packages: base, datasets, graphics, grDevices, methods, parallel, stats, stats4, tools, utils
- Other packages: AnnotationDbi 1.34.4, BiasedUrn 1.07, Biobase 2.32.0, BiocGenerics 0.18.0, biomaRt 2.28.0, bitops 1.0-6, colorout 1.1-1, doParallel 1.0.10, dplyr 0.5.0, dynamicTreeCut 1.63-1, fastcluster 1.1.22, flashClust 1.01-2, foreach 1.4.3, geneLenDataBase 1.8.0, GenomeInfoDb 1.8.7, GenomicFeatures 1.24.5, GenomicRanges 1.24.3, ggplot2 2.2.0, GO.db 3.3.0, goseq 1.24.0, gplots 3.0.1, gridExtra 2.2.1, hpgltools 2016.02, IRanges 2.6.1, iterators 1.0.8, knitcitations 1.0.7.1, knitr 1.15.1, Leishmania.major.Friedlin 28.0, limma 3.28.21, Matrix 1.2-7.1, nvimcom 0.9-25, OrganismDbi 1.14.1, org.LmjF.tritryp.db 28.0, preprocessCore 1.34.0, RColorBrewer 1.1-2, RCurl 1.95-4.8, readr 1.0.0, reshape2 1.4.2, rmarkdown 1.2, rtracklayer 1.32.2, S4Vectors 0.10.3, TxDb.LmajorFriedlin.tritryp28.genes 28.0, WGCNA 1.51
- Loaded via a namespace (and not attached): acepack 1.4.1, assertthat 0.1, backports 1.0.4, base64 2.0, bibtex 0.4.0, BiocInstaller 1.22.3, BiocParallel 1.6.6, Biostrings 2.40.2, caTools 1.17.1,

cluster 2.0.5, codetools 0.2-15, colorspace 1.3-1, data.table 1.10.0, DBI 0.5-1, digest 0.6.10, evaluate 0.10, foreign 0.8-67, Formula 1.2-1, gdata 2.17.0, GenomicAlignments 1.8.4, graph 1.50.0, grid 3.3.2, gtable 0.2.0, gtools 3.5.0, highr 0.6, Hmisc 4.0-1, htmlTable 1.7, htmltools 0.3.5, httr 1.2.1, impute 1.46.0, KernSmooth 2.23-15, labeling 0.3, lattice 0.20-34, latticeExtra 0.6-28, lazyeval 0.2.0, lubridate 1.6.0, magrittr 1.5, matrixStats 0.51.0, memoise 1.0.0, mgcv 1.8-15, munsell 0.4.3, nlme 3.1-128, nnet 7.3-12, openssl 0.9.5, plyr 1.8.4, R6 2.2.0, RBGL 1.48.1, Rcpp 0.12.8, RefManageR 0.13.1, RJSONIO 1.3-0, rpart 4.1-10, rprojroot 1.1, Rsamtools 1.24.0, RSQLite 1.1, scales 0.4.1, splines 3.3.2, stringi 1.1.2, stringr 1.1.0, SummarizedExperiment 1.2.3, survival 2.40-1, tibble 1.2, XML 3.98-1.5, XVector 0.12.1, yaml 2.1.14, zlibbioc 1.18.0
